# Structural and mechanistic insights into primer synthesis initiation by DNA primase

**DOI:** 10.1038/s41467-026-74862-8

**Published:** 2026-07-03

**Authors:** Pengzhi Wu, Fred F. Damberger, Johannes Zehnder, Nina Wehr, Niklas Senning, Georg Lipps, Thomas Wiegand, Frédéric H.-T. Allain

**Affiliations:** 1 Department of Biology, Institute of Biochemistry, ETH Zürich, Switzerland; 2 Laboratory of Physical Chemistry, ETH Zürich, Switzerland; 3 Institute of Technical and Macromolecular Chemistry, RWTH Aachen University, Aachen, Germany; 4 Max Planck Institute for Chemical Energy Conversion, Mülheim (Ruhr), Germany; 5 Institute of Chemistry and Bioanalytics, University of Applied Sciences Northwestern Switzerland, Muttenz, Switzerland; 6 Present Address: State Key Laboratory of Chemical Biology, Shanghai Institute of Organic Chemistry, Chinese Academy of Sciences, Shanghai, China

**Keywords:** Solution-state NMR, Enzyme mechanisms, DNA synthesis

## Abstract

DNA primases synthesize short primers required for genome replication, yet the mechanism of initial dinucleotide formation remains poorly understood. Here, we investigate the primase encoded by the pRN1 plasmid from the thermoacidophile archaeon *Sulfolobus islandicus*, a minimal model for primer synthesis. Using nucleotide analogues to slow the reaction, we capture transient intermediates of dinucleotide formation. Structural NMR and modeling reveal that the ancillary domain simultaneously binds the DNA template and two initiating nucleotides. Unexpectedly, only the second nucleotide base-pairs with the template, whereas the first remains unpaired, inducing template-base flipping and linker interaction. This interaction promotes a closed conformation in which the second nucleotide moves from the initiation to the elongation site and the first forms a base pair in the initiation site, positioning both nucleotides for catalysis. These findings reveal a mechanism for template recognition, nucleotide assembly, and proofreading during primer initiation that is likely conserved among primases.

## Abstract (web summary)

DNA primases synthesize short primers that allow DNA polymerases to initiate genome replication, yet the mechanism of primer initiation remains unclear. Here, the authors show how an ancillary domain assembles and proofreads the first two nucleotides, ensuring efficient and accurate dinucleotide formation.

## Introduction

DNA primases synthesize short oligonucleotides, typically 10–15 nucleotides long, which are used by DNA polymerases to initiate DNA synthesis^1–3^. Primases are essential for both leading and lagging strand synthesis, as DNA polymerases cannot initiate strand synthesis de novo. At the replication fork, primases periodically synthesize RNA primers on the lagging strand, facilitating the discontinuous synthesis of Okazaki fragments^4–7^. These RNA primers are later removed by ribonuclease and replaced by DNA, but their initial synthesis is crucial for the replication process^8^. The catalytic mechanism of DNA primases involves three key stages: initiation, elongation, and termination^9^. During initiation, primases bind two initiating nucleoside triphosphates (NTPs)—one at the initiation site (I-site) and the other at the elongation site (E-site)^10^. The 3’-hydroxy group of the first NTP then attacks the α-phosphate of the second NTP, forming a dinucleotide that becomes the 5′ end of the oligonucleotide primer^11^. However, the structural mechanism by which primases recruit the two NTPs and position the 3’-hydroxy group for efficient nucleophilic attack remains unclear.

DNA primases are classified into two main types: DnaG-type primases, primarily found in bacteria, and archaeo-eukaryotic primases, present in archaea and eukaryotes^12^. DnaG primases function as monomers and contain a catalytic domain with a topoisomerase-primase (TOPRIM) fold, flanked by zinc-binding and helicase interaction domains^13,14^. In contrast, archaeo-eukaryotic primases typically function as heterodimers, consisting of a small catalytic subunit (PriS) and a large ancillary subunit (PriL). Their catalytic domain is characterized by an N-terminal α/β fold and a C-terminal RNA recognition motif (RRM)-like fold^15–17^. The ancillary subunit plays a crucial role in regulating primase activity and facilitating the transfer of the synthesized primer to DNA Polymerase α^18^.

**Fig. 1:**
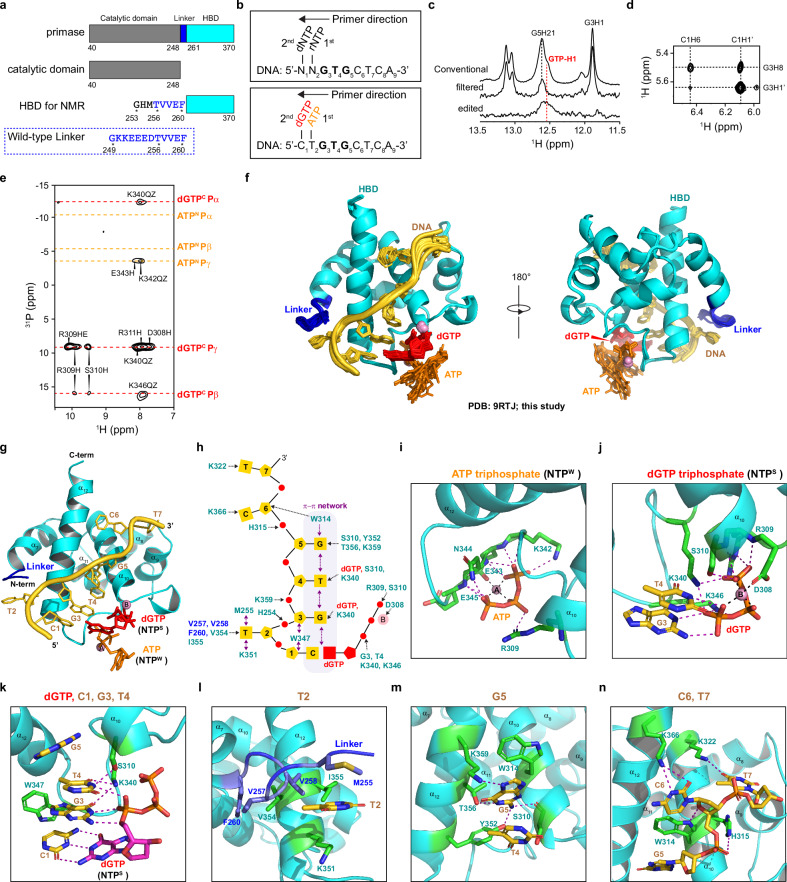
Binding of two cognate NTPs to the HBD results in a single base-pair formation. **a** Primase constructs from the pRN1 plasmid of the thermoacidophile archaeon *Sulfolobus islandicus* used in this study: primase (residues 40–370), catalytic domain (residues 40–248), and the HBD construct for NMR, including the HBD (residues 261–370), five linker residues (residues 256–260), and three additional non-native residues (GHM, residues 253–255) remaining after TEV cleavage. **b** DNA template (DNA^N1N2^) and cognate NTPs; the commonly used DNA^CT^ sequence with its initiating NTPs is shown. **c** 1D ^1^H NMR spectra of the HBD-DNA^CT^-ATP-GTP complex at pH 6.3 and 278 K. Only GTP is ^13^C- and ^15^N-labeled. **d** NOE cross-peaks between C1 and G3 in the 2D F1f-F2f NOESY spectrum of the HBD-DNA^CT^-ATP^C^-dGTP^C^ complex. Only protein is ^13^C- and ^15^N-labeled. **e** 2D hPH solid-state NMR spectrum of primase^H145A^-DNA^CT^-ATP^N^-dGTP^C^. Cross-peaks from ATP^N^ (orange) and dGTP^C^ (red) are indicated. Q denotes a pseudo-atom representing three equivalent protons; for example, K340QZ refers to the three equivalent protons attached to the NZ atom of K340. **f** Ensemble of the HBD-DNA^CT^-ATP-dGTP structures. **g** Representative structure of the complex. Pink spheres indicate Mg^2+^ ions. Nucleotide C8 and A9 of the DNA template are omitted for clarity, as they are dynamic and do not form stable interactions with the protein. **h** Schematic of protein-DNA and protein-dGTP interactions; hydrogen bonds are observed in at least half the conformers. **i**–**n** Close-up views of binding pockets, highlighting hydrogen bonding, stacking, and hydrophobic contacts at NTP^W^, NTP^S^, and DNA interaction sites.

The pRN1 plasmid from *Sulfolobus islandicus* harbors a bifunctional enzyme, ORF904, which combines primase and DNA polymerase activities within its N-terminal region, and helicase activity in the C-terminal region^19^. The pRN1 primase is one of the archaeo-eukaryotic primases that contains a catalytic N-terminal domain in *cis* with a C-terminal ancillary domain (Fig. 1a). The ancillary regulatory domain is a helix bundle domain (HBD) that shares homology with the C-terminal region of the eukaryotic PriL (PriL-CTD). Deletion of this domain abolishes primase initiation but does not affect elongation^20^. In the absence of bound DNA and nucleotides, the two domains do not interact with each other and remain in an open conformation, connected by a flexible interdomain linker^21,22^. Priming by pRN1 primase occurs exclusively at a GTG motif in the DNA template located immediately downstream of the initiation site. Additionally, the primase synthesizes a primer with a ribonucleotide at the first position, followed by deoxyribonucleotides from the second position onward, a dinucleotide composition which is found in many organisms, including the human PrimPol or the CRISPR-associated primase-polymerase from *Marinitoga* sp.1137 (MsCAPP)^23–25^. We previously revealed how the HBD of the pRN1 primase uses the binding of two NTP molecules to achieve sequence-specific recognition of the template GTG sequence^22^.

Several primase structures have been reported, including unbound forms lacking DNA and nucleotides^18,26^, as well as complexes with a single NTP^27,28^ or with chimeric DNA-RNA template-primers^29–32^. Notably, a recent study revealed the structure of the catalytic domain of MsCAPP bound to two base pairs formed by the first two NTPs. Although MsCAPP contains both a catalytic and an ancillary domain, this structure included only the catalytic domain, leaving the essential regulatory role of the ancillary domain unresolved^24^. Furthermore, there is currently no structural information on how the catalytic and ancillary domains of MsCAPP coordinate during primer synthesis. Although that study concluded that the catalytic domain alone is sufficient for dinucleotide formation in vitro, both domains of archaeal-eukaryotic primases are required under physiological conditions^20,33,34^.

In this study, we reveal mechanistically how the pRN1 primase sequentially prepares the first two NTPs for dinucleotide formation using structural NMR spectroscopy. We present three structural models that capture distinct stages of this process. One structure is determined entirely from experimental NMR restraints, while the other two are hybrid models generated by integrating NMR-derived restraints with crystallographic coordinates. Together, these structures reveal how the catalytic and ancillary domains coordinate to ensure substrate preparation, proofreading of the nucleotides and positioning of the NTPs for efficient dinucleotide synthesis. This mechanism is likely general among most known primases, highlighting the broad significance of the proposed mechanism.

## Results

### Binding of two cognate NTPs to the HBD results in a single base-pair formation

The DNA^N1N2^ template used in this work follows the sequence 5’-N_1_N_2_G_3_T_4_G_5_C_6_T_7_C_8_A_9_-3’, where N1 and N2 can be any nucleotide (Fig. 1b). N1 corresponds to the template base that pairs with the second incoming nucleotide, while N2 serves as the template for pairing with the first NTP. Our previous work demonstrated that the HBD of pRN1 primase binds both ATP and dGTP, with dGTP specifically binding to the DNA^CT^ template^35^. This strongly suggests that both nucleotides would base pair with the first two positions (N1 and N2) of the DNA^N1N2^ template within the HBD. Here with DNA^CT^, one would expect the formation of two base-pairs C1-dGTP and T2-ATP (Supplementary Fig. 1a). To investigate this, we added ^13^C,^15^N isotope-labeled GTP to the unlabeled HBD-DNA^CT^-2ATP complex and collected 1D ^1^H-filtered/edited solution-state NMR spectra (Fig. 1c). We used labeled GTP instead of labeled dGTP because the HBD does not distinguish between rNTP and dNTP (Supplementary Fig. 1b). A distinct imino proton signal from GTP confirmed the formation of a base pair with C1, as this signal indicates that the guanine imino proton is stabilized by hydrogen bonding-an interaction characteristic of a structured, paired state and absent in free nucleotide. We then prepared the ^13^C,^15^N-labeled HBD-DNA^CT^-ATP^C^-dGTP^C^ complex using NTP analogues to ensure stability of the complex for over a month, since NTPs hydrolyze rapidly, preventing long NMR experiments required for resonance assignment and structural characterization. NTP analogs where the oxygen atom between the β- and γ-phosphates is replaced by nitrogen or carbon are referred to herein as NTP^N^ and NTP^C^, respectively^35^. Unexpectedly, NOE signals supported stacking of C1 with G3 (Fig. 1d), while T2 interacts with the interdomain linker residues from H254 to E259 (Supplementary Fig. 1c, d). These results confirm that ATP does not base pair with T2, and that only a single base pair, C1-dGTP, forms in the HBD quaternary complex.

### Mapping the two NTP binding sites in the HBD

NMR studies of the HBD-DNA^CT^-2ATP complex revealed that the HBD can simultaneously bind two ATP molecules, but with markedly different affinity. One ATP binds tightly at the high-affinity NTP^S^ site (strong binding; residues D308-R311; Supplementary Fig. 1d), while the second binds weakly at the low-affinity NTP^W^ site (weak binding; residues K340-N344)^22^. To determine the binding positions of ATP and dGTP in the HBD-DNA^CT^-ATP-dGTP complex, we employed both solution- and solid-state NMR. For solid-state NMR, we used a catalytically inactive full-length primase mutant (primase^H145A^) to increase molecular weight, which facilitated sedimentation^35^. Our solution-state NMR data confirmed that ATP and dGTP analogues bind identically to the free HBD or the HBD fused to the inactive catalytic domain (Supplementary Fig. 1e, f). We next turned to solid-state NMR allowing the acquisition of ^1^H-^31^P correlation spectra (hPH, using two cross-polarization transfers)^36,37^. By performing the experiments at a MAS frequency of 100 kHz, proton-detected experiments become feasible due to the significant narrowing of the ^1^H resonances^38–42^. The hPH solid-state NMR spectrum of primase^H145A^-DNA^CT^-ATP^N^-dGTP^C^ (Fig. 1e) surprisingly revealed that ATP^N^ binds at the NTP^W^ site, while dGTP^C^ occupies the NTP^S^ site, contradicting our initial hypothesis (Supplementary Fig. 1a). Hydrogen bonding involving the side-chain amino groups of three lysine residues (K340, K342, and K346) stabilizes the bound states of both NTPs, as evidenced by the observation of these typically fast-exchanging side-chain signals in the 2D ^1^H-^15^N HMQC solution-state NMR spectrum (Supplementary Fig. 1g). Additionally, when comparing the solution-state NMR spectra of HBD-DNA^CT^-ATP in the presence of dGTP or GTP^C^, we observed chemical shift changes at the NTP^S^ site confirming this location for the guanine (Supplementary Fig. 1h).

Together, these findings demonstrate that dGTP displaces ATP at the NTP^S^ site to base-pair with C1, whereas the second ATP remains bound at the NTP^W^ site without base-pairing with T2, which instead interacts with the interdomain linker (Supplementary Fig. 1i). We define the HBD-DNA^CT^-2ATP complex, which lacks base-pairing, as the 0bp_HBD state, and the HBD-DNA^CT^-ATP-dGTP complex, with a single base pair, as the 1bp_HBD state. All NMR complexes used throughout the manuscript, along with their corresponding conformational states and associated figure numbers, are summarized in Supplementary Fig. 2.

### Hybrid solution- and solid-state NMR structure of HBD-DNA^CT^-ATP-dGTP

To elucidate how the HBD binds two nucleotides and forms a single base pair, we determined the NMR structure of the HBD-DNA^CT^-ATP^C^-dGTP^C^ complex (PDB: 9RTJ) in this study. This resulted in a well-defined structural ensemble (Fig. 1f, g and Supplementary Table 1). Key interactions between the protein, DNA, and dGTP are summarized in Fig. 1h. At the NTP^W^ site, ATP’s triphosphate interacts with residues K342-E345 and coordinates a magnesium ion (MgA) through E343 (Fig. 1i). However, the ribose and base of ATP remain flexible, without interacting with the HBD or forming a base pair with T2 (Fig. 1g). At the NTP^S^ site, dGTP’s triphosphate forms hydrogen bonds with R309, S310, K340, and K346, and G3 and T4 of the DNA template, while coordinating a second magnesium ion (MgB) via D308 (Fig. 1j). The base of dGTP forms a Watson-Crick pair with C1, stabilized by π-π stacking with G3 (Fig. 1k). The solvent-exposed ribose groups of ATP and dGTP are consistent with the HBD’s inability to distinguish between rNTP and dNTP (Supplementary Fig. 1b).

T2 is bulged out and interacts with the interdomain linker and helix 12, supported by stacking between M255 and K351, along with hydrophobic contacts involving residues V257, V258, F260, V354, and I355 (Fig. 1l). G3 and T4 are stacked between C1 and G5 (Fig. 1k), with the first two interacting with the dGTP triphosphate (Fig. 1j). G3 additionally engages in T-shaped stacking with W347, resulting in a very unusual upfield ^1^H chemical shift value for G3 H8 (~5.5 ppm), beyond the current range for Guanine H8 in the BMRB chemical shift database (Fig. 1d). G5 is stabilized by hydrogen bonds with S310, Y352, T356, and K359, as well as stacking with T4 and W314 (Fig. 1m). Continuous stacking of C1, the GTG motif, and W314 enhances the structural stability of the complex (Fig. 1h). Additionally, the phosphate, ribose and base groups of C6 form hydrogen bonds with H315, W314, and K366, respectively, while T7 interacts with K322 (Fig. 1n). Previous mutagenesis studies of the residues involved in these protein-DNA and protein-NTP interactions confirm their importance for DNA template binding and primase activity in the primase, consistent with our current structure^22^.

### Conformational changes upon single base-pair formation

**Fig. 2:**
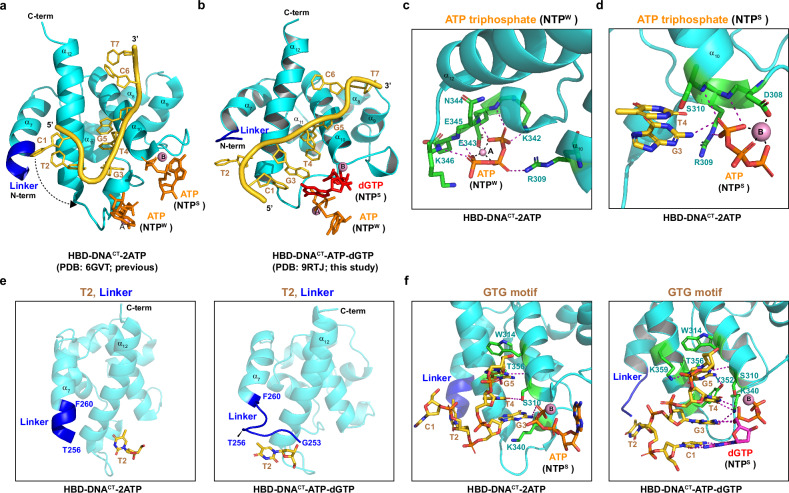
Conformational changes upon single base-pair formation. **a** NMR structure of HBD-DNA^CT^–2ATP before base-pair formation (PDB: 6GVT). **b** NMR structure of HBD-DNA^CT^-ATP-dGTP after C1-dGTP base-pair formation. **c** ATP triphosphate binding pocket in the NTP^W^ binding site of HBD-DNA^CT^–2ATP. **d** ATP triphosphate binding pocket in the NTP^S^ binding site of HBD-DNA^CT^–2ATP. **e** Relative position of T2 and the Linker in HBD-DNA^CT^–2ATP (left) compared to HBD-DNA^CT^-ATP-dGTP (right). **f** Structural comparison of the GTG motif between HBD-DNA^CT^-2ATP (left) and HBD-DNA^CT^-ATP-dGTP (right).

To understand the molecular mechanism underlying C1-dGTP base-pair formation, we compared the NMR structures of the HBD-DNA^CT^-2ATP complex, previously reported (PDB: 6GVT), and HBD-DNA^CT^-ATP-dGTP complex, newly determined in this study (PDB: 9RTJ) (Fig. 2a, b and Supplementary Movie 1). ATP maintains a consistent position at the NTP^W^ site in both complexes. However, its Pα shifted to interact with N344 instead of K346 upon C1-dGTP base-pair formation (Figs. 1i, 2c). This repositioning likely occurred as K346 engaged the dGTP triphosphate at the NTP^S^ site (Fig. 1j).

The major structural transition occurs at the NTP^S^ site, where ATP is replaced by dGTP (Fig. 2a, b). In the 2ATP complex, C1 was distant from both NTP binding sites. Upon dGTP binding, C1 was repositioned beneath G3 to form a base pair with dGTP. In this configuration, C1 forms three canonical hydrogen-bonds with dGTP, engages in π-π stacking with G3, and adopts a T-shaped stacking interaction with W347 (Fig. 1k). Furthermore, T2 now interacts strongly with the interdomain linker (Figs. 1l, 2e). The GTG motif also exhibits more interactions following base-pair formation (Fig. 2f). In particular, five new hydrogen bonds are formed, enhancing HBD’s specific recognition of the GTG motif by approximately five-fold (Supplementary Fig. 3). Very importantly, the substantial conformational shift in T2 and its interaction with the interdomain linker unfolds the first helical turn of helix α7 (Fig. 2e, amino acids T256-E259). Despite the unfolding event, dGTP binding to the HBD remains enthalpically favorable, accounting for its approximately five-fold higher binding affinity compared to ATP. This stronger affinity arises from the aforementioned enhanced molecular interactions and the resulting conformational stabilization.

### The HBD-formed base pair is initially recognized by the catalytic domain’s I-site

We next asked if the formation of a single C1-dGTP base pair within the HBD constitutes a necessary intermediate during primase synthesis. When ATP and dGTP were used as substrates with the DNA^CT^ template, dinucleotide formation proceeded too rapidly to detect a single base-pair intermediate by NMR. To stall the reaction, we substituted ATP with 3’-dATP, eliminating the reactive 3’-OH group. This resulted in a stable complex that could not proceed to catalysis. Yet large chemical shift changes are observed in both the HBD and the catalytic domain, indicating that a complex with two base-pairs is now formed with both domains contacting the DNA template and the nucleotides (2bp_closed state; Supplementary Fig. 4a, b). Notably, the G3 imino proton signal in this state exhibits a significant shift relative to the 1bp_HBD state (Supplementary Fig. 4c), indicating that G3 now stacks on the T2-ATP base pair rather than the C1-dGTP pair. The shift thus reflects the transition from one to two base-pairs.

**Fig. 3:**
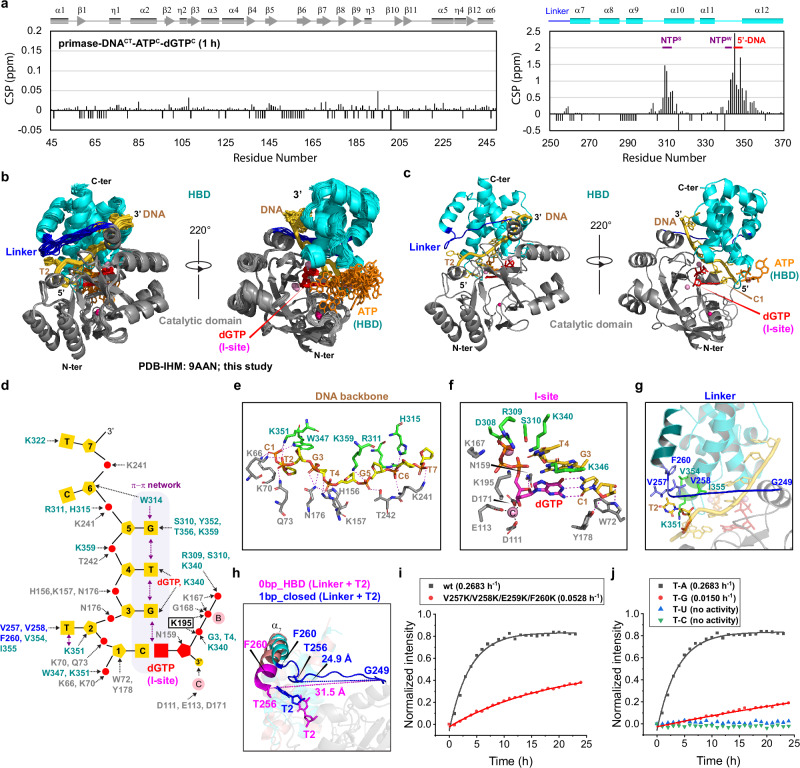
The HBD-formed base pair is initially recognized by the catalytic domain’s I-site. **a** Chemical shift perturbations (CSP) of the primase-DNA^CT^ complex upon ATP^C^ and dGTP^C^ binding after a 1-h reaction. Left, CSPs in the catalytic domain; right, CSPs in the Linker and HBD. **b** Ensemble of primase-DNA^CT^-1bp complex structures. **c** Lowest-energy structure of the complex. **d** Schematic of protein-DNA and protein-dGTP contacts. **e** Close-up of protein-DNA backbone interactions. **f** Binding pocket of the C1-dGTP base pair. **g** Linker interaction with T2 of the DNA template. **h** Conformations of the Linker and helix 7 in the 0bp_HBD and 1 bp_closed states, highlighting T2-Linker contacts. In 0bp_HBD (HBD-DNA^CT^-2ATP), the HBD is salmon, Linker and T2 magenta. In 1bp_closed (primase-DNA^CT^-1bp), the HBD is cyan, Linker and T2 blue; structures aligned on the HBD. **i** Time-resolved ^31^P NMR monitoring dinucleotide formation for wild-type primase (black) and Linker mutant V257K, V258K, E259K and F260K (red); rate constants (*k*, h^−^^1^) are indicated. **j** Dinucleotide formation with a canonical Watson-Crick at the E-site and different base pair configurations at the I-site using primase-DNA^CT^-dGTP with ATP (black), GTP (red), UTP (blue), or CTP (green).

To directly capture the single base-pair intermediate, we next assembled a primase-DNA^CT^ complex with the NTP analogues ATP^C^ and dGTP^C^. At early time points, NMR spectra revealed the presence of a single C1-dGTP base pair within the HBD (1bp_open state), which subsequently transitioned into a two-base-pair conformation (Fig. 3a and Supplementary Fig. 4c, d). These results strongly support the conclusion that formation of the single base pair in the HBD is a required intermediate for dinucleotide synthesis.

We next sought to elucidate how this base pair is transferred from the HBD to the catalytic domain. The crystal structure of MsCAPP primase containing two base pairs in the active site revealed two distinct NTP binding pockets: the I-site and E-site (Supplementary Fig. 4e)^24^. To identify which site first engages the C1-dGTP base pair, we performed paramagnetic relaxation enhancement (PRE) experiments using the primase–DNA^CT^–dGTP^C^ complex in the presence of paramagnetic manganese(II) ions in place of magnesium(II) ions^43^. We observed significant line broadening in resonances corresponding to the I-site, indicating close spatial proximity to the paramagnetic Mn^2+^ center (Supplementary Fig. 4f). In contrast, residues in the E-site showed no such broadening. These findings indicate that the I-site, rather than the E-site, initially recognizes the C1-dGTP base pair, forming the 1bp_closed state (Supplementary Fig. 4g).

To further characterize the 1 bp_closed state, we prepared a complex containing the primase, DNA^CT^ and 2GTP (Supplementary Fig. 5a, b). This composition allows formation of a C1-GTP base pair in the I-site but prevents occupancy of the E-site, which selectively binds dNTP. We could observe NMR chemical shift perturbations in the catalytic domain and DNA template in comparison to the open conformation (Supplementary Fig. 5c, d). We attribute these chemical shift changes to dynamic equilibrium between the 1 bp_open and 1 bp_closed states, with the open form predominating. We define this intermediate ensemble as 1bp_exchange state.

Together, these results provide the evidence that the single base pair formed in the HBD acts as a transient intermediate that is subsequently transferred to and recognized by the catalytic domain’s I-site. We refer to this intermediate, where the base pair is simultaneously bound by both the HBD and I-site, as the primase–DNA^CT^–1bp complex, which adopts the 1bp_closed conformation.

### Structural model of the primase-DNA^CT^-1bp complex based on NMR data

Although recent studies have captured preinitiation and elongation-related states of human primase and proposed an initiation model for the priming complex^17,44–46^, the transient closed intermediate associated with formation and handover of the first base pair remains structurally uncharacterized. To gain structural insight into this early closed intermediate, we generated a testable 3D model of the primase-DNA^CT^-1bp complex by integrating our NMR data with previously determined crystal structures.

This model was guided by two key observations. First, we had determined the NMR structure of the HBD bound to DNA^CT^ and the two cognate NTPs in a state containing a single base pair (Fig. 1g). Second, PRE data showed that the C1-dGTP base pair occupies the I-site of the catalytic domain (Supplementary Fig. 4f). Based on these observations, we positioned the C1-dGTP base pair in the I-site to generate a structural model of this transient closed intermediate. Model building involved CYANA regularization using data obtained from different complexes, followed by CYANA structure calculation and AMBER refinement guided by our NMR analysis (Supplementary Fig. 5e, f and Supplementary Method). The latter two steps were carried out using procedures similar to those used in standard NMR structure determination.

To clarify the experimental basis of this model, restraints for the HBD, linker, ATP, the dGTP triphosphate, and DNA template residues T2-A9 were taken from the NMR structure of the HBD-DNA^CT^-ATP-dGTP complex, supported by the nearly identical chemical shifts observed for these components in the two complexes (Fig. 3a and Supplementary Fig. 1e). Because many residues in the catalytic domain, particularly near the active site, were broadened beyond detection and could not be assigned (Fig. 3a), the catalytic domain was modeled using the pRN1 crystal structure (PDB: 3M1M). The position of the C1-dGTP base pair in the I-site was guided by PRE data. The geometry of this base pair, excluding the dGTP triphosphate, was modeled based on the corresponding base pair observed in the MsCAPP structure (PDB: 7QAZ).

The resulting model provides the first structural insights into how the HBD and catalytic domain adopt a closed conformation to coordinate the formation and handover of the first base pair (Fig. 3b–d and Supplementary Table 1). Domain closure is mediated by interactions between the protein and nucleic acids (Fig. 3d). The phosphate backbone of the DNA is simultaneously engaged by both domains, bringing them into proximity as both domains bind different sides of the DNA (Fig. 3e), while the ribose and base moieties of the DNA—except for the first cytosine—are bound exclusively by the HBD. Similarly, the C1-dGTP base pair is co-bound at the I-site: the two domains interact with the dGTP’s triphosphate, and the catalytic domain additionally contacts the sugar (Fig. 3f). Two metal ions, one bound by each domain, assist in stabilizing this intermediate. This clearly shows that after initial binding to the HBD, the DNA template and the cognate NTPs are accessible to the catalytic domain. What is remarkable is that upon formation of the single base pair, the interdomain linker is extended due to the contacts with T2 (Fig. 3g). The extended linker allows the two domains to bind DNA and the cognate NTPs together. In contrast, when we generated independent models of the primase-DNA^CT^-2ATP complex in the absence of a base pair using different structural approaches (Fig. 3h and Supplementary Fig. 6a), the interdomain linker was too short to allow binding between the two domains in any model. This suggests that formation of the C1-dGTP base pair within the HBD acts as a proofreading checkpoint for the catalytic domain recruitment.

In agreement with this model, our previous study showed that mutation of K351 to alanine (K351A), which stacks with T2 and stabilizes its interaction with the linker (Fig. 1l), completely abolishes dinucleotide formation by disrupting both DNA and NTP binding^22^. In the present study, we additionally introduced mutations in the interdomain linker (V257-F260) that disrupt both α-helical formation and hydrophobic interactions with T2, which significantly reduces primase activity (Fig. 3i). These findings are also consistent with previous studies using an HPLC-based primase assay, which showed that linker length is critical for the primase synthesis, as shortening the flexible linker by deletion of residue 250 reduces primer activity to approximately 25% of the wild-type level^21^. The agreement with this independent biochemical assay supports the structural relevance of the effects observed in our NMR-based priming experiments. More broadly, the two approaches are complementary: HPLC-based assays define primer synthesis activity and product distribution, whereas the NMR-based assay enables direct monitoring of substrate binding, intermediate formation, and mechanistic transitions during dinucleotide synthesis at atomic resolution.

In forming the closed complex model, we could observe two hydrogen bonds between side-chains from the two domains: D194-R309 and G166-R311 (Supplementary Fig. 6b). However, mutation of D194 to alanine (D194A) did not affect primase activity, indicating that the D194-R309 interaction is not essential for domain closure (Supplementary Fig. 6c). Instead, precise Watson-Crick base pairing and recognition by the I-site are critical for domain closure. Mutations in the I-site residues that interact with the C1-dGTP base pair abolish primase activity (Supplementary Fig. 6d), and mismatched base pairs at this position prevent stable complex formation and inhibit dinucleotide synthesis (Fig. 3j).

### Transition from single- to double-base-pair binding

For the dinucleotide formation to take place, the C1-dGTP base pair must move from the I-site to the E-site, while a new T2-ATP base pair forms and occupies the I-site. This configuration defines a pre-catalytic state referred to here as the primase-DNA^CT^-2bp complex (Supplementary Fig. 7a). To study this two-base-pairs state, we analyzed two states of the catalytic cycle: a 2bp_closed state and a dinucleotide_closed state. The 2bp_closed state was shown earlier using dGTP and the ATP analog 3′-dATP (Supplementary Fig. 4a, b). The dinucleotide_closed state was prepared by incubating the primase with DNA^CT^, ATP and dGTP, leading to rapid formation of the pppApG dinucleotide before NMR data collection.

In both states, we still see large chemical shift changes in the HBD domain, indicating that it remains engaged with the DNA template and interacts with the ATP triphosphate in the 2bp_closed state or the dinucleotide in the dinucleotide_closed state (Supplementary Figs. 4a, 7b). Additionally, large chemical shift changes in residues at both the I and E sites of the catalytic domain confirm occupation by the expected two base-pairs. Supporting the presence of the two base pairs, the imino protons of G3 and T4 exhibited large chemical shift changes relative to the 1bp_HBD state observed at the beginning of the reaction (Supplementary Fig. 4c). These shifts suggest that G3 stacks over T2, rather than C1. Furthermore, the solid-state hPH NMR spectrum of the primase-DNA^CT^-pNHppApG complex revealed that the triphosphate moiety of the dinucleotide pNHppApG—derived from the ATP analogue ATP^N^—has shifted from the NTP^W^ site to the NTP^S^ site of the HBD following catalysis (Supplementary Fig. 7c). This transition is supported by pre-reaction cross-peaks between the Pγ atom of ATP^N^ and NTP^W^ site residues (Fig. 1e and Supplementary Fig. 7d), which shift to interaction with NTP^S^ site residues after catalysis.

To further monitor this transition, we conducted time-resolved NMR spectroscopy using R311 in the HBD as a reporter for the NTP^S^ site, which also reflects occupancy of the I-site in the catalytic domain. Upon addition of ATP or its analogue ATP^C^ to the primase-DNA^CT^ complex, distinct chemical shift values for R311 indicated binding of either ATP species (Supplementary Fig. 7e). Introduction of dGTP^C^ to the primase-DNA^CT^-2ATP complex led to ATP displacement from the NTP^S^ site and formation of the C1-dGTP^C^ base pair, as evidenced by R311 transitioning to the analogue-binding position. As the reaction progressed, ATP reoccupied the NTP^S^ site, pairing with T2 at the I-site, while the C1-dGTP^C^ base pair migrated to the E-site. By the end of the reaction, the C1-dGTP^C^ base pair occupies the E-site, and the T2-ATP base pair is positioned in the I-site. These data clearly confirmed a coordinated exchange of nucleotide positions between the I-site and the E-site of the catalytic domain during dinucleotide formation, revealing a catalysis-coupled transition of ATP from the NTP^W^ site to the NTP^S^ site of the HBD (Supplementary Fig. 7a).

### Structural model of the primase-DNA^CT^-2bp complex based on NMR data

**Fig. 4:**
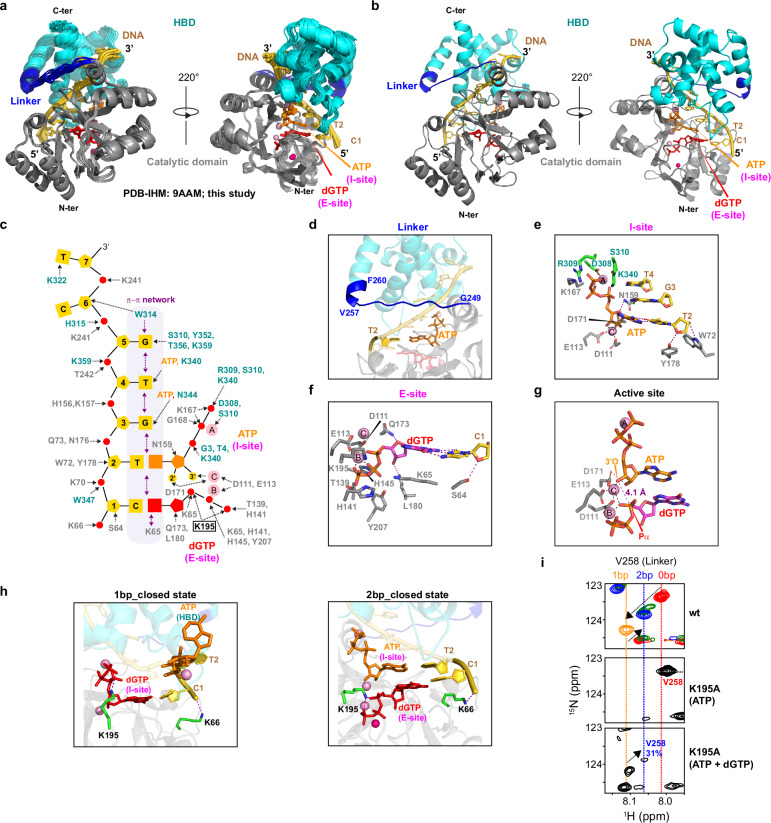
Structural model of the primase-DNA^CT^-2bp complex based on NMR data. **a** Ensemble structures of the primase-DNA^CT^-2bp complex. Magnesium ions are shown as pink spheres, and the zinc ion is shown as a hot pink sphere. **b** Lowest-energy structure of the primase-DNA^CT^-2bp complex. **c** Schematic representation of protein-DNA and protein-nucleotide contacts. **d** Linker region showing a reformed helical conformation at its C-terminal portion. **e** Close-up of the T2-ATP base pair binding pocket in the I-site. **f** Close-up of the C1-dGTP base pair binding pocket in the E-site. **g** Pre-catalytic conformation of the active site, showing alignment of ATP’s 3’-OH group for nucleophilic attack on the α-phosphate of dGTP. **h** Structural comparison of K195 and K66 interactions with dGTP and C1 in the primase-DNA^CT^-1bp complex (1bp_closed state, left) and primase-DNA^CT^-2bp complex (2bp_closed state, right), suggesting their role in base pair transfer. **i** Chemical shift changes of V258 in the Linker during reaction stages. Top, overlaid 2D ^1^H-^15^N BEST-TROSY spectra of primase-DNA^CT^ complex with ATP (red, 0 bp), dGTP (orange, 1 bp), 3’-dATP and dGTP (green, 2 bp), and pppApG dinucleotide (blue, 2 bp). Middle and bottom, V258 shifts in primase^K195A^-DNA^CT^-2ATP before and after dGTP addition (40-min reaction at 313 K). Dashed arrows indicate shift changes.

To gain insights into the 2 bp_closed state, we constructed a structural model of the primase-DNA^CT^-2bp complex, using the same approach as for the primase-DNA^CT^-1bp complex (Fig. 4a–c and Supplementary Table 1). Distance restraints for the HBD and DNA^CT^ template residues G3-A9 were taken from our NMR structure of the HBD-DNA^CT^-ATP-dGTP complex, supported by the nearly identical chemical shifts observed in the two complexes (Supplementary Figs. 1e, 4a). Restraints for the catalytic domain were derived from the crystal structure of pRN1 primase (PDB: 3M1M). The geometries of the C1-dGTP and T2-ATP base pairs, excluding the ATP triphosphate, were modeled based on the corresponding interactions observed at the I-site and E-site of the MsCAPP structure (PDB: 7QAZ). The ATP triphosphate was positioned using distance restraints from solid-state NMR spectra of pRN1 primase in complex with DNA^CT^ and the pNHppApG dinucleotide (Supplementary Fig. 7c). Thus, the 1bp_closed and 2bp_closed states are represented by NMR-guided structural models that are strongly supported by experimental data, although some mechanistic details remain model-based because full NMR structures of the entire complexes could not be directly determined.

The resulting model closely resembles the MsCAPP catalytic domain structure containing two base pairs in the active site but differs in the DNA π-π stacking network (Supplementary Fig. 8). In the pRN1 model, G3 is positioned between T2 and ATP, which is co-bound by the catalytic domain and the HBD, thereby forming a π-π stacking network with both bases. This arrangement is consistent with experimental observations from our previous NMR structure of the HBD-DNA^CT^-2ATP complex (PDB: 6GVT), which showed that, even before T2 and ATP base pairing, the ATP triphosphate is tightly coordinated at the HBD NTP^S^ binding site and forms hydrogen-bond interactions between G3 and HBD. These interactions position G3 close to ATP and favor stacking with both T2 and ATP after formation of the two base pairs. By contrast, the available MsCAPP structure lacks the ancillary HBD. As a result, the triphosphate of the first NTP appears less constrained, and the corresponding template base (A8) stacks primarily with the adjacent DNA base (C7) rather than with the isolated nucleotide triphosphate.

The transition from single- to double-base-pair binding did not substantially alter the overall protein architecture; the two primase models remain similar overall, with a backbone RMSD of 1.2 Å for the protein (Supplementary Fig. 9a). The relative orientations of the HBD and the catalytic domains were also largely preserved (Supplementary Fig. 9b). The most prominent difference was observed in the interdomain linker region (Supplementary Fig. 9c). In the 2bp_closed state, the C-terminal portion of the linker adopts a helical conformation, promoting a more compacted closed conformation of the HBD and catalytic domain. This refolding of the helix turn is possible because the linker no longer interacts with T2 (Fig. 4d). This structural rearrangement is experimentally validated by the chemical shift values of the linker residues V258 and E259, which move toward those of the 0bp_open state, where the helix is formed (Supplementary Fig. 9d, e).

In the I-site, the T2-ATP base pair adopts a binding mode like the C1-dGTP base pair in the 1bp_closed state, but with greater stability due to stacking interactions with the adjacent C1-dGTP base pair in the E-site (Fig. 4e). In the E-site, the dGTP triphosphate is tightly coordinated by residues K65, T139, H141, H145, K195, and Y207, along with magnesium ion MgB, which stabilizes interactions with active-site residues D111 and E113 (Fig. 4f). Additionally, the 3′-OH of dGTP forms a hydrogen bond with L180, while its 4′-O group interacts with Q173, further stabilizing the C1-dGTP base pair in the E-site. In this 2bp_closed state, three magnesium ions facilitate the nucleophilic attack of ATP’s 3′-OH group on the α-phosphate of dGTP (Fig. 4g). Magnesium MgA coordinates ATP’s triphosphate group, enabling co-binding by the HBD and catalytic domain (Fig. 4e). Magnesium MgB ensures tight binding of dGTP in the E-site, while magnesium MgC aligns the 3′-OH group of ATP and the α-phosphate of dGTP to be within 4.1 Å, promoting catalytic activity (Fig. 4f, g).

The three-magnesium-ion configuration is supported by our NMR data showing that the triphosphate group of the dinucleotide product remains bound at the NTP^S^ site after dinucleotide formation (Supplementary Fig. 7c), indicating that the ATP triphosphate occupies this site in the 2bp_closed state. Because NTP binding at this site requires Mg^2+^ coordination (Supplementary Fig. 9f), these data support the presence of a magnesium ion at the NTP^S^ site. Consistently, the MsCAPP primase structure (PDB: 7QAZ) contains two catalytic metal ions in the active site and the third metal ion coordinating the triphosphate of the initiating NTP.

Almost all key residues in the catalytic domain that are modeled to interact with DNA and NTPs are highly conserved among archaeal primases (Supplementary Fig. 10). Many of these residues have previously been shown by mutagenesis, using conventional gel-based primase assays, to be essential for DNA-binding and enzyme activity^20,47^, providing an independent biochemical framework for interpreting our NMR-based priming assays. To further validate our structural model, we mutated additional residues predicted to stabilize the substrates in the catalytic domain (Supplementary Figs. 6d, 11a–c). These included Q73, K157, and N176, which interact with the DNA phosphate backbone; W72, N159, and Q173, which contact the ribose moieties of the two base pairs; K167, which contacts the ATP triphosphate; and T139, H141, H145, K195, and Y207, which coordinate the dGTP triphosphate. Primase activity was monitored by time-resolved 1D ^31^P NMR spectroscopy using wild-type or mutant pRN1 primase. Substitutions at these positions caused marked reductions in dinucleotide formation, consistent with a model in which these residues stabilize the DNA template, the initiating nucleotides, and their triphosphates in a catalytically competent arrangement. Together, these findings are consistent with a model in which precise alignment of the HBD and catalytic domain is required for efficient catalysis.

### Mechanism of the C1-dGTP base pair transfer from the I to E site

Our NMR data reveal that the C1-dGTP base pair initially forms in the HBD before being transiently recognized in the I-site and subsequently transferred to the E-site. To explore the mechanism of this transfer, we compared the interactions of the C1-dGTP base pair in the 1bp_closed and 2bp_closed states. In the 1bp_closed state, residues K66 and K195 interact with C1 and dGTP, respectively, within the I-site. Notably, both residues retain these interactions in the E-site of the 2bp_closed state (Fig. 4h). This suggests that K66 and K195 act as molecular anchors facilitating the transfer of the base pair. Mutagenesis of K195 to alanine (K195A) resulted in a 15-fold reduction in primase activity and impaired the transfer of the C1-dGTP base pair, as indicated by incomplete chemical shift transitions of V258 after 40 min of reaction (Fig. 4i and Supplementary Fig. 11c).

Energetically, the 1bp_closed to 2bp_closed transition can be explained as well because the 2bp_closed state is stabilized by the two hydrogen bonds formed by the T2-ATP base-pairing, the stacking of the two base-pairs and the numerous contacts to the dGTP in the catalytic domain at the E-site and finally the refolding of the Linker in a helical turn. This compensates for the removal of the ATP from the NTP^W^ site and the loss of the hydrophobic interactions between T2 and the Linker.

### Proofreading and DNA template selection by primase

Our structure of HBD-DNA^CT^-ATP-dGTP revealed the importance of the second nucleotide (dNTP, which is dGTP in our structure) for initial base-pair formation. We now explored whether this single base-pair formation may be critical for primase fidelity or proofreading. We therefore systematically tested the base-pairing capacity of all four nucleotides at the first template position (N1) of the DNA^N1N2^ sequence.

**Fig. 5:**
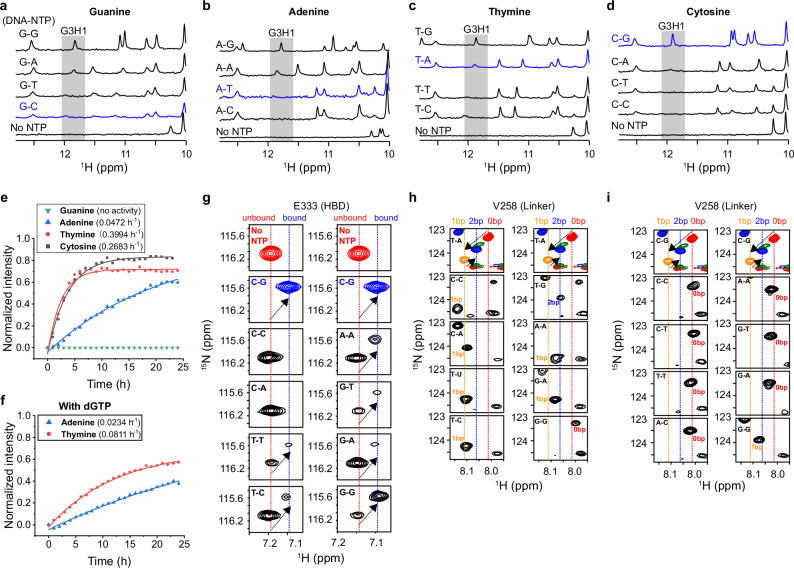
Proofreading and DNA template selection by primase. **a**–**d** Single base-pair formation by the isolated HBD with guanine (**a**), adenine (**b**), thymine (**c**) or cytosine (**d**) at the N1 position of the DNA^N1N2^ template. Base pair stability was assessed using the G3 imino proton. Watson-Crick base pairs are highlighted in blue. **e** Time-dependent normalized intensities of dinucleotide formation by primase with guanine (green), adenine (blue), thymine (red), or cytosine (black) at N1 using their cognate NTPs. **f** Dinucleotide formation by the primase with adenine (blue) or thymine (red) at N1 in the presence of cognate NTPs and dGTP. **g** Single base-pair formation by the HBD in the context of the primase. Base pair stability was monitored via chemical shift changes of residue E333, which is located near the two NTP binding sites of the HBD (Supplementary Fig. 12a, b). **h** Formation of two base pairs by the primase with a mismatched pair at the I-site and a Watson-Crick pair at the E-site, monitored by V258 shifts. T-G, G-A, and A-A mismatches were validated by activity assays (Supplementary Fig. 12c). **i** Two-base-pair formation with mismatched pairs at the E-site and Watson-Crick pairs at the I-site. G-G and G-T mismatches were confirmed by activity assays (Supplementary Fig. 12 d).

Surprisingly, only pyrimidines (thymine and cytosine) at the N1 position formed stable Watson-Crick base pairs, while purines (guanine and adenine) failed to do so (Fig. 5a–d), resulting in markedly reduced or abolished primase activity (Fig. 5e). Further analysis showed that cytosine was the only N1 nucleotide that did not form stable mismatched base pairs, whereas thymine, adenine, and guanine readily mispaired with incoming purine dNTPs. These findings suggest that the preference of a pyrimidine at N1 arises from the favorable stacking of the G3 on the cognate purine dNTP of the N1 template. When N1 is not a cytosine, the HBD fails to distinguish cognate from non-cognate dNTPs, allowing stable mismatches to form and significantly slowing down the reaction (Fig. 5f). This limited specificity is partially corrected by the catalytic domain. In the primase, none of the templates with mismatched base pairs can be fully bound (Fig. 5g and Supplementary Fig. 12a, b). Moreover, these mismatches cannot bind at either the I-site (Fig. 5h and Supplementary Fig. 12c) or the E-site (Fig. 5i and Supplementary Fig. 12d), except for a weakly bound T-G wobble pair at the I-site.

Together, these results indicate that the HBD and catalytic domain act in concert to monitor and proofread incoming dNTPs, thereby contributing to the fidelity of primer initiation. Although this proofreading is imperfect, it likely explains the evolutionary preference for cytosine at position N1 observed in many primases, including human, CAPP and *E. coli* primases, as cytosine does not permit the formation of mismatched base pairs^9,11^. Our findings in pRN1 support this model, suggesting that the ancillary domain enhances fidelity not by actively rejecting mismatches, but by preferentially binding cytosine at N1, thus indirectly preventing incorrect base pairing.

A similar mechanism applies at the second template position (N2). When two base pairs are formed and N1 is transferred from the I-site to the E-site, G3 stacks with the N2 nucleotide located at the I-site (Fig. 4b, c). Cytosine is again preferred due to its ability to efficiently form a canonical Watson-Crick pair with GTP. Thymine is also tolerated at the N2 position because the T-G wobble pair it forms with GTP can be accommodated at the I-site (Fig. 5h and Supplementary Fig. 12c). Importantly, this base pair does not need to be transferred to the E-site, where such mismatches would be rejected and catalysis impaired (Fig. 5i and Supplementary Fig. 12d). These structural constraints explain the evolutionary preference for pyrimidines (cytosine and thymine) over purines at the N2 position. These observations are in line with prior biochemical studies showing a preference for pyrimidines at both the N1 and N2 positions, as well as a general preference for base pairing at these sites^23^.

## Discussion

Until recently, the molecular mechanism underlying dinucleotide formation—the essential initial step in primer synthesis by DNA primase—remained poorly understood structurally. The prevailing model proposes that the catalytic domain initially forms a low-affinity complex with ssDNA and scans along the template to locate potential priming sites. A purine NTP subsequently binds at the E-site, followed by the binding of a second purine NTP at the I-site^48^. This model is supported by in vitro evidence showing that the isolated catalytic domain can catalyze de novo primer synthesis in the presence of high manganese concentrations, although with markedly reduced efficiency compared to the enzyme operating under physiological conditions (magnesium) and in the presence of ancillary domains, such as the pRN1 HBD or human PriL-CTD, provided in *cis* or *trans*^24,49,50^. By contrast, an alternative model for human primase initiation proposes that the ancillary domain provides the dominant substrate-binding platform, whereas the catalytic domain must align with it in a catalytically competent configuration to enable dinucleotide formation^44^.

Here, we present mechanistic and structural insights into the stepwise assembly of the first two NTPs during dinucleotide formation, which explain the critical role of the ancillary domain under physiological conditions.

### Stepwise mechanism of dinucleotide formation by DNA primase

**Fig. 6:**
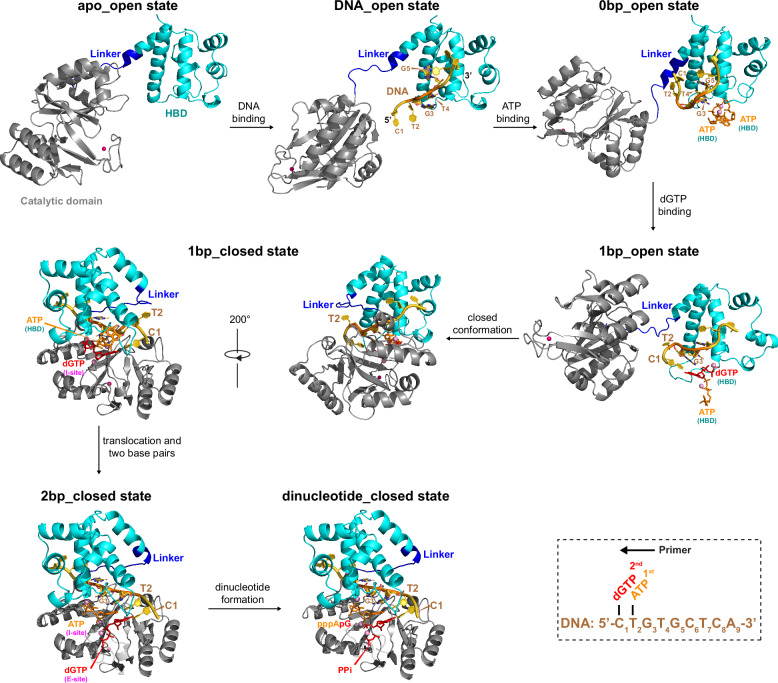
Stepwise mechanism of dinucleotide formation by DNA primase. NMR-derived structural models illustrating the sequential assembly of the DNA template and NTPs during dinucleotide formation by pRN1 DNA primase. The apo_open, DNA_open, 0bp_open and 1 bp_open states are based on the HBD structures from PDB entries 6GT7 (apo HBD), 6GVU (HBD-DNA^CT^), 6GVT (HBD-DNA^CT^-2ATP), and the current structure of the HBD-DNA^CT^-ATP-dGTP complex, respectively. In all four states, the catalytic domain is modeled using the crystal structure of the pRN1 primase (PDB: 3M1M). The 1bp_closed and 2 bp_closed states are from current NMR structural models of the primase-DNA^CT^-1bp and primase-DNA^CT^-2bp complexes, respectively. The dinucleotide_closed state is modeled from the primase-DNA^CT^-2bp complex (this work) in combination with the product complex of DNA polymerase iota bound to pyrophosphate (PDB: 9DQU).

Our structural and spectroscopic data support a sequential model for how pRN1 primase assembles the DNA template and the first two NTPs during dinucleotide formation (Fig. 6 and Supplementary Movie 2). Initially, the HBD binds ssDNA nonspecifically and scans along the template until it encounters the GTG motif, a key sequence element required for priming. Specific recognition of this motif occurs upon the binding of two rNTP molecules (rNTPs being 10 to 100 times more abundant than dNTPs in total cellular concentrations)^51^. Upon GTG recognition, dGTP is recruited and base pairs with the first nucleotide (C1) of the DNA^CT^ template, displacing one of the previously bound rNTP molecules (the affinity of HBD for the template in the presence of the cognate dGTP being five-fold higher than a non-cognate rNTP; Supplementary Fig. 3). The remaining rNTP remains nonspecifically associated with the HBD without forming a base-pair. We initially expected the two cognate NTPs to form two base-pairs within the HBD, and the observed one base-pair intermediate was therefore a major surprise. However, it now rationalizes previous literature that already observed that the first nucleotide to bind primases is the second NTP (the elongating NTP) and not the first one (the initiating NTP)^9,52^.

We next found that the single base pair formed within the HBD is transiently recognized by the catalytic domain, resulting in the DNA template and the two NTPs being sandwiched between the HBD and the catalytic domain. Again, quite surprisingly, the base-pair containing the second NTP is bound to the I-site and not to the expected E-site. This initial binding to the I-site can be explained by the stacking of dGTP with G3 of the template, which was already seen in the 1bp_HBD state. Even more surprising is that the closure of the two domains is triggered by the fact that T2 is bulged out and interacts with the interdomain linker, unfolding part of the N-terminal helix α7 of the HBD to elongate the linker. In a second step, the base-pair is transferred to the E-site, allowing T2 to now base-pair with the ATP to occupy the I-site. This second conformational change is possible because of favorable enthalpic energy (formation of the second base-pair, refolding of the helix α7, preference for rNTP and dNTP in the I and E-sites, respectively). Crucially, in this pre-catalytic state, the ATP is simultaneously coordinated by the HBD, the DNA template (through pairing with T2, stacking with G3, and hydrogen bonding with G3 and T4), and the catalytic domain, positioning its 3’-OH group for an in-line attack on the α-phosphate of dGTP to facilitate dinucleotide formation.

### Closure explains why dinucleotide formation is the rate-limiting step of primer synthesis

Dinucleotide formation represents the rate-limiting step in primer synthesis by all primases^12^. Using NTP analogues to slow down the reaction, we could identify the different steps required to achieve dinucleotide formation. Among the different intermediates, we clearly see a slow step when the HBD is bound by the template, and the two cognate NTPs and one base-pair is formed (Fig. 3a and Supplementary Fig. 4c). Here, an equilibrium between an open form and the closed form is present, where the open form is more abundant (Supplementary Fig. 5a–d). The enthalpy gained by the binding of the catalytic domain probably does not compensate for the entropic cost required for stabilizing the interdomain linker. Indeed, the interdomain linker must be elongated by unfolding helix 7 and then must adopt a fully extended conformation to facilitate domain closure (Fig. 3h). This closing step is therefore likely to be at the origin of the slow rate observed for dinucleotide formation.

### A precise nucleotide alignment for catalysis

Unlike DNA polymerase, which requires a pre-existing primer-template duplex to initiate synthesis, DNA primase can initiate nucleotide polymerization de novo. For catalysis to proceed, the 3’-OH group of the initiating NTP at the I-site must be precisely aligned for nucleophilic attack on the α-phosphate of the elongating NTP at the E-site. In DNA polymerases, this geometry is achieved by the stable pairing within the preformed primer-template duplex (Supplementary Fig. 13a). Without an ancillary domain such as the HBD of pRN1 primase, DNA polymerases are unable to stabilize the 3’-OH of a free nucleotide at the I-site.

Our NMR data show that the HBD of pRN1 primase facilitates this precise alignment by first preparing the two initial NTPs for base pairing with the template and subsequently handing them over to the catalytic domain. The triphosphate of the first NTP is tightly coordinated through interactions with the HBD, DNA template, and catalytic domain (Fig. 4e). Moreover, refolding of the helix 7 of the HBD promotes a more compact closed conformation (Fig. 4d). All these features ensure that the 3’-OH group of the initiating NTP is tightly and correctly positioned in the I-site, even without a preformed primer (Supplementary Fig. 13b). This geometry is essential for efficient nucleophilic attack and primer synthesis.

Without such stabilization, as in DNA polymerase lacking an ancillary domain, de novo initiation is not possible. However, the stabilization provided by primase is inherently weaker than that provided by a preformed duplex, making the initial two nucleotides in the primer more error-prone^9^. This relatively low fidelity likely underlies why DNA primase synthesizes an RNA primer: the RNA segment can be readily identified and removed by RNase, thereby minimizing the risk of propagating errors into the mature DNA strand^12^.

### Proofreading by DNA primase

Although primases lack a 3’->5’ exonuclease domain and thus cannot remove misincorporated nucleotides enzymatically, our data reveal a distinct proofreading mechanism based on nucleotide competition. We observed that some mismatched base-pairs (MBPs) can form within the HBD (Fig. 5a–c). However, in the primase, MBPs are proofread by the catalytic domain either by preventing closing of the one base-pair complex or by preventing translocation to the E-site and the ensuing second base-pair formation in the I-site (Fig. 5g–i). This layered specificity (first base-pair formation, translocation and then second base-pair formation) ensures that only Watson-Crick base pairs are accommodated sequentially in both the I-site and E-site, allowing some proofreading of the primase at this initiation stage. Yet, probably to avoid wasting energy, evolution selected pyrimidine-rich templates, especially cytosines (most eukaryotic primases preferentially initiate on DNA^CC^ and DNA^CT^ sequences^11^), as these are less prone to form MBPs. In addition, pyrimidines at the N1 and N2 template positions ensure that the incoming nucleotide is a purine, which is favorable for dinucleotide formation. Because the incoming nucleotide triphosphate is flexible and forms limited contacts with the protein, stabilization through stacking interactions is important. Purine nucleotides provide stronger stacking interactions either with neighboring template bases (Fig. 1k) or with protein residues, such as the interaction between the initiating GTP and H303 observed in the p58C structure of human primase^29^. Pairing of template pyrimidines with incoming purine nucleotides therefore promotes favorable stacking interactions that stabilize the nascent base pair and facilitate efficient dinucleotide formation.

### Essential role of the ancillary domain in dinucleotide formation

In most DNA primases, the ancillary domains such as human PriL-CTD, PriX from *Saccharolobus solfataricus* and pRN1 HBD, have been shown to enhance primase activity in vitro^20,34,53^. While earlier models suggested these domains stabilize the initiating NTP in the I-site via coordination of the triphosphate with a third metal ion, our data show that their role is considerably broader^48^. We demonstrated that the pRN1 HBD plays critical roles throughout the dinucleotide formation process. It facilitates rapid primer synthesis by recognizing the template sequence-specifically, and by recruiting the two NTPs (first proofreading step). If the second NTP is cognate, the HBD forms an initial base pair, which triggers interaction with the catalytic domain (second proofreading step). If the second NTP is a dNTP and the first NTP is cognate, then translocation of the first base-pair to the E-site and formation of a second base-pair in the I site can occur (third proofreading step). Here, both domains contribute to proofreading since the template and the cognate nucleotides remain in contact with both domains. Our mismatched base pair experiments show that while the HBD tolerates MBPs, the I-site and E-site exhibit high selectivity for Watson-Crick base pairs, ensuring fidelity. Thus, the HBD promotes speed through rapid template scanning and initial nucleotide assembly, while fidelity is enforced when the HBD coordinates with the catalytic domain. This division of labor enables primase to meet the high demands of replication speed and accuracy within the cellular environment.

Our model further suggests that the ancillary domain continues to function after dinucleotide formation. By remaining associated with the 5’ end of the nascent primer, most likely through the 5’-triphosphate (Supplementary Fig. 7c), the HBD could stabilize the growing duplex while the catalytic domain extends the 3’ end. Such a role would be consistent with broader models for archaeal-eukaryotic primases, in which the ancillary domain could act not only as a processivity factor during early extension but also as a determinant of primer-length control by imposing a geometry-dependent limit as the duplex grows^11,48^. In pRN1, previous biochemical work argues against a simple caliper model based solely on linker length, suggesting instead that termination reflects a more specific structural constraint intrinsic to the primase^21^. Consistent with this view, the ancillary domain of human primase, the C-terminal domain of PriL, has previously been shown to regulate both primer length and handoff to Polα, indicating that ancillary domains can stabilize the 5’-end of the product during elongation and help position the completed primer for release or transfer after termination^17,44–46^.

### Conserved mechanism of dinucleotide formation in DNA primases

DNA primases universally contain at least a catalytic domain and an ancillary domain. Although primases, even within the archaeal-eukaryotic primase superfamily, are highly divergent in sequence across kingdoms^15^, these domains work in concert to ensure precise dinucleotide formation—the first step in primer synthesis.

Extensive evidence from bacterial DnaG-type primases and archaeal-eukaryotic primases highlights the essential role of the ancillary domain in vivo during primer initiation^33,49,54,55^. Ancillary domains consistently exhibit higher DNA-binding affinity than their catalytic counterparts, particularly in the presence of the correct initiating NTPs^54,56–59^. In line with this, our previous NMR structure of the pRN1 HBD bound to a DNA template and two ATP molecules revealed that the HBD—rather than the catalytic domain—engages the DNA–NTP complex^22^.

Moreover, the ancillary domains of primases, such as those from T7 and human, mediate sequence-specific recognition of a trinucleotide motif in the DNA template. This motif includes the two templating bases required for dinucleotide synthesis and a third, non-templating base that defines the initiation register^54,58^. These findings support a model in which the first base pair of the primer–template duplex is formed within the ancillary domain, not the catalytic site. Structural data further corroborate this mechanism. In systems such as bacterial DnaG (e.g., T7 replisome) and human primase (PriS/PriL), ancillary domains bind the template/primer duplex and specifically interact with the primer’s 5′-triphosphate^29,60^. Biochemical studies of calf thymus primase further show that the second NTP binds first, defining the initiation site and strongly influencing where synthesis begins^52^. These observations are consistent with our NMR structure of the pRN1 HBD-DNA^CT^-ATP-dGTP complex. The pRN1 HBD concurrently recognizes the DNA template and the second NTP, primarily through contacts with the NTP’s triphosphate, and facilitates formation of the first base pair. Furthermore, the ability of other ancillary domains to bind the primer’s 5′-triphosphate suggests a conserved function in positioning the 3′-OH of the first cognate NTP correctly at the I-site in the 2 bp_closed state, as demonstrated in our NMR structural model of the pRN1 primase–DNA–2bp complex. Therefore, similar structural features across diverse primase systems point to a conserved mechanism by which ancillary domains orchestrate the preparation and stabilization of both initiating nucleotides^9,10^.

Taken together, our pRN1 results support a broadly shared principle of primer initiation, in which the ancillary domain forms the first productive base pair with the second cognate NTP before this intermediate is handed over to the catalytic domain. In this view, the ancillary domain first engages the DNA template, organizes the initiating nucleotide, and stabilizes the first base-pairing event. After transfer to the catalytic domain and dinucleotide formation, the ancillary domain is proposed to continue stabilizing the 5’-triphosphate of the nascent primer during early extension, while the catalytic domain adds incoming nucleotides to the 3’-end. We suggest that this overall functional logic may be shared by many primases. By contrast, the precise structural details observed here, including GTG recognition and linker-mediated sensing of a flipped template base, are more likely to be system-specific.

## Methods

### Protein expression and purification

Three constructs derived from pRN1 primase (UniProt accession number Q54324)—the helix bundle domain (HBD, residues 256–370), catalytic domain (residues 40–248), and primase (residues 40–370)—were expressed and purified as previously described^22^. Constructs were cloned into the pET28c vector for expression in *E. coli* BL21 (DE3) CodonPlus. The HBD and catalytic domain constructs contained an N-terminal His6 tag with a TEV protease cleavage site to facilitate purification and optional tag removal, while the primase had a C-terminal His6 tag without a cleavage site.

Cells were grown at 37 °C until an optical density (OD600) of 0.6–0.7 was reached. Protein expression was then induced by adding 1 mM IPTG, followed by overnight incubation at 30 °C. Proteins were expressed in LB medium (unlabeled proteins) or M9 minimal medium supplemented with 1 g/L ^15^NH₄Cl and either 4 g/L glucose or 2 g/L ^13^C-glucose for isotopic labeling. Cells were harvested and lysed using a microfluidizer (three cycles at 15,000 psi) in Buffer A (50 mM Tris, 1 M NaCl, 10 mM imidazole, pH 8.0). Clarified lysate (30,000×*g*, 4 °C, 40 min) was loaded onto a 5 mL Ni²⁺-charged HiTrap IMAC FF column equilibrated in Buffer A. Proteins were eluted using an imidazole gradient, dialyzed overnight at 4 °C in Buffer B (50 mM Tris, 1 M NaCl, pH 8.0) with TEV protease, and re-applied to the IMAC column in Buffer B. Cleaved proteins in the flow-through were further purified by size-exclusion chromatography using Buffer C (50 mM MOPS, 50 mM NaCl, pH 7.0) or Buffer D (25 mM sodium phosphate, 50 mM NaCl, pH 7.0). Protein purity was monitored by SDS-PAGE. Mutant proteins were purified using identical protocols.

### Mutagenesis

Site-directed mutagenesis was performed using the QuikChange method, employing mutagenic primers complementary to plasmid regions flanking the targeted mutation. PCR with *Pfu* polymerase generated mutant plasmids, which were treated with *DpnI* enzyme to digest the methylated parental DNA, leaving only the newly synthesized mutated plasmid intact. Mutated plasmids were transformed into competent *E. coli* cells, and mutations were verified by DNA sequencing.

### Chemical shift perturbations (CSP)

Solution-state NMR experiments were conducted at pH 7.0 using Bruker AV NEO 500 MHz, 600 MHz, 700 MHz, and 1.2 GHz, as well as Avance III HD 900 MHz spectrometers. Experiments were performed at 298 K (HBD samples) or 313 K (the primase samples), unless stated otherwise. Data were processed with Topspin 4.0 (Bruker) and analyzed using NMRFAM-SPARKY.

The CSP values were calculated using the formula: ∆δ = [(δH)^2^ + (δN/6.51)^2^]^1/2^. For visualization in CSP plots, residues that were unassigned in both states (before and after NTP addition) were assigned a negative placeholder value, and residues assigned only in the state before NTP addition were assigned a larger negative value to distinguish missing peaks. For the HBD constructs, these values were set to −0.2 ppm and −0.5 ppm, respectively. For the catalytic domain constructs, smaller placeholder values (−0.02 ppm and −0.05 ppm, respectively) were used to accommodate the different CSP scale in these datasets. The secondary structure elements are depicted as follows: α, α-helices (cylinders); η, 3_10_-helices (cylinders); β, β-strands (arrows).

### NMR backbone and side-chain assignments

For structure determination of the HBD-DNA^CT^-ATP-dGTP complex, an initial sample containing 0.7 mM ^13^C,^15^N-labeled HBD with 0.77 mM DNA^CT^ template, 2.8 mM ATP, and 2.8 mM dGTP was prepared. However, due to ATP and dGTP auto-hydrolysis, the complex was unstable. Replacing ATP and dGTP with non-hydrolysable analogs (ATP^C^ and dGTP^C^) stabilized the complex for over a month. Thus, the final complex used contained 0.8 mM ^13^C,^15^N-labeled HBD, 0.8 mM DNA^CT^, 1.6 mM each of ATP^C^ and dGTP^C^. Backbone resonances were assigned via standard triple-resonance experiments (HNCA, HN(CO)CA, HNCO, HN(CA)CO, HNCACB, and CBCA(CO)NH). While side-chain assignments were achieved using (H)C(CCO)NH, H(CCCO)NH, (H)CCH TOCSY, and HC(C)H TOCSY experiments. Assignments for DNA^CT^, ATP^C^, and dGTP^C^ involved 2D F1-filtered, F2-filtered ^1^H-^1^H NOESY and standard homonuclear NOESY/TOCSY experiments. Additionally, ^13^C-natural and ^15^N-natural abundance HSQCs were recorded with an unlabeled sample containing 4 mM protein, 4.4 mM DNA^CT^, 4 mM ATP^C^, and 4 mM dGTP^C^.

Assignments for the primase were initially guided by those of the individual domains and supported by 3D BEST-TROSY HNCA, HN(CO)CA, HNCACB, HN(CO)CACB spectra, using 1 mM uniformly ^13^C, ^15^N-labeled protein. For the primase complexes bound to the DNA template and NTPs, assignments were obtained through titration experiments monitored by 2D ^1^H-^15^N HSQC or BEST-TROSY.

### Solid-state NMR experiments

Solid-state NMR samples were prepared by sedimentation using home-built rotor filling tools^61–64^. All solid-state NMR experiments were performed at Bruker Avance III HD or Bruker Avance Neo spectrometers. The hPH experiment was acquired at a static magnetic field strength of 28.2 T using a 0.7 mm triple-resonance Bruker Biospin probe and a MAS frequency of 100 kHz. The spectra were recorded by using a MISSISSIPPI water suppression scheme. The NHHP and CHHP experiments were acquired at a static magnetic field strength of 11.7 T using a 3.2 mm triple-resonance Bruker Biospin probe and a MAS frequency of 17 kHz. An overview of the experimental parameters for all NMR spectra is given in Supplementary Table 2.

### NMR structural restraints detection

For structure determination of the HBD-DNA^CT^-ATP-dGTP complex, intramolecular restraints for HBD were derived from 3D ^15^N- and ^13^C-resolved ^1^H-^1^H NOESY spectra in either 90% H_2_O/10% D_2_O or 100% D_2_O. Intermolecular restraints were determined by comparing 2D F1-filtered, F2-filtered ^1^H-^1^H NOESY and F2-filtered-only ^1^H-^1^H NOESY. Additionally, 3D ^13^C-resolved F1-filtered, F2-edited ^1^H-^1^H NOESY spectra were used to identify intermolecular NOEs, and 2D ^1^H-^1^H NOESY in 90% H_2_O/10% D_2_O at 283 K captured interactions between the HBD and the imino and amino groups of the GTG motif.

TALOS+ provided dihedral angle constraints for the protein. Intramolecular hydrogen bonds were identified from ^1^H-^15^N hydrogen-deuterium exchange data and the preliminary structure, supported by secondary structure propensities inferred from CA and CB chemical shifts. An additional hydrogen bond between dGTP O3A and K346 was included based on NMR evidence.

Sugar puckers of nucleic acids were identified from 2D ^1^H-^1^H TOCSY and confirmed by intra-nucleotide H6/H8-H2’ versus H6/H8-H3’ NOEs. *Syn* or *anti* glycosidic conformations were inferred from NOE patterns of H6 or H8 resonances. Notably, T2 adopts a *syn* glycosidic conformation and a C2’-endo sugar pucker. The prominent H6-H1’ NOE indicates a short interproton distance consistent with a *syn* glycosidic conformation (Supplementary Fig. 14a). In addition, the strong H1’-H2’ cross-peak observed in the TOCSY spectrum indicates a large ^3^J_H1’H2’_ coupling constant, consistent with a C2’-endo sugar pucker (Supplementary Fig. 14b).

Additional triphosphate binding interactions were characterized by solid-state NMR (NHHP, CHHP, and hPH). A complex containing the primase H145A mutant, DNA^CT^ template, ATP^N^, and dGTP^C^ was prepared. For NHHP and CHHP solid-state NMR, a ^13^C, ^15^N-labeled primase H145A mutant was used, while for hPH solid-state NMR, a ^2^H,^15^N-labeled primase H145A mutant was prepared. The solid-state hPH NMR spectra have been recorded at 28.2 T using an MAS frequency of 100 kHz in a Bruker triple-resonance 0.7 mm probe. The sample temperature was set to 309 K using the water lines as an internal reference^64^. The CP contact times have been set to 3.0 ms and a repetition time of 1.2 s was used. Acquisition times were set to 2.6 ms (F1) and 73.7 ms (F2). Solid-state NMR data provided distance restraints of triphosphates with HBD residues. Solution-state NMR revealed six hydrogen-bond restraints between the HBD and triphosphates, derived from significantly downfield-shifted backbone amide groups upon two NTPs binding, consistent with the previous HBD-DNA^CT^-2ATP NMR structure.

### Structure calculation of HBD-DNA^CT^-ATP-dGTP

Preliminary structures of the HBD in the HBD-DNA^CT^-ATP-dGTP complex were generated using UNIO-ATNOS-CANDID software with automated peak-picking and NOE assignments from 3D ^15^N- and ^13^C-NOESY spectra of bound HBD. Each step of the seven iterations involved calculating 100 independent structures. Manually assigned intramolecular and intermolecular NOEs, hydrogen bonds, and dihedral angle restraints were then integrated into CYANA’s NOEASSIGN module for structural calculation. The 50 lowest-energy structures were refined using AMBER20 SANDER in Cartesian space, with final analysis by AMBER20 scripts and PROCHECK.

### Isothermal titration calorimetry (ITC)

ITC experiments were performed to assess HBD binding to the DNA^CT^ template in the presence of either ATP or dGTP. For the ATP condition, HBD at 250.52 µM (syringe) was titrated into 18.40 µM DNA^CT^ in the presence of 1 mM ATP. For the dGTP condition, HBD at 120.12 µM was titrated into 11.65 µM DNA^CT^ with 1 mM dGTP. All titrations were conducted at 298 K in a buffer containing 25 mM Na₂HPO₄/NaH₂PO₄, 50 mM NaCl, and 10 mM MgCl₂ (pH 7.0), and each experiment was performed in duplicate.

Each experiment, including controls, consisted of 19 injections of 2 µL, spaced 150 s apart, using a MicroCal PEAQ-ITC instrument. The cell volume was 300 µL and the syringe volume was 70 µL. The stirring speed was set to 750 RPM and the reference power to 10 µcal/s.

Raw thermograms were processed with MicroCal PEAQ-ITC Analysis software. The integrated heat changes from each injection were plotted against the molar ratio of DNA to protein, and the binding isotherms were fitted to a one-site binding model to determine the equilibrium dissociation constant (Kd).

### Paramagnetic measurement with Mn^2+^

An NMR sample containing 0.1 mM ^15^N-labeled primase, 0.11 mM DNA^CT^, 0.1 mM dGTP^C^ was first prepared in 25 mM sodium phosphate, 50 mM NaCl, 95% H_2_O/5% D_2_O at pH 7.0. Small volumes of a 5 mM MnCl_2_ solution were progressively added to this NMR sample. [MnCl_2_]/[protein] molar ratios of 0.33, 0.66, and 1 were used to monitor the paramagnetic effect using 2D ^1^H-^15^N HSQC. The control diamagnetic experiment was performed using similar methods. Control experiments with MgCl_2_ were similarly performed, although Mg^2+^ required higher concentrations to achieve saturation due to a weaker binding affinity compared to Mn^2+^. [MgCl_2_]/[protein] molar ratios of 0.33, 0.66, 1, 2, and 3 were added to saturate the protein.

### Primase activity assays

Primase activity was monitored using time-resolved 1D ^31^P NMR spectroscopy. NMR samples were prepared by mixing 20 μM pRN1 primase, 22 μM DNA template, 1 mM rNTP, and 1 mM dNTP in buffer containing 50 mM MOPS (pH 7.0), 50 mM NaCl, 1 mM DSS, and 10% D_2_O. The reaction mixture was then loaded into a 3 mm NMR tube. Initial NMR spectra were acquired at 298 K in the absence of Mg^2+^ to establish reference intensities. Signal intensity corresponding to free DNA at approximately −1 ppm was integrated and defined as *I*_0,DNA_, while Pγ signal from free NTPs around −7.5 ppm was integrated as *I*_0,Pγ_ using Bruker Topspin Dynamic Center software. To initiate the primase reaction, MgCl_2_ was added to a final concentration of 10 mM. Subsequently, 1D ^31^P NMR spectra were acquired at 1-h intervals for 19–24 h. Spectra were collected on a Bruker Avance NEO 500 MHz spectrometer equipped with a CP-QCI ^1^H/^19^F-^13^C/^15^N/^31^P-^2^H Z-gradient probe. Each spectrum consisted of 2048 complex data points, with a spectral width of 40.5 ppm. A total of 3072 scans were acquired per spectrum, corresponding to approximately 1 h per acquisition.

Due to signal overlapping, the dinucleotide product and unbound DNA both resonate near −1 ppm^35^. Thus, the integrated intensity at this position, denoted as *I*_DNA+dinuc_(t), represents the combined signal from both free DNA and newly synthesized dinucleotide. The normalized dinucleotide signal at each time point was calculated using Eq. 1.

1 $${{\rm{Normalized}}}\;{{\rm{intensity}}}\;{{\rm{of}}}\;{{\rm{dinucleotide}}}=\frac{{I}_{{{\rm{DNA}}}+{{\rm{dinuc}}}}\left({{\rm{t}}}\right)-{I}_{0,{{\rm{DNA}}}}}{{I}_{0,{{{\rm{P}}}}_{{{\rm{\gamma }}}}}}$$

It should be noted that primase binds the DNA template less efficiently in the absence of Mg^2+^, resulting in a higher free DNA signal. Upon Mg^2+^ addition, enhanced DNA binding may reduce the free DNA signal. As a result, *I*_DNA+dinuc_(t) may become lower than *I*_0,DNA_, occasionally resulting in negative normalized intensities.

Reaction kinetics of dinucleotide formation were analyzed by fitting the data in OriginPro 2025 to a first-order exponential growth model according to Eq. 2.

2 $$I={I}_{0}+A\left(1-{e}^{-{kt}}\right)$$

where *I* is the normalized intensity of dinucleotide, *I*_0_ is the initial normalized intensity, *A* represents the maximum increase from baseline intensity, *k* is the rate constant (h^−^^1^), and *t* is the reaction time (h).

### Proofreading and DNA template selection by primase

NMR samples were prepared with 0.1 mM protein, 0.11 mM single-stranded DNA template, and 0.4 mM of each NTP in a buffer containing 25 mM sodium phosphate (pH 7.0), 50 mM NaCl, 10 mM MgCl_2_, 1 mM DSS, and 10% D_2_O. For the HBD construct, 1D proton and 2D ^1^H-^15^N HSQC spectra were acquired at 298 K. For the primase, 1D proton and 2D ^1^H-^15^N BEST-TROSY spectra were recorded at 313 K. The sequences of the single-stranded DNA templates and the corresponding NTPs used in each experiment are provided in Supplementary Data 1.

### Reporting summary

Further information on research design is available in the Nature Portfolio Reporting Summary linked to this article.

## Supplementary information

Supplementary information

Description of additional Supplementary files

Supplementary Data 1

Supplementary Movie 1

Supplementary Movie 2

Reporting Summary

Transparent Peer Review file

## Data Availability

The NMR structure of the HBD-DNA^CT^-ATP-dGTP complex, along with the restraints used for its calculation, has been deposited in the Protein Data Bank (PDB, https://www.wwpdb.org/) under accession code 9RTJ. The corresponding chemical shift values are available in the Biological Magnetic Resonance Data Bank (BMRB, https://bmrb.io) under accession number BMRB35007. Structural models of the primase-DNA^CT^-1bp and primase-DNA^CT^-2bp complexes have been deposited in the Protein Data Bank–Integrative/Hybrid Methods (PDB-IHM, https://pdb-ihm.org/) under accession codes 9AAN and 9AAM, respectively. The corresponding chemical shift assignments are available in the BMRB under accession numbers BMRB53262 and BMRB53266, respectively, and the associated restraints are available in BMRbig (https://bmrbig.bmrb.io) under accession numbers bmrbig142 and bmrbig143, respectively.
