# Supplementary material for: Structural and mechanistic insights into primer synthesis initiation by DNA primase
Source: Nat Commun. 2026 Jul 3;17:8299. doi: 10.1038/s41467-026-74862-8 (PMC13469122; doi:10.1038/s41467-026-74862-8)
Supplement: Supplementary file 1 [file 41467_2026_74862_MOESM1_ESM.pdf]

Supplementary information for

Structural and Mechanistic Insights into Primer Synthesis Initiation by DNA Primase

Pengzhi Wu[1†*], Fred F. Damberger[1], Johannes Zehnder[2], Nina Wehr[3,4], Niklas Senning[1], Georg Lipps[5], Thomas Wiegand[3,4], Frédéric H.-T. Allain[1*]

[1]Institute of Biochemistry, Department of Biology, ETH Zürich, Switzerland

[2]Laboratory of Physical Chemistry, ETH Zürich, Switzerland

[3]Institute of Technical and Macromolecular Chemistry, RWTH Aachen University, Germany

[4]Max Planck Institute for Chemical Energy Conversion, Mülheim (Ruhr), Germany

[5]Institute of Chemistry and Bioanalytics, University of Applied Sciences Northwestern Switzerland, Muttenz, Switzerland

†Present address: State Key Laboratory of Chemical Biology, Shanghai Institute of Organic Chemistry, Chinese Academy of Sciences, Shanghai, China

*Corresponding authors: pzwu@sioc.ac.cn; allain@bc.biol.ethz.ch

This Supplementary Information contains:

Supplementary Figures 1-17

Supplementary Tables 1, 2

Supplementary Method

Supplementary Figures

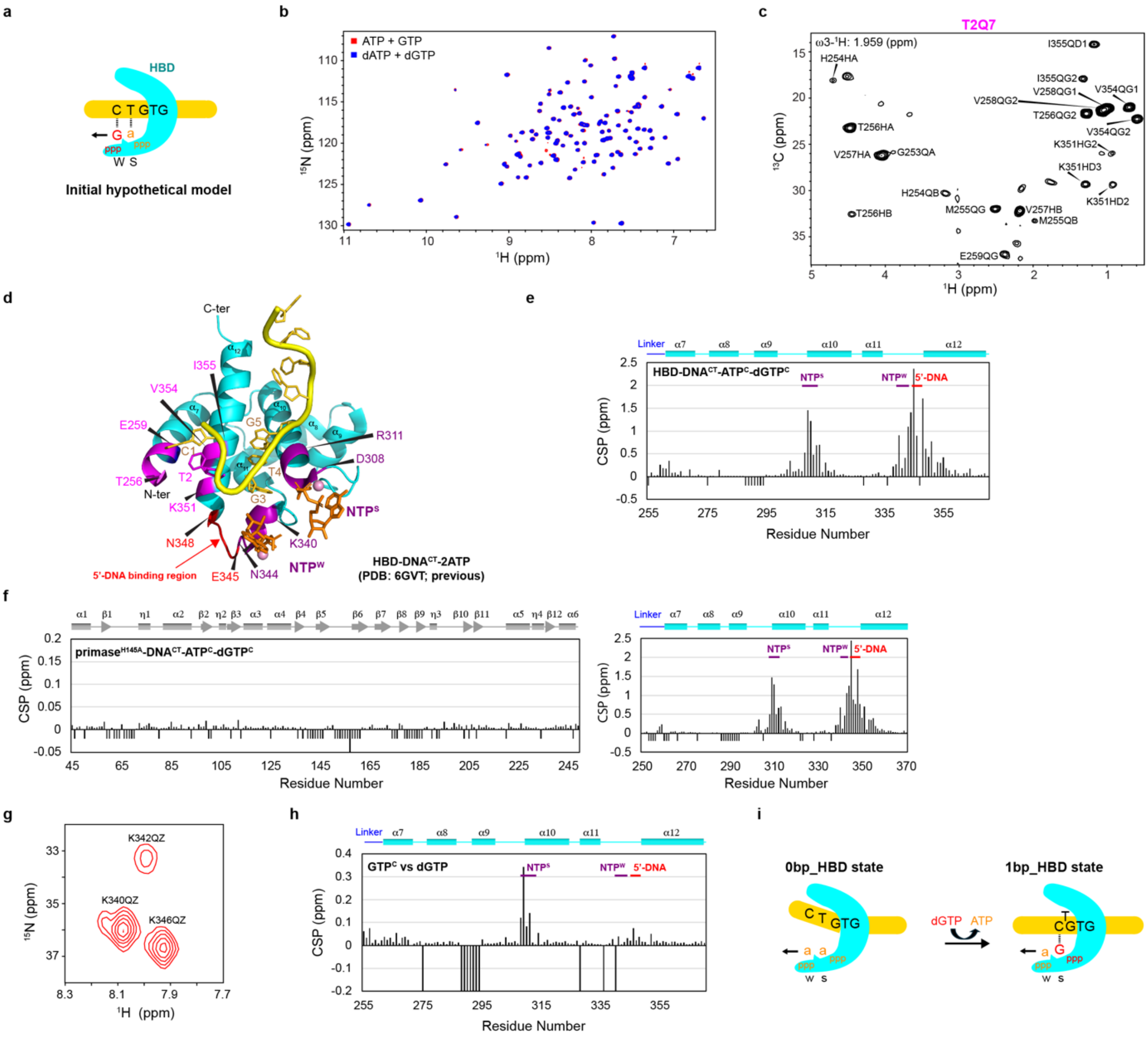

Supplementary Fig. 1 | Binding of Two Cognate NTPs to the HBD Results in a Single Base-pair Formation. a, Initial hypothetical complex in which the HBD binds the first two NTPs, forming two base pairs with the DNA complex. **b**, Overlaid 2D ^{1}H-^{15}N HSQC spectra of HBD-DNA^{CT}-ATP-GTP (red) and HBD-DNA^{CT}-dATP-dGTP (blue) at pH 5.0, 298 K. **c**, Expanded NOE plane showing T2Q7 interactions with the Linker residues in the HBD-DNA^{CT}-ATP^{C}-dGTP^{C} complex from 3D ^{13}C$_{aliphatic}$ Hfiltered-Hedited NOESY spectrum. Only the protein is ^{13}C, ^{15}N-labeled. HA and HB protons are folded into this region. Q denotes a pseudo-atom representing two or three equivalent protons; for example, T2Q7 refers to the methyl group of T2, G253QA the glycine alpha protons and V354QG2 the gamma2 methyl of V354. **d**, NMR structure of HBD-DNA^{CT}-2ATP (PDB: 6GVT). The HBD and DNA (excluding T2) are shown in cyan and gold, respectively. T2 and HBD residues exhibiting NOEs to T2 in the HBD-DNA^{CT}-ATP^{C}-dGTP^{C} complex are highlighted in magenta. ATP molecules are depicted in orange. Residues within the NTP^{S} and NTP^{W} binding sites are colored purple. Residues near the 5’-DNA region are shown in red. **e**, Bar plot of the combined chemical shift perturbations

(CSPs) for the HBD-DNA^{CT} complex upon binding to ATP^{C} and dGTP^{C}. **f**, Bar plot of the CSPs for the primase^{H145A}-DNA^{CT} complex upon binding to ATP^{C} and dGTP^{C}. Left panel: catalytic domain; right panel: Linker and HBD. **g**, 2D ^{1}H-^{15}N HMQC spectrum of the HBD-DNA^{CT}-ATP^{C}-dGTP^{C} complex, showing the assignments of the side-chain amino groups of K340, K342 and K346. **h**, Bar plot comparing CSPs between the HBD-DNA^{CT}-ATP-GTP^{C} and the HBD-DNA^{CT}-ATP-dGTP complexes. **i**, Schematic representation of dGTP addition to the HBD-DNA^{CT}-2ATP complex. Lowercase letters (e.g. *a*) indicate ribonucleotide; uppercase letters (e.g. *G*) denote deoxyribonucleotides. *W* and *S* denote weak and strong nucleotide-binding sites, respectively.

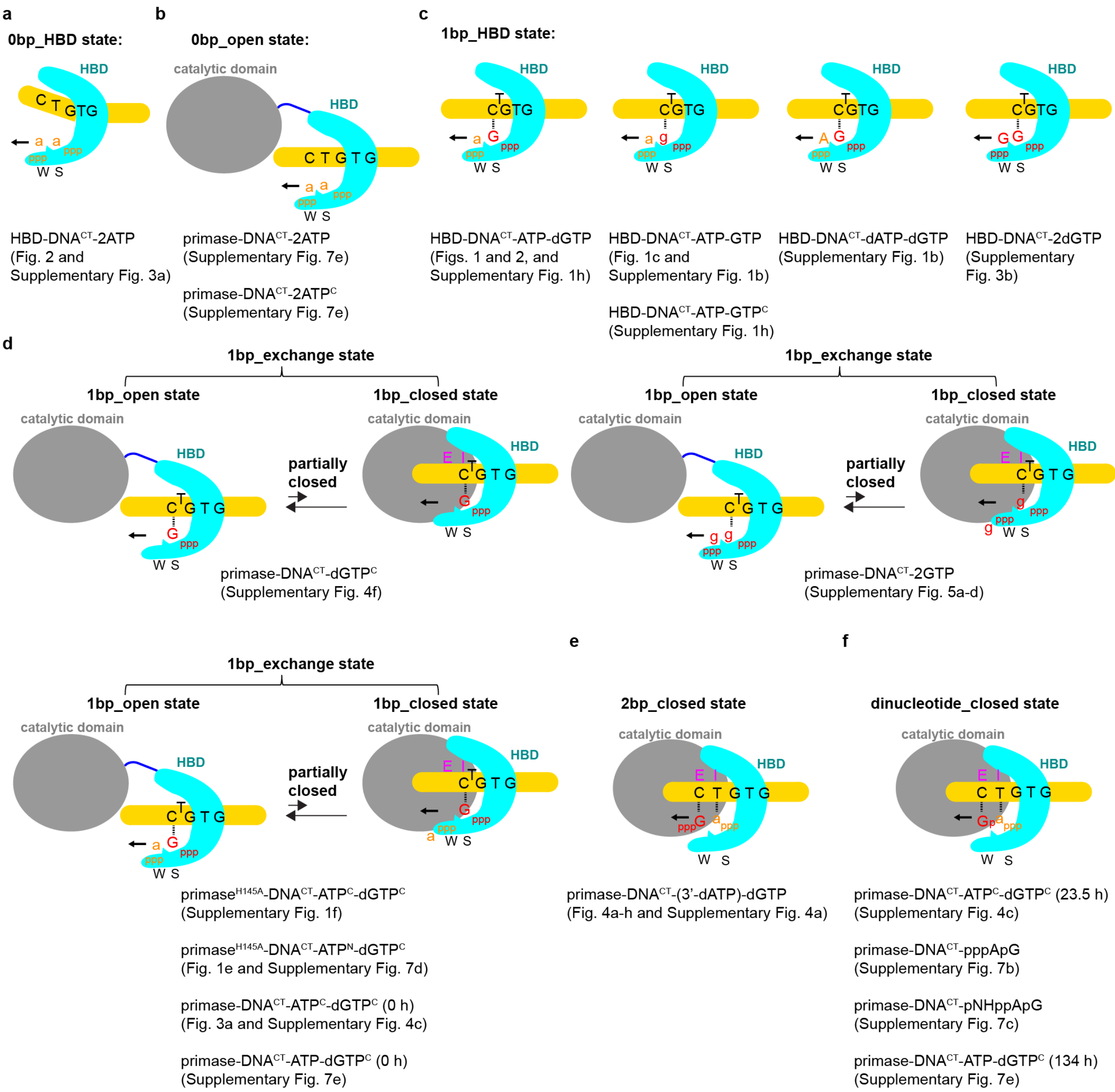

Supplementary Fig. 2 | Summary of pRN1 Primase Complexes and Their Conformational States. *I* and *E* denote the Initiation and Elongation nucleotide binding sites in the catalytic domain. The figure number corresponding to the experiment data for each complex is indicated below its name. **a**, 0bp_HBD: The isolated HBD binds DNA and specifically recognizes the GTG motif in the presence of two ATP molecules, none of which forms any base pair with DNA template. **b**, 0bp_open: In the context of the pRN1 primase, the HBD similary binds and recognizes the GTG motif in the presence of two ATP molecules, none of which forms any base pair with DNA template. **c**, 1bp_HBD: A single base pair is formed between DNA and the second NTP within the free HBD. **d**, 1bp_exchange: Following base-pair formation in the HBD, the single base pair can be transiently accommodated by the I-site of the catalytic domain, resulting in dynamic exchange between open and closed conformations of the primase. **e**, 2bp_colsed: Two base pairs are stably formed in the catalytic domain, while the triphosphate group of the first nucleotide remains coordinated by

the HBD. **f**, dinucleotide_closed: After dinucleotide synthesis, the product is retained in the catalytic domain, with its 5'-triphosphate still engated by the HBD.

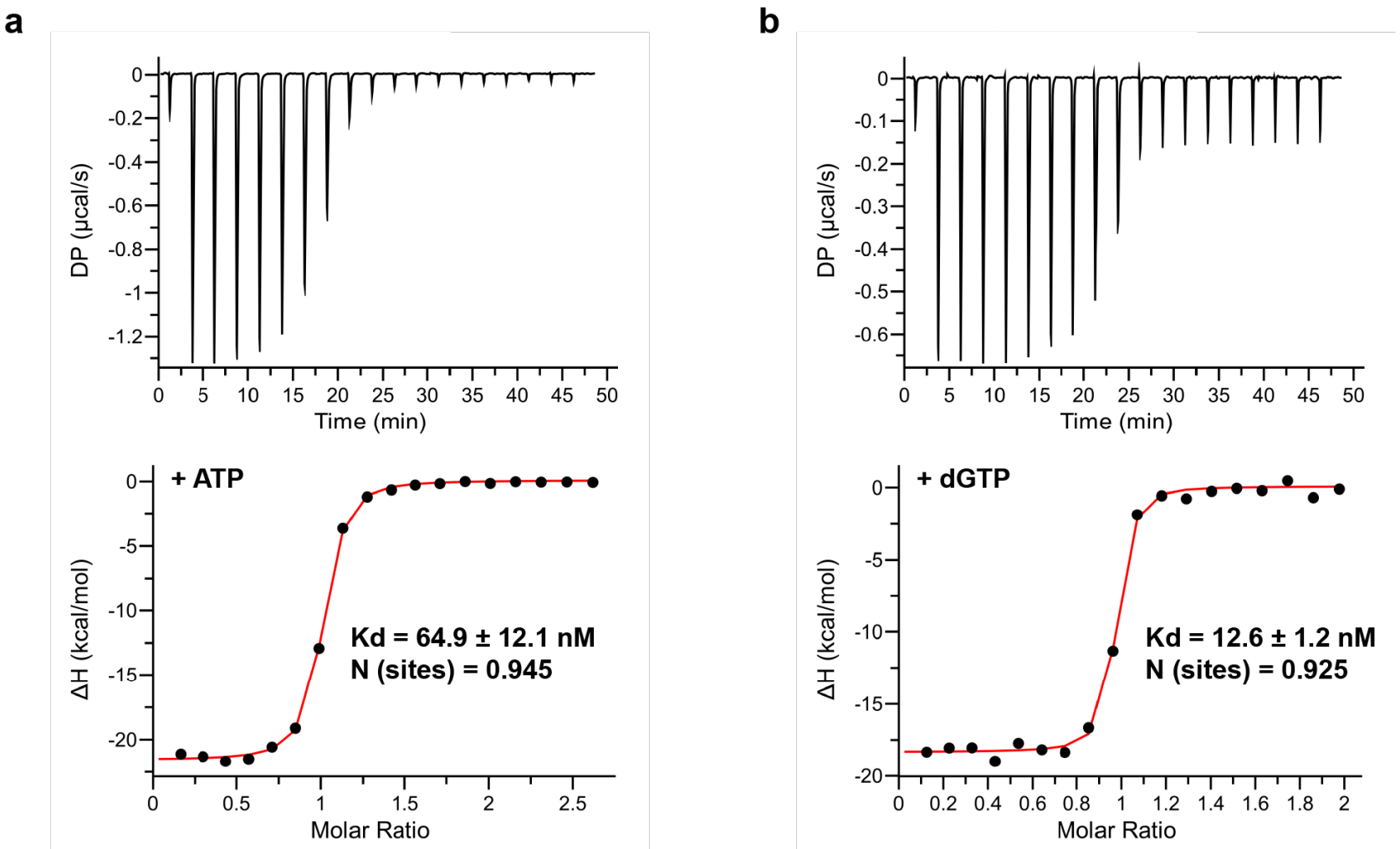

Supplementary Fig. 3 | ITC profile resulting from the injection of the HBD into the template DNA^{CT} in the presence of ATP (a) or dGTP (b).

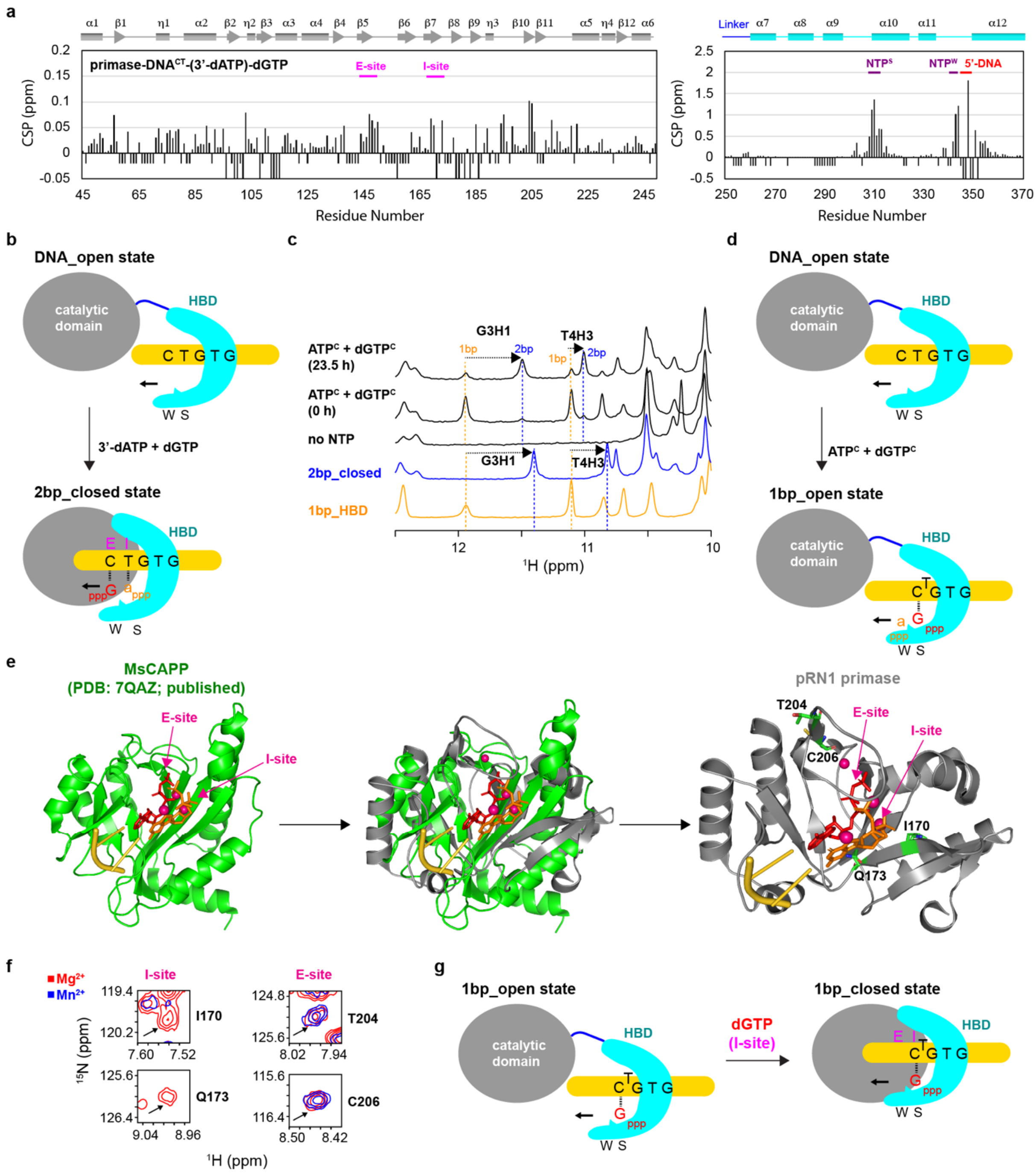

Supplementary Fig. 4 | The HBD-Formed Base Pair Is Initially Recognized by the Catalytic Domain's I-Site. **a**, Bar plot of the CSPs for the primase-DNA^{CT} complex upon binding 3'-dATP and dGTP. Residues within the active site core, such as D111, E113, D171 and H145, were not observed following NTP binding. Residues near the I-site and E-site that exhibit significant CSPs are indicated. **b**, Cartoon representation illustrating the formation of two base pairs after adding 3'-dATP and dGTP into primase-DNA^{CT} complex. The DNA_open state represents the primase bound only to the DNA template without NTPs, in which the two domains adopt an open conformation. **c**, Transition from single base pair to two base pairs. From top to bottom are the complexes: primase-DNA^{CT}-ATP^{C}-dGTP^{C} after 23.5 h reaction, primase-DNA^{CT}-ATP^{C}-dGTP^{C} at the start of the reaction, primase-DNA^{CT},

primase-DNA^{CT}-(3'-ATP)-dGTP (2bp_closed, control), HBD-DNA^{CT}-ATP^{C}-$dGTP^{C}$ (1bp_HBD, control). **d**, Cartoon representation illustrating the formation of one base pair within the HBD just after adding ATP^{C} and $dGTP^{C}$ into primase-DNA^{CT} complex. **e**, Identification of the I-site and E-site in the pRN1 primase. Left panel: crystal structure of the MsCAPP primase complex with two base pairs, showing the I-site and E-site within the active site (PDB: 7QAZ). Middle panel: Structural overlay of the catalytic domains from MsCAPP (green) and pRN1 (gray) primases. Right panel: Model of two base pairs into the active site of pRN1 primases, indicating residues I170 and Q173 adjacent to the I-site, and residues T204 and C206 adjacent to the E-site, shown as sticks. Hot pink spheres represent metal ions: Zn^{2+} in pRN1 and Co^{2+} in MsCAPP. **f**, Overlaid 2D ^{1}H-^{15}N HSQC spectra of primase-DNA^{CT}-$dGTP^{C}$ (1:1.1:1) in the presence of Mg^{2+} (red) or Mn^{2+} (blue). Panels PRE effect in I170 and Q173 from the I-site, and T204 and C206 from the E-site. **g**, Cartoon representation showing initial recognition of the C1-dGTP base pair by the I-site.

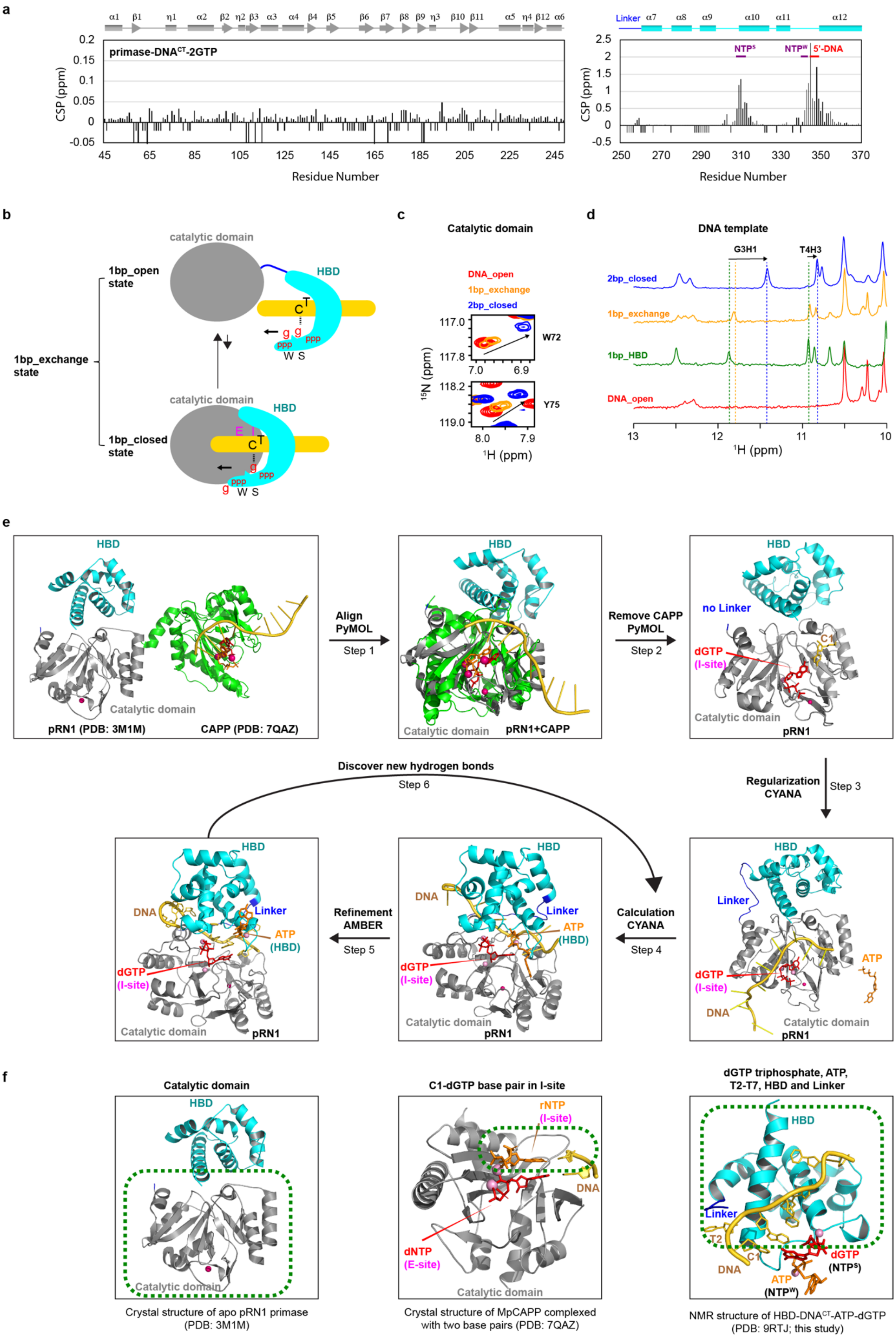

a
primase-DNA^{CT}-2GTP
CSP (ppm)
Residue Number
Linker
NTP^{S}
NTP^{W}
5'-DNA
b
catalytic domain
HBD
1bp_open state
1bp_exchange state
1bp_closed state
c
Catalytic domain
DNA_open
1bp_exchange
2bp_closed
W72
Y75
^{15}N (ppm)
^{1}H (ppm)
d
DNA template
G3H1
T4H3
2bp_closed
1bp_exchange
1bp_HBD
DNA_open
^{1}H (ppm)
e
pRN1 (PDB: 3M1M)
CAPP (PDB: 7QAZ)
Catalytic domain
Align PyMOL
Step 1
pRN1+CAPP
Remove CAPP PyMOL
Step 2
no Linker
dGTP (I-site)
Regularization CYANA
Step 3
Discover new hydrogen bonds
Step 6
Calculation CYANA
Step 4
Refinement AMBER
Step 5
DNA
Linker
ATP (HBD)
pRN1
f
Catalytic domain
C1-dGTP base pair in I-site
dGTP triphosphate, ATP, T2-T7, HBD and Linker
rNTP (I-site)
dNTP (E-site)
dGTP (NTP^{S})
ATP (NTP^{W})
Crystal structure of apo pRN1 primase (PDB: 3M1M)
Crystal structure of MpCAPP complexed with two base pairs (PDB: 7QAZ)
NMR structure of HBD-DNA^{CT}-ATP-dGTP (PDB: 9RTJ; this study)

Supplementary Fig. 5 | Structural Model of the Primase-DNA^{CT}-1bp Complex Based on NMR Data. **a**, Bar plot of the CSPs for the primase-DNA^{CT} complex upon binding to two GTP molecules. **b**, Cartoon representation of the conformational exchange in the primase-DNA^{CT}-2GTP complex (referred to as the 1bp_exchange state) between the 1bp_open (top) and 1bp_closed (bottom) conformations. **c**, Overlaid 2D ^{1}H-^{15}N BEST-TROSY spectra showing chemical shift perturbations within the I-site (W72 and Y75) during the conformational transition from the open state (red, DNA_open) to partially closed intermediate (orange, 1bp_exchange), and finally to the closed state (blue, 2bp_closed). **d**, 1D ^{1}H spectra highlighting imino proton shifts of the DNA template (G3) across the same conformational intermediate states. (**c-d**) NMR spectra representing DNA_open, 1bp_HBD, 1bp_exchange and 2bp_closed conformations were obtained from the complexes: primase-DNA^{CT}, HBD-DNA^{CT}-2dGTP, primase-DNA^{CT}-2GTP and primase-DNA^{CT}-(3'-dATP)-dGTP, respectively. All spectra were acquired at 313 K, pH 7.0. **e**, Structural modeling procedure:

Step 1: Align the active sites of the crystal structure of the apo pRN1 primase (PDB: 3M1M) and MsCAPP complexed with two base pairs (PDB: 7QAZ) using PyMOL.

Step 2: In PyMOL, remove all components from the MsCAPP complex except the GTP-cytosine base pair bound in the I-site. Then, mutate GTP to dGTP using PyMOL's mutagenesis function.

Step 3: Add the unresolved Linker region of the pRN1 primase, ATP, and the remaining DNA sequence using CYANA regularization.

Step 4: Incorporate distance restraints between the HBD, DNA^{CT}, ATP and dGTP from the NMR structure of the HBD-DNA^{CT}-ATP-dGTP complex. Use CYANA to calculate the structure.

Step 5: Refine the final structure using AMBER.

Step 6: Discover new hydrogen bonds after AMBER refinement.

f, Distance restraints for CYANA calculation and AMBER refinement:

Left panel: Distance restraints for the catalytic domain are derived from the crystal structure of apo pRN1 primse (PDB: 3M1M).

Middle panel: Distance restraints for the dGTP-C1 base pair, excluding the dGTP triphosphate, are modeled based on the base pair binding in the I-site of the MsCAPP complex (PDB: 7QAZ).

Right panel: Distance restraints for the dGTP triphosphate, ATP, and nucleotides from T2 to T7 of the DNA^{CT} template, as well as for the HBD and Linker, are taken from our NMR structure of the HBD-DNA^{CT}-ATP-dGTP complex.

The green dashed lines indicate the regions where the distance restraints are derived from the three different structures.

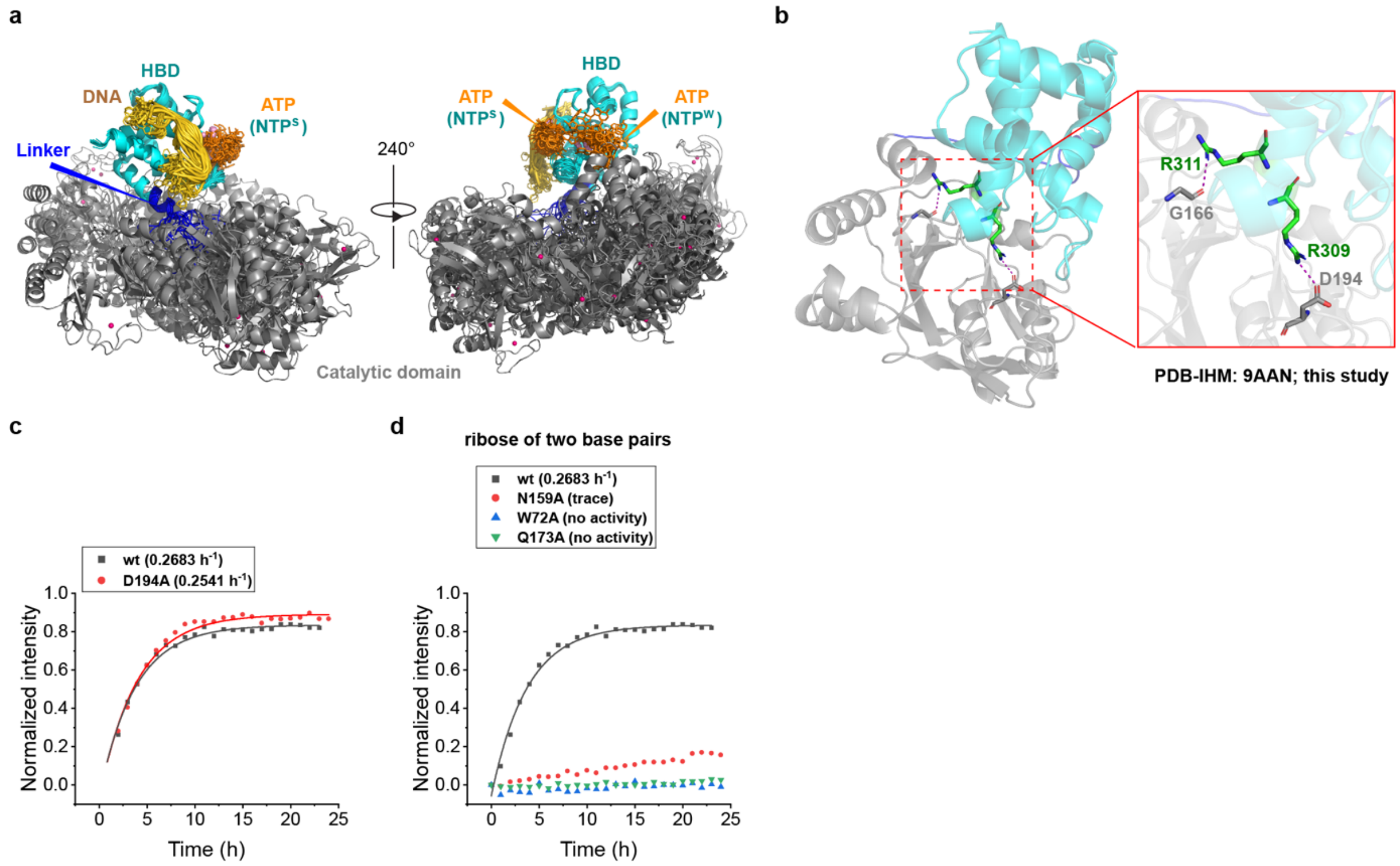

Supplementary Fig. 6 | Transition from Single- to Double-Base-Pair Binding. a, Ensemble structures of the primase-DNA^{CT}-2ATP complex in the 0bp_open state. The 0bp_open state represents a conformation in which the HBD binds to DNA and two ATP molecules, but no base pairing has yet occurred, and the two domains remain in an open conformation. In this state, due to the helical structure formed in the interdomain linker, the linker is no long enough for the catalytic domain to reach the two ATP molecules bound to the HBD. The model was constructed using the same approach as for the primase-DNA^{CT}-1bp complex: the catalytic domain is modeled using the crystal structure of the pRN1 primase (PDB: 3M1M), while the HBD-DNA^{CT}-2ATP region is derived from the NMR structure (PDB: 6GVT). **b**, Two hydrogen bonds between the HBD and catalytic domain (D194 with R309, and G166 with R311) consistently observed in at least half of the primase-DNA^{CT}-1bp complex ensembles. **c**, Time-dependent normalized intensity of dinucleotide formation. Data are shown for the wild-type (black) and D194A mutant (red) primase with the DNA^{CT} template, ATP and dGTP. **d**, Time-dependent normalized intensity of dinucleotide formation. The data show the effects of disrupting hydrogen bonding with the ribose moieties of the two base pairs bound at the I-site (W72 and N159) and E-site (Q173). All reactions were carried out using the DNA^{CT} template and its cognate NTPs, ATP and dGTP.

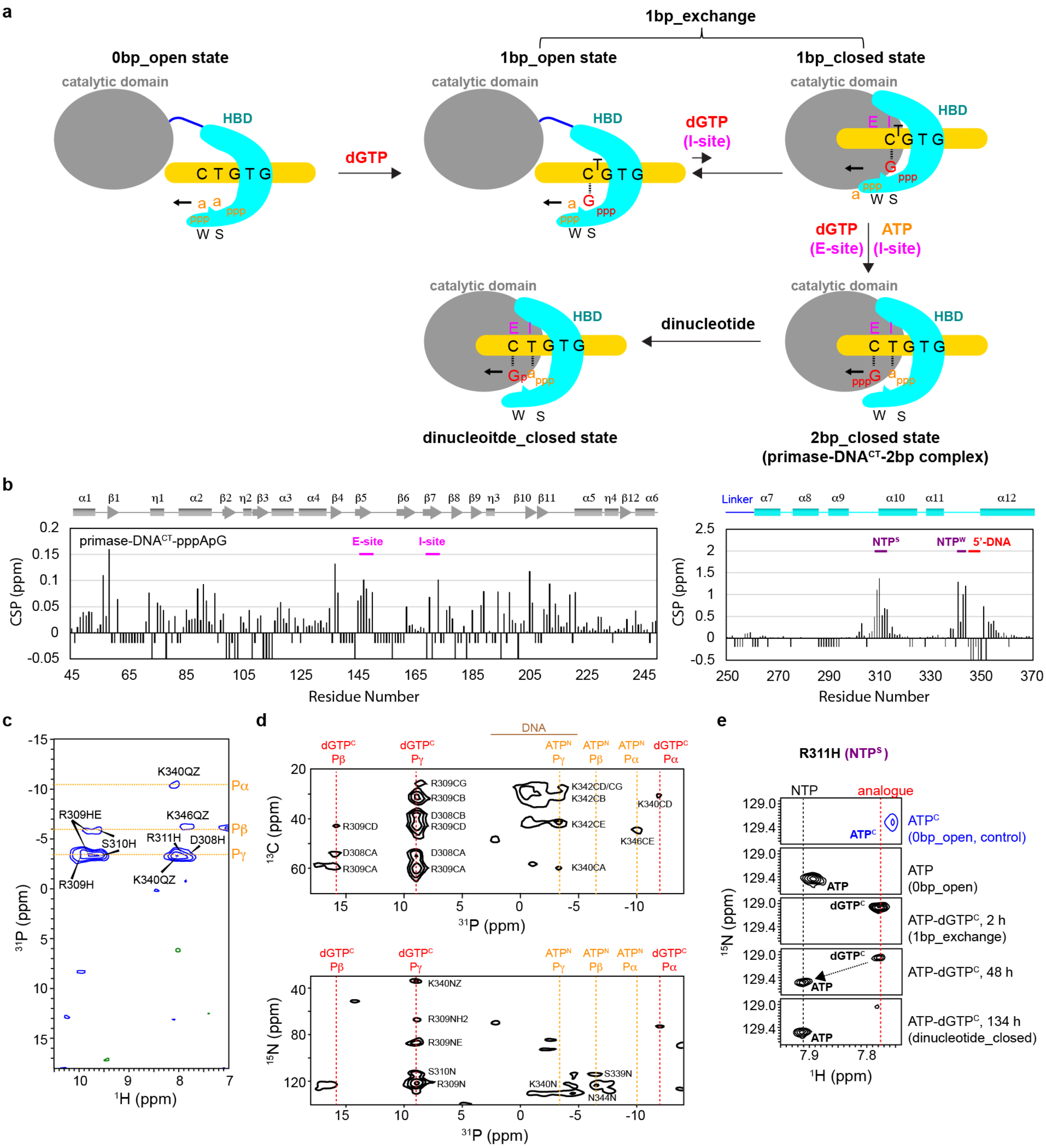

Supplementary Fig. 7 | Stepwise Assembly of the First Two NTPs during Dinucleotide Formation. a, Cartoon illustrating key conformational states. **b**, Bar plot of the CSPs for the primase-DNA^{CT} complex upon binding the pppApG dinucleotide, corresponding to the dinucleotide_closed state. **c,** 2D hPH solid-state NMR spectrum of the primase-DNA^{CT}-pNHppApG complex. Cross-peaks corresponding to the triphosphate group of the pNHppApG dinucleotide are indicated with orange dashed line. The dinucleotide was synthesized by pRN1 primase using ATP^{N} and dGTP^{C} as substrates. **d,** Solid-state NMR spectrum of the primase^{H145A}-DNA^{CT}-ATP^{N}-dGTP^{C} complex. Top: CHHP correlation spectrum. Bottom: NHHP correlation spectrum. Cross-peaks from dGTP^{C} are indicated by red dashed lines and labels, while those from ATP^{N} are marked with orange dashed line and labels. **e,** 2D ^{1}H-^{15}N HSQC spectra of the primase-DNA^{CT} complex with different NTPs, highlighting chemical shift

changes of the backbone amide of R311 at the NTP^S site of the HBD during distinct stages of dinucleotide formation. From top to bottom:

1) Complex with ATP^C, showing NTP analogue binding at the NTP^S site (0bp_open; control).
2) Complex with ATP, showing NTP binding at NTP^S the site (0bp_open).
3) Addition of $dGTP^C$ to the primase-DNA^{CT}-2ATP complex, showing $dGTP^C$ replacing ATP at the NTP^S site (2 h reaction; 1bp_exchange state).
4) Complex after 48 h of reaction with ATP and $dGTP^C$, showing the transition from one to two base pairs and subsequent dinucleotide formation, as $dGTP^C$ leaves and ATP rebinds at the NTP^S site (transition from 1bp_exchange to dinucleotide_closed state)
5) Final stage (134 h), showing the triphosphate of the pppApG dinucleotide, derived from ATP, bound at the NTP^s site (dinucleotide_closed state).

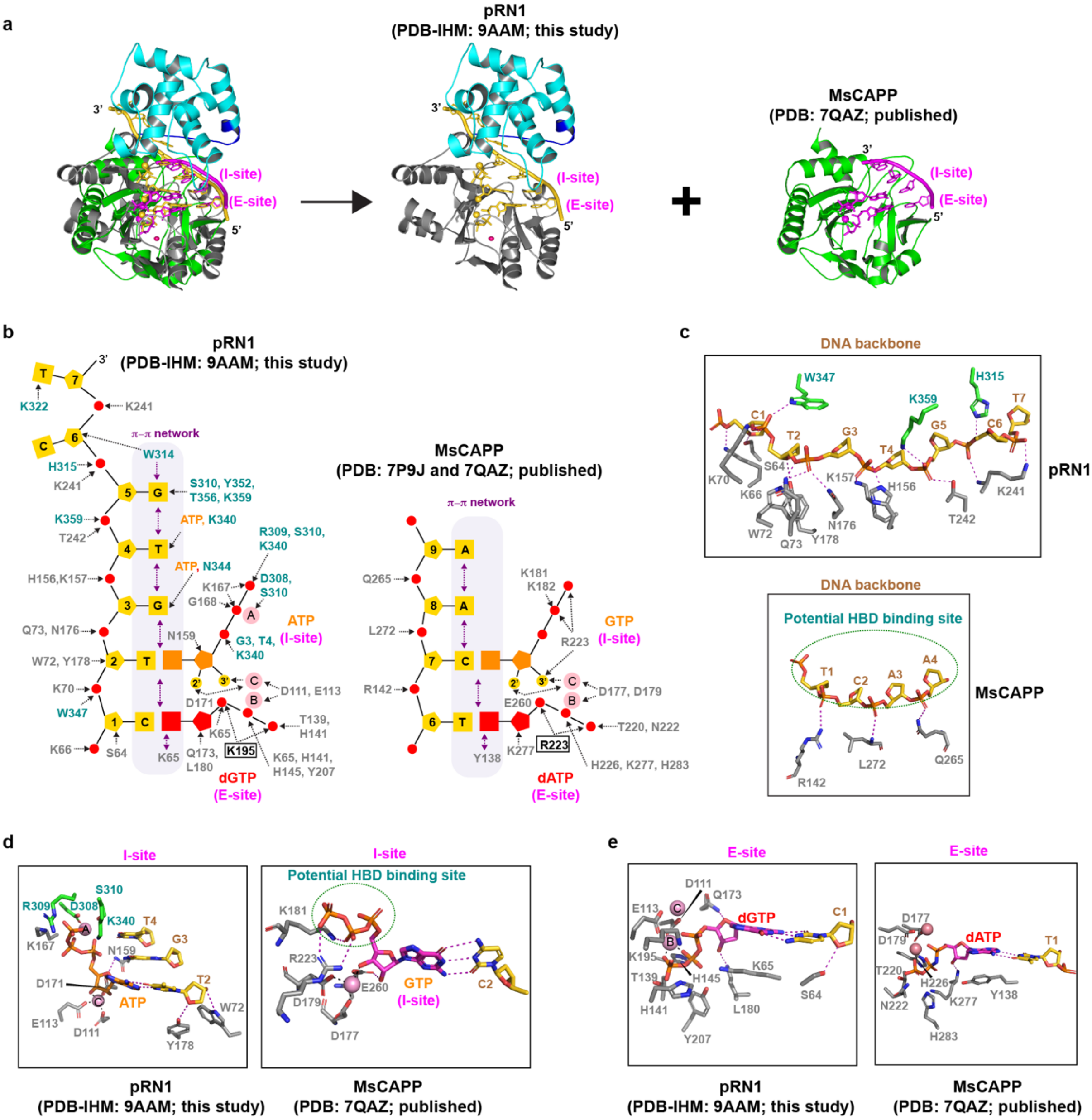

Supplementary Fig. 8 | Structural comparison of pRN1 and MsCAPP primases with two base-pairs bound in the active site. a, Aligned structural model of pRN1 primase-DNA^{CT}-2bp with the crystal structure of the catalytic domain of MsCAPP primase complexed with two base-pairs in the active site. **b**, Schematic representation of protein-DNA and protein-nucleotide contacts. **c**, Detailed view of the protein-DNA backbone interactions. **d**, Close-up of the I-site binding pocket. **e**, Close-up of the E-site binding pocket.

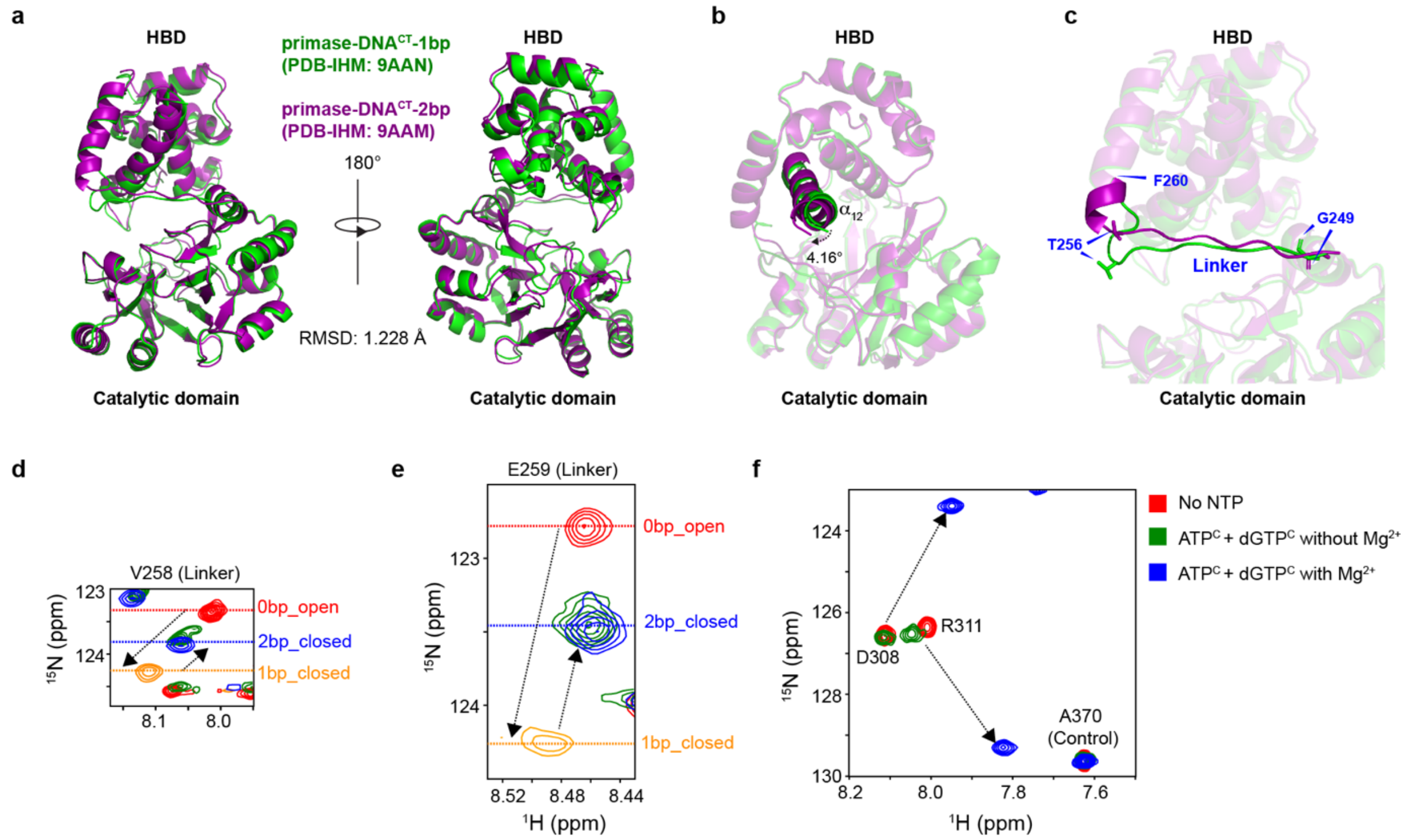

Supplementary Fig. 9 | Structural Comparison between Single- and Double-Base-Pair Binding States. a, Structural superposition of the primase-DNA^{CT}-1bp (green) and primase-DNA^{CT}-2bp (purple) complexes. **b**, Comparation of terminal helix orientations. **c**, Structural comparison of the linker region. **d-e**, Overlaid spectra of V258 (**d**) and E259 (**e**) in primase-DNA^{CT} complexes with ATP (red, 0bp_open state), dGTP (orange, 1bp_closed state), 3'-dATP and dGTP (green, 2bp_closed state), and pppApG dinucleotide (blue, dinucleotide_closed state). f, Overlaid 2D ^{1}H-^{15}N HSQC spectra of the HBD-DNA^{CT} complex in the absence of NTP (red), with ATP^{C} and dGTP^{C} but without Mg^{2+} (green), and with ATP^{C} and dGTP^{C} in the presence of Mg^{2+} (blue). In the NMR structure of the HBD-DNA^{CT}-ATP-dGTP complex, D308 coordinates MgA at the NTP^{S} site, while R311 engages the NTP triphosphate.

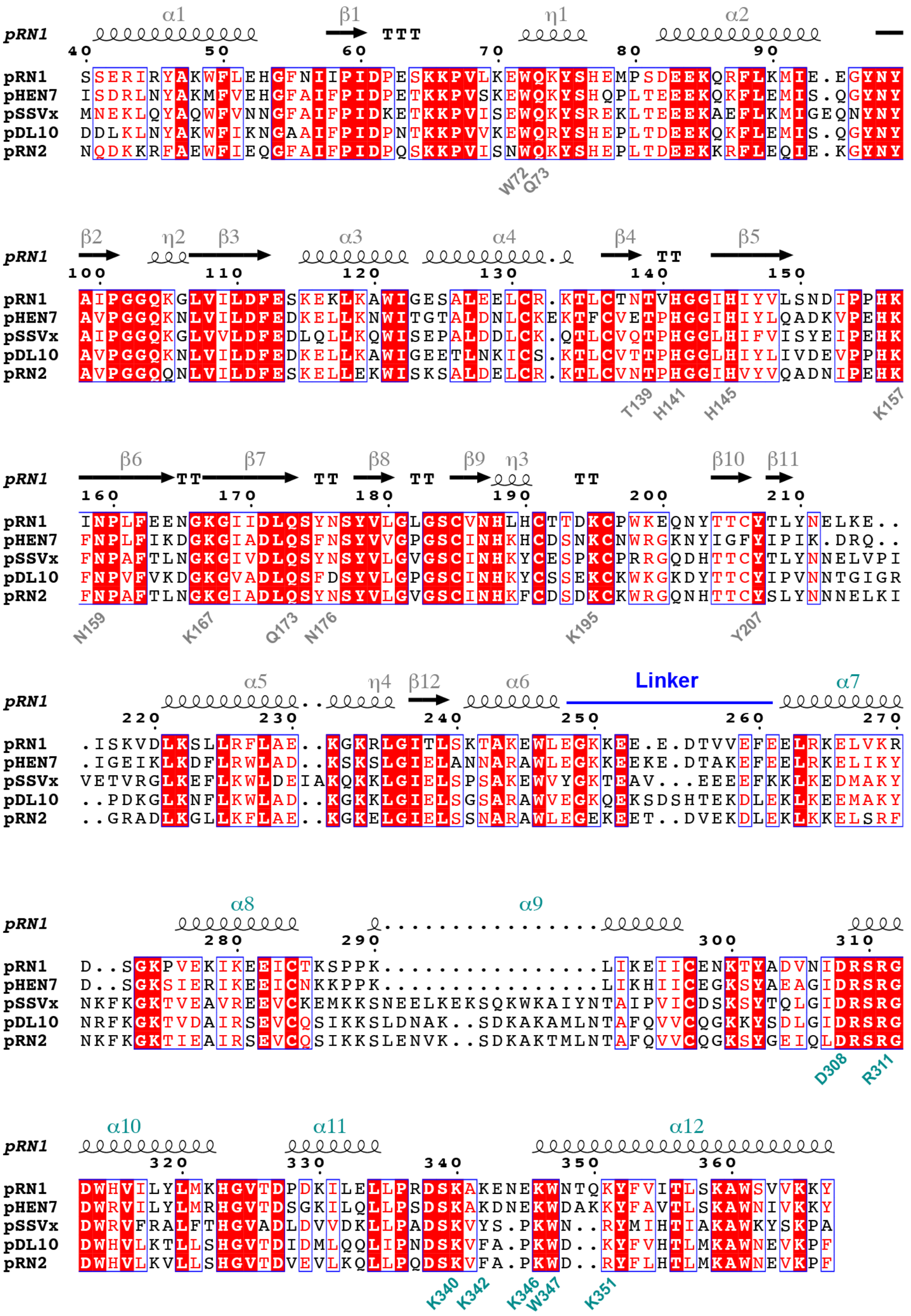

Supplementary Fig. 10 | Sequence alignment of primases from archaeal plasmids. Secondary structure elements (e.g., α1, β1) of the pRN1 primase, based on its crystal structure (PDB: 3M1M), are indicated above the alignment. Regions corresponding to the catalytic domain, linker, and HBD are labeled in gray, blue, and cyan, respectively. Key residues in the catalytic domain that form hydrogen bonds with DNA and NTPs in the pRN1 primase-DNA^{CT}-2bp complex, but have not previously been shown by mutagenesis to be essential for DNA binding or enzymatic activity, are labeled and highlighted in gray. Some key residues in the HBD that interact with two NTPs and the T2 base of the DNA template are labeled and highlighted in cyan. The GenBank accession numbers for the sequences are pRN1 (10954551), pHEN7 (11137542), pSSVx (7288084), pDL10 (7521320) and pRN2 (10954590).

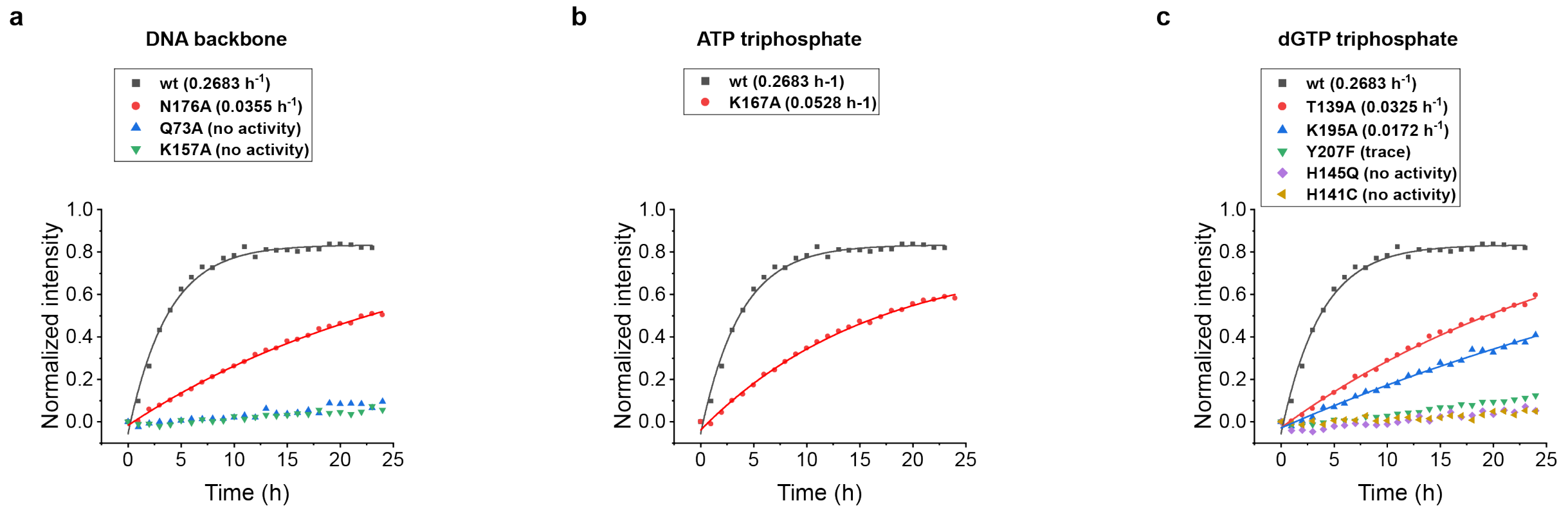

Supplementary Fig. 11 | Impact of key residue substitutions on primase activity. Time-dependent normalized intensities of dinucleotide formation showing the effects of disrupting interactions with specific substrate components: (**a**) DNA backbone, (**b**) ATP triphosphate, and (**c**) dGTP triphosphate. All reactions were carried out using the DNA^{CT} template and its cognate NTPs, ATP and dGTP.

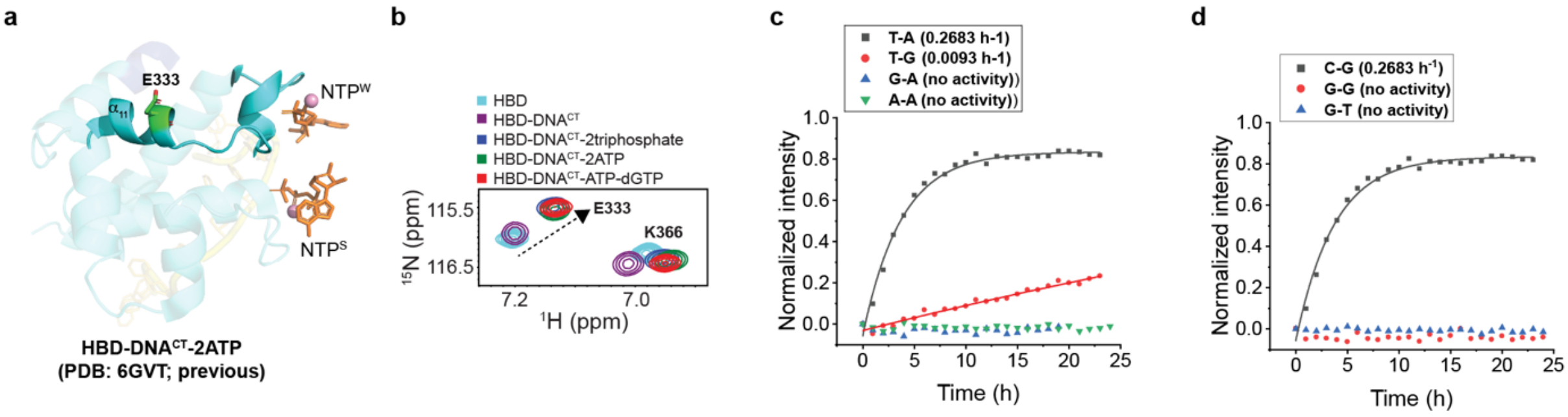

Supplementary Fig. 12 | Proofreading and DNA template selection. a, Location of E333 in the NMR structure of the HBD-DNA^{CT}-2ATP complex. **b**, Chemical shift analysis of E333 upon binding of a triphosphate or NTP to the HBD. **c**, Time-dependent normalized intensity of dinucleotide formation with a canonical Watson-Crick base pair at the E-site and different base pair configurations at the I-site. Reactions were carried out using the following I-site base pairs: T-A (control, black), T-G (red), G-A (blue), and A-A (green). **d**, Time-dependent normalized intensity of dinucleotide formation with a canonical Watson-Crick base pair at the I-site and different base pair configurations at the E-site. Reactions were carried out using the following E-site base pairs: C-G (control, black), G-G (red) and G-T (blue).

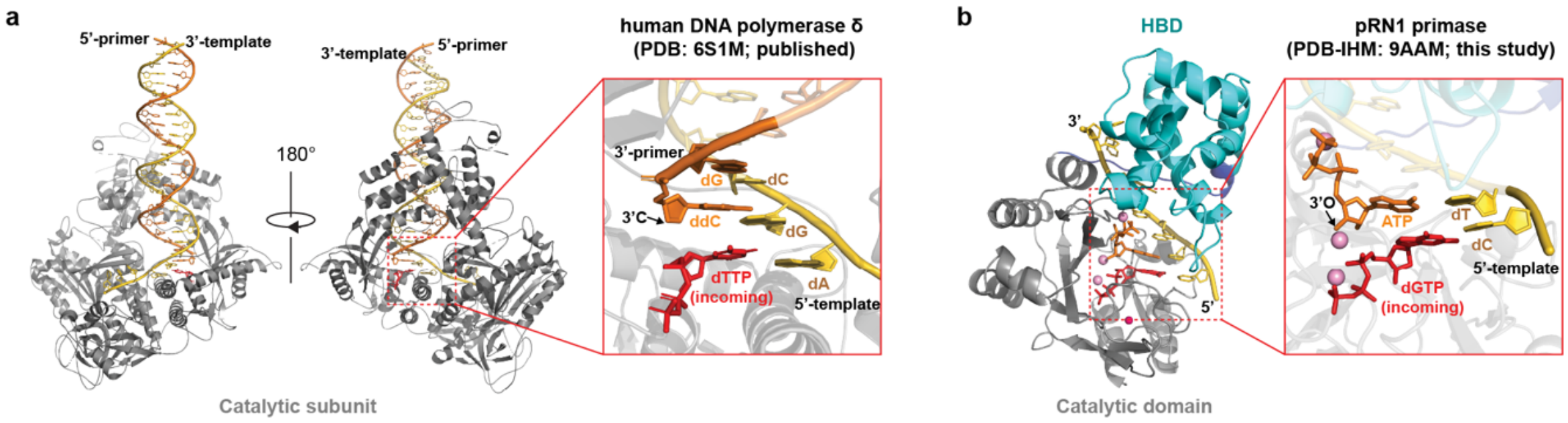

Supplementary Fig. 13 | Comparison of 3'-OH positioning at the I-site by DNA polymerase and primase. a, Cryo-EM structure of the catalytic subunit (p125) of human polymerase δ bound to a template-primer duplex and an incoming dTTP (PDB: 6S1M; published). **b**, NMR structrual model of the pRN1 primase-DNA^{CT}-2bp complex (PDB-IHM: 9AAM; this study).

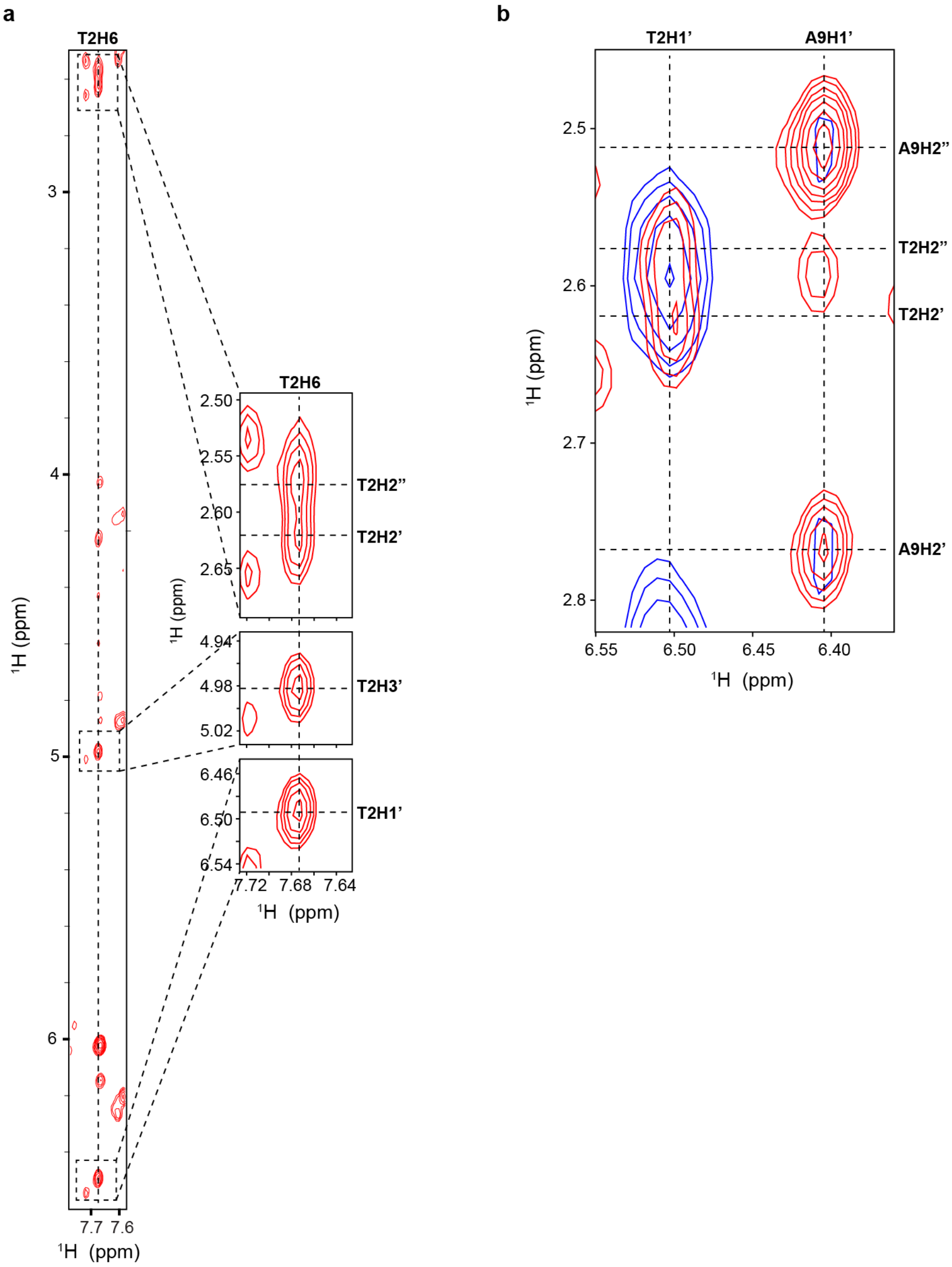

Supplementary Fig. 14 | Glycosidic conformation and sugar pucker of T2. a, Selected regions of the 2D F1fF2f NOESY spectrum of the HBD-DNA^{CT}-ATP^{C}-dGTP^{C} complex. The left panel shows intraresidue NOE cross-peaks between T2 H6 and the ribose protons of T2. The right panel zooms in and highlights the NOEs between T2 H6 and H1', H2', H2", and H3'. **b,** Selected regions of overlaid the 2D ^{1}H-^{1}H TOCSY (blue) and 2D F1fF2f NOESY (red) spectra of the HBD-DNA^{CT}-ATP^{C}-dGTP^{C} complex. Cross-peaks between H1' and H2'/H2" are shown for T2, with the corresponding signals for A9 included as a control.

Supplementary Table 1. Structural statistics

**NMR structure or model**	**HBD-DNA^{CT}-ATP-dGTP**	**primase-DNA^{CT}-1bp**	**primase-DNA^{CT}-2bp**
PDB or PDB-IHM ID	PDB: 9RTJ	PDB-IHM: 9AAN	PDB-IHM: 9AAM
Distance restraints	4168	6076	5636
DNA Intramolecular	273	307	279
intraresidual	203	241	202
sequential (|i-j|=1)	58	54	74
medium range (1<|i-j|<5)	12	12	3
long range (|i-j|>=5)	0	0	0
Protein Intramolecular	3584	5413	5103
intraresidual	768	1030	989
sequential (|i-j|=1)	801	1319	1255
medium range (1<|i-j|<5)	1094	1492	1402
long range (|i-j|>=5)	873	1415	1295
hydrogen bonds[a]	48	157	162
Complex Intermolecular	283	302	226
NOEs, solid-state data	267	252	161
hydrogen bonds	16	50	59
Torsion Angles[b]			
DNA sugar pucker (DELTA)	8	8	8
DNA glycosidic bond angle	1	1	0
Protein backbone	181	578	578
**Energy Statistics[c]**			
Average distance constraint violations			
0.1-0.2 A	84.8 +/- 28.6	207.9 +/- 69.8	210.7 +/- 70.8
0.2-0.3 A	8.0 +/- 3.6	28.5 +/- 10.6	19.0 +/- 7.1
0.3-0.4 A	2.4 +/- 1.0	3.8 +/- 1.8	1.0 +/- 0.8
>0.4 A	0.5 +/- 0.7	1.4 +/- 0.7	0.3 +/- 0.6
Maximal (A)	0.36 +/- 0.13	0.71 +/- 0.27	0.34 +/- 0.13
Average angle constraint violations			
<5 degree	10.1 +/- 4.0	79.5 +/- 27.3	84.1 +/- 28.7
>5 degree	2.5 +/- 10.9	2.7 +/- 10.9	2.5 +/- 10.9
Maximal (degree)	0.65 +/- 0.45	2.66 +/- 2.48	0.75 +/- 0.28
Mean AMBER Violation Energy			
Constraint (kcal.mol-1)	64.8 +/-21.9	180.1 +/-60.9	152.1 +/-50.9
Distance (kcal.mol-1)	64.6 +/-21.9	178.1 +/-60.2	150.8 +/-50.5
Torsion (kcal.mol-1)	0.2 +/-0.2	2.0 +/-1.0	1.4 +/-0.5
Mean AMBER Energy (kcal.mol-1)	-7417.9 +/- 2472.6	-14457.6 +/- 4819.2	-14552.1 +/- 4850.7
Mean deviation from ideal covalent geometry			
Bond Length (A)	0.0037 +/- 0.0012	0.0032 +/- 0.0011	0.0032 +/- 0.0011
Bond Angles (degrees)	1.505 +/- 0.502	1.400 +/- 0.467	1.391 +/- 0.464
**Ramachandran plot Statistics[c,d,e]**			
Residues in most favored regions (%)	85.9 +/- 1.5	89.1 +/- 1.0	91.8 +/- 0.6
Residues in additionally allowed regions (%)	13.1 +/- 1.4	10.3 +/- 1.1	7.5 +/- 0.6
Residues in generously allowed regions (%)	0.9 +/- 0.4	0.6 +/- 0.3	0.6 +/- 0.2
Residues in disallowed regions (%)	0.1 +/- 0.3	0.0 +/- 0.1	0.0 +/- 0.1
**RMSD to mean structure Statistics[c,d]**			
DNA			
Backbone atoms	0.39 +/- 0.15	0.78 +/- 0.28	0.81 +/- 0.31
Heavy atoms	0.38 +/- 0.14	1.25 +/- 0.36	1.23 +/- 0.37
Protein			
Backbone atoms	0.21 +/- 0.05	0.46 +/- 0.08	0.45 +/- 0.09
Heavy atoms	0.51 +/- 0.14	0.81 +/- 0.05	0.80 +/- 0.08
All molecules			
Backbone atoms	0.26 +/- 0.05	0.51 +/- 0.08	0.51 +/- 0.07
Heavy atoms	0.51 +/- 0.13	0.87 +/- 0.07	0.86 +/- 0.07

[a] H-bond constraints were identified from slow exchanging amide protons in D2O

[b] Protein backbone angles determined by the program TALOS+ and sugar pucker angles based on coupling efficiency in homonuclear TOCSY

[c] Statistics computed for the deposited bundle of 20 violation energy best structure selected out of 50 amber energy best

[d] Based on structured residue range as defined by user. Protein: 256-370, chain ID B (sequence range 256-370); DNA: 1-9, chain ID A (sequence range 1-9)

[e] Ramachandran plot, as defined by the program PROCHECK.

Supplementary Table 2. Experimental parameters of the solid-state NMR experiments.

**Sample**	pRN1 primase H145A-DNA^{CT}-ATP^{N}-dGTP^{C}	pRN1 primase H145A-DNA^{CT}-ATP^{N}-dGTP^{C}	pRN1 primase H145A-DNA^{CT}-ATP^{N}-dGTP^{C}
**Experiment**	NHHP	CHHP	hPH
**Figure**	Supplementary Fig. 7d	Supplementary Fig. 7d	Fig. 1e
ν_r / kHz	17.0	17.0	100.0
B_0/ T	11.7	11.7	28.2
transfer I	^{1}H-^{15}N CP	^{1}H-^{13}C CP	^{1}H-^{31}P CP
ν_1(^{1}H)/ kHz	45.0	60.0	70.0
ν_1(X)/ kHz	36.6	75.0	27.5
Shape	Tangent ^{1}H	Tangent ^{1}H	Tangent ^{1}H
time / ms	1.0	0.5	3.0
transfer II	^{15}N-^{1}H CP	^{13}C-^{1}H CP	^{31}P-^{1}H CP
ν_1(^{1}H)/ kHz	45.0	75.0	70.0
ν_1(X)/ kHz	36.6	60.0	27.5
Shape	Tangent ^{1}H	Tangent ^{1}H	Tangent ^{1}H
time / ms	1.0	0.5	3.0
transfer III	^{1}H spin-diffusion	^{1}H spin-diffusion	-
time / ms	0.2	0.2	-
transfer IV	^{1}H-^{31}P CP	^{1}H-^{31}P CP	-
ν_1(^{1}H)/ kHz	60.0	60.0	-
ν_1(X)/ kHz	43.7	59.1	-
Shape	Tangent ^{1}H	Tangent ^{1}H	-
time / ms	1.25	1.25	-
t_1 increments	56	96	128
spectral width (t_1) / kHz	7.0	25.0	24.3
acquisition time (t_1) / ms	3.9	1.9	2.6
t_2 increments	2048	3072	8192
spectral width (t_2) / kHz	100.0	100.0	55.6
acquisition time (t_2) / ms	10.2	15.4	73.7
^{1}H Decoupling sequence	Spinal64	Spinal64	swfTPPM
^{1}H Decoupling power / kHz	90.0	90.0	10.0
interscan delay / s	2.0	2.7	1.2
number of scans	9728	4608	2752

Supplementary Method

Methodology for Structural Modeling Based on NMR Data

To construct models of the pRN1 primase-DNA^{CT}-1bp and -2bp complexes, we used an integrated approach combining NMR data, structural insights from homologous proteins, and mutagenesis.

1. Design Concept

Our structural model was guided by comparing pRN1 primase with its homolog MsCAPP, which shares similar domain structures and conserved active-site residues. Both primases have an N-terminal catalytic domain essential for enzymatic activity and a C-terminal helix bundle domain (HBD). Key conserved residues in the active sites—D111, E113, and D171 in pRN1, corresponding to D177, D179, and E260 in MsCAPP—are vital for nucleotide coordination. Both primases recognize rNTPs in the I-site and dNTPs in the E-site. This functional conservation supports our hypothesis that these primases may employ similar mechanisms for base-pair binding, and structural insights from MsCAPP were thus transferable to pRN1 to build a model reflecting a conserved binding mechanism.

2. Modeling Strategy

To model the pRN1 primase-DNA^{CT}-1bp complex, we used the following six steps (Supplementary Fig. 5e):

Step 1: Initial Structural Alignment

We began modeling by aligning the catalytic domains of pRN1 and MsCAPP in PyMOL, focusing on nine conserved residues (in pRN1: 65, 66, 67, 111, 113, 145, 171, 178, and 180; in MsCAPP: 138, 139, 140, 177, 179, 226, 260, 275, and 277). These residues were chosen as they are functionally significant for nucleotide and substrate binding. Aligning only these

conserved residues minimized alignment distortions and provided a foundation for transferring two base pairs from MsCAPP into the catalytic domain of pRN1.

Step 2: Placement and Mutation of Key Base Pairs

Post-alignment, we removed non-essential components and retained the GTP-cytosine base pair in the I-site from MsCAPP. This pair was mutated to dGTP-cytosine to match pRN1 binding specificity. Any steric clashes were resolved in subsequent CYANA calculations, optimizing the nucleotide interactions specific to pRN1 while maintaining structural integrity.

Step 3: Regularization with CYANA and Pseudo-Atom Anchoring

Due to the integration of components from two different crystal structures, the combined model required regularization in CYANA. During this step, missing elements, such as linker residues (249 to 260) and additional nucleotides (e.g., ATP and sections of the DNA template), were added without constraints.

In this step, pseudo-atoms within a pseudo-residue, denoted as 'REG1,' were generated based on reference positions from the crystal structure. These REG1 pseudo-atoms are positioned to correspond with specific reference atoms from the crystal structure. By doing so, they anchor the model to a known structural framework, aligning the NMR-derived structure with crystal structure reference points. In subsequent CYANA calculations, upper distance restraints were applied between REG1 pseudo-atoms and corresponding reference atoms, confining these atoms within 0.3 Å. This setup allows the model to retain the overall alignment with the crystal structure but still permits slight adjustments, simulating conformational flexibility that might occur in solution.

Step 4: CYANA Calculation with Distance Restraints

After regularization, we introduced distance restraints from various sources to ensure structural accuracy:

a. **Apo pRN1 Primase Structure (PDB: 3M1M)**: Provided primary distance restraints for the catalytic domain backbone and secondary structure elements, maintained by retaining upper distance restraints around the REG1 pseudo-residue.

b. **MsCAPP Complex Structure (PDB: 7QAZ)**: Supplied base-pair positioning in the I-site and E-site, with restraints retained around REG1 for the base pair binding in the active site.

c. **NMR Structure of HBD-DNA^{CT}-ATP-dGTP Complex**: Defined HBD-DNA and ATP interactions, especially for the dGTP triphosphate, removing restraints around the REG1 and using the same distance restraints as applied in the original NMR structure.

Initial hydrogen bonds and metal ion interactions (to be discussed further) were added between catalytic residues and the C1-dGTP base pair to ensure accurate positioning. CYANA calculations were repeated to minimize side-chain conformational violations, particularly for residues in the I-site that differed between the apo and substrate-bound conformations. Incremental upper distance restraints between REG1 and side chains allowed adaptation to nucleotide binding without altering the backbone. Iterations continued until CYANA's target function stabilized with minimized violations.

Step 5: AMBER Refinement

AMBER refinement was used to achieve final structural stability by optimizing energy states and fine-tuning atomic interactions. For the catalytic domain and C1-dGTP base pair, due to the absence of a specific library for pseudo-residue REG1 in AMBER, proton distances within 5 Å , calculated from the CYANA-refined structure, were used as upper distance restraints. These restraints effectively simulated NOESY-derived constraints for maintaing atom proximity. For the HBD, DNA^{CT} template, ATP and the dGTP triphosphate, the same distance restraints applied in the NMR structure of the HBD-DNA^{CT}-ATP-dGTP Complex

were used. Multiple rounds of refinement progressively reduced energy levels, resulting in a model that maintained empirical distance restraint fidelity.

Step 6: Addition of New Hydrogen Bonds Identified in AMBER Refinement

During AMBER refinement, several hydrogen bonds were identified between catalytic domain residues and the bound C1-dGTP base pair. To validate these interactions:

a. **Conservation Analysis**: Highly conserved residues across archaeal primases were prioritized for hydrogen bond restraints, ensuring biological relevance.

b. **Restraint Impact Assessment**: CYANA target functions and AMBER energy levels were evaluated following the addition of each restraint. Restraints causing violations were discarded, while those stabilizing the model were retained.

c. **Experimental Validation via Mutagenesis**: Site-directed mutagenesis was used to confirm the functional importance of predicted interactions. If mutations significantly affected dinucleotide formation, the hydrogen bond was incorporated as a distance restraint in subsequent CYANA and AMBER calculations.

This process was repeated for the primase-DNA^{CT}-2bp complex, with adjustments for an additional base pair. Modifications included mutating bound nucleotides to reflect ATP-thymine and dGTP-cytosine interactions, aligning with DNA^{CT} template requirements.

3. Manually Added Hydrogen and Metal Ion Bonds

Due to limited conservation between pRN1 and MsCAPP primases, some interactions in the I-site and E-site required manual adjustment. Additional restraints, including hydrogen bonds and metal ion coordination, were added to stabilize base-pair positioning. These restraints for the primase-DNA^{CT}-2bp complex originated from three sources:

a. **Previous Structures or NMR Data (Supplementary Fig. 15**): Hydrogen bonds stabilizing alpha-helices and beta-sheets in the catalytic domain were derived from the apo pRN1 crystal structure, while those in the HBD came from the NMR structure of the HBD-DNA^{CT}-ATP-dGTP complex. Hydrogen bonds between the ATP triphosphate and the HBD or DNA template (G3 and T4) were based on observed interactions between the ATP triphosphate and the HBD or DNA template using solution- and solid-state NMR. Additional hydrogen bonds were inferred, such as between the O3A group of dGTP and residue K65 in the E-site of the catalytic domain. NMR analysis with dGMPCPP showed no chemical shift changes, suggesting that K65, stacked near dGTP, likely interacts with O3A. In MsCAPP, the analogous position is occupied by Y138, which, although similarly positioned, cannot form this bond due to its shorter side chain.

b. **Comparative Data from Other Primase Structures (Supplementary Fig. 16**): Distance restraints were informed by structures of the MsCAPP and human PrimPol complexes, reinforcing known interactions in primase structures.

c. **AMBER Refinement Observations (Supplementary Fig. 17**): Most of manually added hydrogen and metal ion bond restraints were from the two sources above. Additional restraints were also identified during AMBER refinement. Hydrogen bonds were added only after validation in Step 6 above.

During CYANA calculations and AMBER refinement, we observed that the 5'-end of the DNA consistently moved away from the catalytic domain. This displacement occurred due to insufficient distance restraints to firmly position the 5'-end within the catalytic domain. This movement lowered the CYANA target function and AMBER energy, as the software optimized for lower values, inadvertently favoring this shift. To prevent this unintended displacement, we introduced additional distance restraints: specifically, between the ribose of

T2 and the backbone of K66, as well as between the ribose of C1 and the backbone of K65. These restraints helped maintain the spatial positioning of the DNA's 5'-end relative to the catalytic domain, enhancing model stability.

The restraints for the primase-DNA^{CT}-1bp complex were applied similarly to those used for the primase-DNA^{CT}-2bp complex.

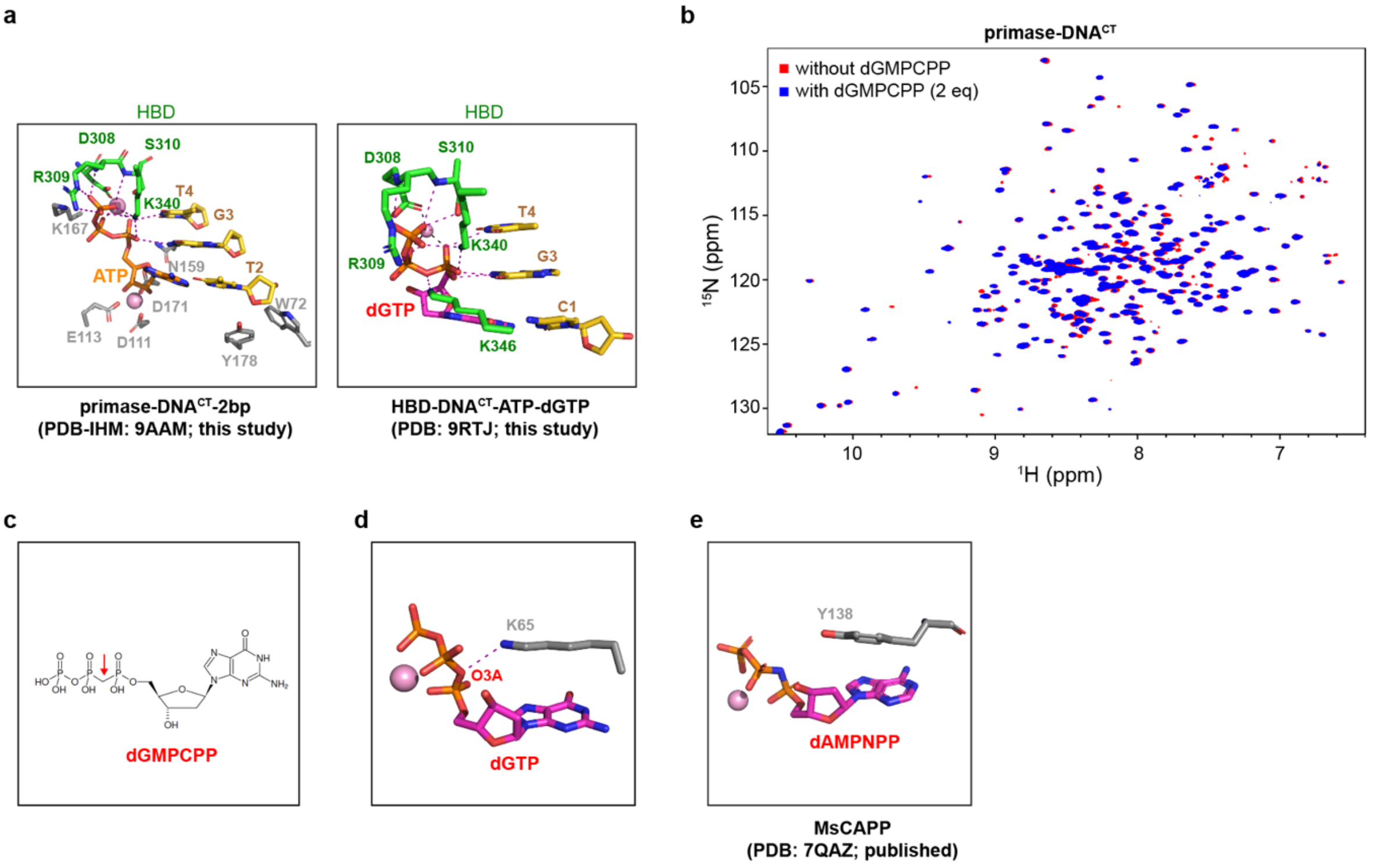

Supplementary Fig. 15 | Manually Added Hydrogen Bonds Derived from Previous Structure or NMR data. a, The hydrogen bonds between the ATP triphosphate and the HBD or DNA template (G3 and T4) (left panel), shown as purple dashed lines, were derived from the hydrogen bonds observed between the dGTP triphosphate and the HBD or DNA template in the NMR structure of the HBD-DNA^{CT}-ATP-dGTP complex (right panel). Residues from the catalytic domain that are also hydrogen bonds with the ATP triphosphate are depicted as sticks, but these bonds are not shown with dashed lines. **b**, Overlaid 2D ^{1}H-^{15}N HSQC spectra of the primase-DNA^{CT} complex in the absence (red) and presence (blue) of dGMPCPP, highlighting chemical shift changes. **c**, The structural formula of dGMPCPP. **d**, In the E-site

of the primase-DNA^{CT}-2bp complex, dGTP is stacked by K65. A hydrogen bond is formed between the O3A group of dGTP and the side chain of K65. **e**, In the E-site of the MsCAPP complex crystal structure, dAMPNPP is stacked by Y138.

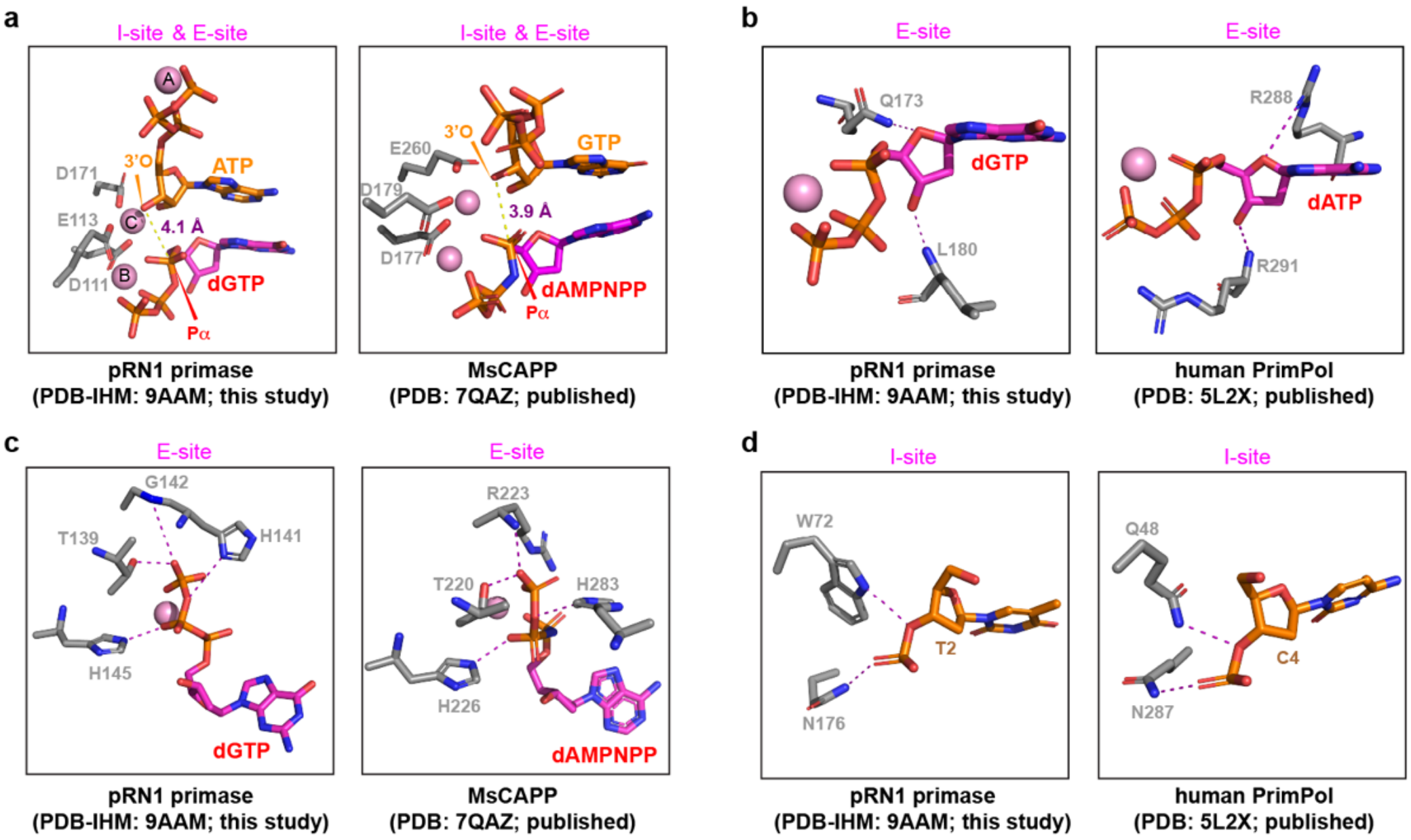

Supplementary Fig. 16 | Manually Added Hydrogen Bonds and Metal Ion Bonds Derived from the MsCAPP Complex and Human PrimPol Complex. a, Metal ion bonds formed between magnesium ions B and C, the active-site residues D111, E113, and D171, and the two NTPs. These bonds are derived from the crystal structure of the MsCAPP complex with two base pairs. **b**, Two hydrogen bonds between the dGTP ribose and the E-site residues are shown: the Q173 side-chain hydrogen bond to the dGTP O4', and the L180 backbone hydrogen bond to the dGTP 3'-OH group. These are derived from the crystal structure of human PrimPol. **c**, Four hydrogen bonds within the dGTP triphosphate are derived from the crystal structure of MsCAPP. **d**, Two hydrogen bonds formed in the T2 bound in the I-site are derived from human PrimPol.

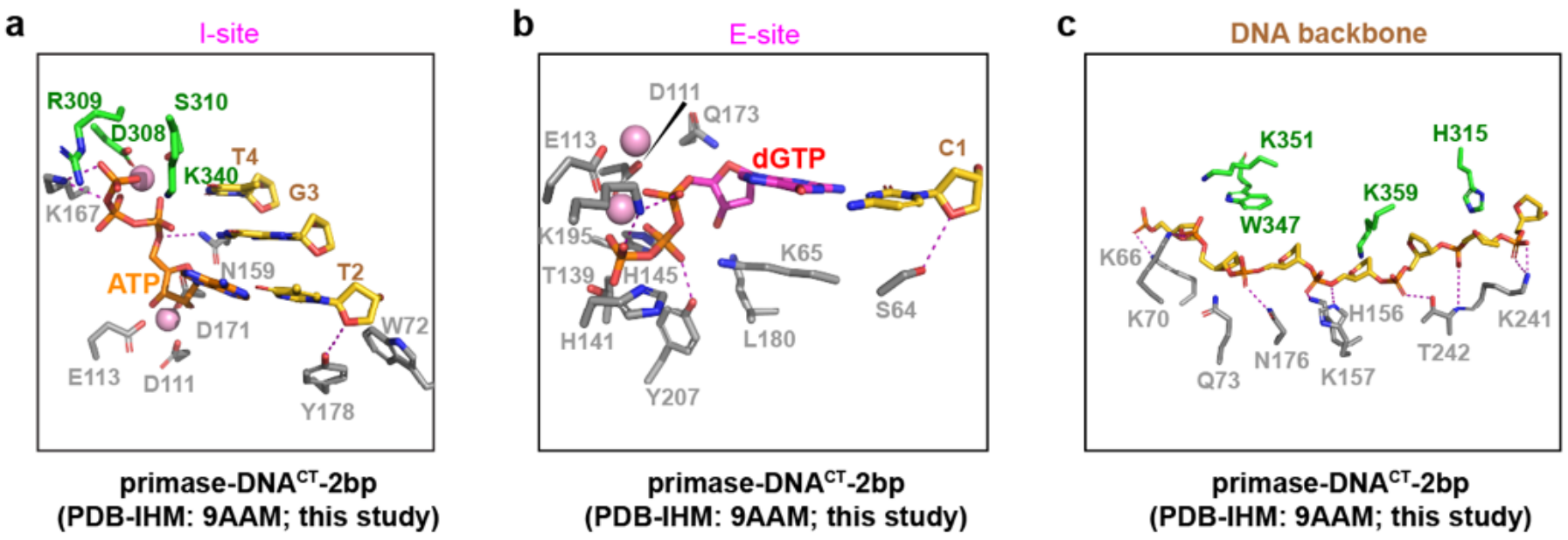

Supplementary Fig. 17 | Manually Added Hydrogen Bonds Identified during AMBER Refinement. a, Three hydrogen bonds between the ATP-T2 base pair in the I-site and catalytic domain residues K167, N159, and Y178 were identified during the refinement and are shown as purple dashed lines. **b**, Four hydrogen bonds between the dGTP-C1 base pair in the E-site and catalytic domain residues S64, Y207, and K195 were found during the refinement and are shown as purple dashed lines. **c**, All hydrogen bonds between the DNA backbone and catalytic domain residues, except for the one formed by Q73, were identified during the refinement and are shown as purple dashed lines. Other residues involved in hydrogen bonds, derived from NMR data or from the MsCAPP and human PrimPol complexes, are shown as sticks but without purple dashed lines.
