# Supplementary material for: Structural and mechanistic insights into primer synthesis initiation by DNA primase
Source: Nat Commun. 2026 Jul 3;17:8299. doi: 10.1038/s41467-026-74862-8 (PMC13469122; doi:10.1038/s41467-026-74862-8)
Supplement: Supplementary file 2 [file 41467_2026_74862_MOESM6_ESM.pdf]

nature portfolio

Corresponding author(s): Frédéric Allain and Pengzhi Wu

Last updated by author(s): May 31, 2026

Reporting Summary

Nature Portfolio wishes to improve the reproducibility of the work that we publish. This form provides structure for consistency and transparency in reporting. For further information on Nature Portfolio policies, see our Editorial Policies and the Editorial Policy Checklist.

Statistics

For all statistical analyses, confirm that the following items are present in the figure legend, table legend, main text, or Methods section.

n/a	Confirmed	
☐	☒	The exact sample size (*n*) for each experimental group/condition, given as a discrete number and unit of measurement
☒	☐	A statement on whether measurements were taken from distinct samples or whether the same sample was measured repeatedly
☒	☐	The statistical test(s) used AND whether they are one- or two-sided<br>*Only common tests should be described solely by name; describe more complex techniques in the Methods section.*
☒	☐	A description of all covariates tested
☒	☐	A description of any assumptions or corrections, such as tests of normality and adjustment for multiple comparisons
☒	☐	A full description of the statistical parameters including central tendency (e.g. means) or other basic estimates (e.g. regression coefficient) AND variation (e.g. standard deviation) or associated estimates of uncertainty (e.g. confidence intervals)
☒	☐	For null hypothesis testing, the test statistic (e.g. *F*, *t*, *r*) with confidence intervals, effect sizes, degrees of freedom and *P* value noted<br>*Give P values as exact values whenever suitable.*
☒	☐	For Bayesian analysis, information on the choice of priors and Markov chain Monte Carlo settings
☒	☐	For hierarchical and complex designs, identification of the appropriate level for tests and full reporting of outcomes
☒	☐	Estimates of effect sizes (e.g. Cohen's *d*, Pearson's *r*), indicating how they were calculated

Our web collection on statistics for biologists contains articles on many of the points above.

Software and code

Policy information about availability of computer code

Data collection	TopSpin 4.0.7, MicroCal PEAQ-ITC Analysis
Data analysis	TopSpin 4.0.7, NMRFAM-Sparky , CYANA 3.98, AMBER 21, MOLMOL, PYMOL 3.1.6.1, Adobe Illustrator 2025, Adobe Premiere Pro 2025, OriginPro 2025, MicroCal PEAQ-ITC Analysis

For manuscripts utilizing custom algorithms or software that are central to the research but not yet described in published literature, software must be made available to editors and reviewers. We strongly encourage code deposition in a community repository (e.g. GitHub). See the Nature Portfolio guidelines for submitting code & software for further information.

Data

Policy information about availability of data

All manuscripts must include a data availability statement. This statement should provide the following information, where applicable:

- Accession codes, unique identifiers, or web links for publicly available datasets
- A description of any restrictions on data availability
- For clinical datasets or third party data, please ensure that the statement adheres to our policy

The NMR structure of the HBD-DNA-ATP-dGTP complex, along with the restraints used for its calculation, have been deposited in the Protein Data Bank (PDB, https://www.wwpdb.org/) under accession code 9RTJ. The corresponding chemical-shift values are available in the Biological Magnetic Resonance Data Bank (BMRB, https://bmrb.io) under accession number 35007. Structural models of the primase-DNA-1bp and primase-DNA-2bp complexes have been deposited in the

Protein Data Bank – Integrative/Hybrid Methods (PDB-IHM, https://pdb-ihm.org/) under accession codes R6FP and MBNP, respectively. The chemical-shift values and restraints used for these models are available in the BMRB under accession numbers 53262 and 53266, respectively.

Research involving human participants, their data, or biological material

Policy information about studies with human participants or human data. See also policy information about sex, gender (identity/presentation), and sexual orientation and race, ethnicity and racism.

Reporting on sex and gender	Sex and gender were not considered in this study. The study did not involve human participants or vertebrate animals, and therefore sex- and gender-related information was not collected or applicable.
Reporting on race, ethnicity, or other socially relevant groupings	Race, ethnicity, or other socially relevant groupings were not considered in this study. The study did not involve human participants, and therefore such information was not collected or applicable.
Population characteristics	Not applicable. See above.
Recruitment	Not applicable. See above.
Ethics oversight	Not applicable. See above.

Note that full information on the approval of the study protocol must also be provided in the manuscript.

Field-specific reporting

Please select the one below that is the best fit for your research. If you are not sure, read the appropriate sections before making your selection.

☒ Life sciences ☐ Behavioural & social sciences ☐ Ecological, evolutionary & environmental sciences

For a reference copy of the document with all sections, see nature.com/documents/nr-reporting-summary-flat.pdf

Life sciences study design

All studies must disclose on these points even when the disclosure is negative.

Sample size	No statistical sample-size calculation was performed. The study is based on structural determination and biochemical assays, not hypothesis-driven comparisons between groups. Experiments were designed to obtain sufficient signal for reproducible structural and functional characterization.
Data exclusions	No data were excluded from the analyses. All collected data were included in the analyses. For structural calculations, all spectra and replicates were used in model building and validation.
Replication	Experiments were replicated. ITC assays were repeated independently at least twice with consistent results. NMR and structural experiments were repeated or validated through independent measurements to ensure reproducibility.
Randomization	Not applicable. The study did not involve allocation of subjects or samples to experimental groups, as all experiments were performed in vitro using purified proteins and nucleic acids.
Blinding	Not applicable. No subjective outcome assessments were performed. Structural analyses and biochemical measurements were objective and did not require blinding.

Reporting for specific materials, systems and methods

We require information from authors about some types of materials, experimental systems and methods used in many studies. Here, indicate whether each material, system or method listed is relevant to your study. If you are not sure if a list item applies to your research, read the appropriate section before selecting a response.

Materials & experimental systems

n/a	Involved in the study
☒	☐ Antibodies
☒	☐ Eukaryotic cell lines
☒	☐ Palaeontology and archaeology
☒	☐ Animals and other organisms
☒	☐ Clinical data
☒	☐ Dual use research of concern
☒	☐ Plants

Methods

n/a	Involved in the study
☒	☐ ChIP-seq
☒	☐ Flow cytometry
☒	☐ MRI-based neuroimaging

Plants

Seed stocks	Not applicable. No plant material or seed stocks were used in this study.
Novel plant genotypes	Not applicable. The study did not involve generation or analysis of plant genotypes.
Authentication	Not applicable. No plant material was used. Therefore, anthentication procedures were not required.
