# Supplementary material for: Structural and mechanistic insights into primer synthesis initiation by DNA primase
Source: Nat Commun. 2026 Jul 3;17:8299. doi: 10.1038/s41467-026-74862-8 (PMC13469122; doi:10.1038/s41467-026-74862-8)
Supplement: Supplementary file 3 [file 41467_2026_74862_MOESM7_ESM.pdf]

nature portfolio

Peer Review File

Structural and Mechanistic Insights into Primer Synthesis Initiation by DNA Primase

Corresponding Author: Professor Frédéric Allain

This file contains all reviewer reports in order by version, followed by all author rebuttals in order by version.

Attachments originally included by the reviewers as part of their assessment can be found at the end of this file.

Version 0:

Reviewer comments:

Reviewer #1

(Remarks to the Author)
See attachment.

Reviewer #2

(Remarks to the Author)
260204primase_review

The primase work by Wu and colleagues carried out NMR work that indicated a novel, unexpected mechanism for initiation of priming, a major question in the field. Their data was complex, with nucleotides binding in unexpected places and a flipped out base in the template strand right where priming should be occurring. The mechanisms revealed by this study are important and would be of interest to Nature Comm readers and the replication and repair fields.

The major concern is the paper's lack of clarity in correlating the structural models (CIFs/PDBs) with the experimental NMR data (e.g., chemical shifts, NOEs, RDCs) used to determine them. The authors appear to shift between discussing determined structures and discussing unassigned or dynamically averaged NMR data, creating significant ambiguity. This makes it challenging for non-specialists in solution NMR (including many structural biologists) to critically evaluate the mechanisms

Comments
For readers to immediately assess the significance of this work, please add the organism from which the primase is derived in the abstract and in the Fig. 1 legend. Along the same lines, it would be helpful to add for the general audience that the PRN1 plasmid provides a minimalist primase system. Use of an extremophile model enables many structural insights, but it should be mentioned.
The authors refer to 3 structures provided by this work, but only two CIFs are located. HBD-DNA-ATP
dGTP seemed to be missing. Figuring out which structure was which was confusing. Adding the assigned pdb deposition ID for structures determined by this paper to figures, including extended data figures and supplementary tables, would improve clarity from which coordinates the figures are derived.
There appears to be NMR data where the structures were not determined. How feasible would it be to determine the entire structure, as small changes may be propagated outside of the binding site?
Figure 1. It was difficult to reconcile the HBD construct shown in Fig. 1 with the cif file of the full-length protein provided. If the cif file had only the HBD construct, then it would make sense. In the methods, it seems that the authors collected NMR data on both the truncated construct and the full-length. Please clarify or provide individual structures for each independent data set, if the truncated was collected as truncation could alter conformational dynamics in the protein. Throughout it seemed that the authors shifted easily between the two, making it difficult to follow.
Fig 1 Metal spheres are not defined at any point The authors have not described that they removed sections of the DNA bases. For the former, it was not until I looked at the cif files directly that I realized.
Depth cueing in Fig 1 j makes it look like the lysines are coordinating with the metal.
The authors do not make it clear that the structure described in Fig. 2 was previously published in Cell. What is new?

To know the generality of the findings,an MSA this archaeal model system compared to the human, would show the conservation of key residues. The authors have an MSA with other archaeal plasmid-based systems that could be used to see close ortholog conservation. It would be helpful for readers to denote any residues mentioned in the text or regions, such as domains and the linker.
The reported 5 Å phosphodiester-phosphodiester distance in the flipped-out T2 structure, when modeled with an expected C2'-endo sugar pucker, is structurally unusual and potentially incompatible with standard nucleic acid geometry. Please provide the primary experimental evidence.
The authors report that they mutated the linker residues to remove hydrophobic, yet they chose lysine, known in protein structures for the hydrophobility of the aliphatic chain. Their choice of mutation did alter beta branching and electron dispersion. The authors argue the biological significance of the flipped out T2. Mutation of the linker partly addressed it, but a more precise mutation (e.g. K351) may be more selective in its effect.
In discussion of unexpected preference for pyrimidines, the other side (template) are purines. Could stabilization and stacking of the purines in the template strand be an important consideration?

Reviewer #3

(Remarks to the Author)

Reviewer #4

(Remarks to the Author)
The manuscript by Wu et al. describes a detailed structural and mechanistic study of primer initiation (dinucleotide synthesis) by the archaeo-eukaryotic primase pRN1. Through DNA-dependent de novo synthesis, primases perform the essential role of generating the RNA primers required to begin replicative DNA synthesis. Based primarily on NMR experiments, the authors propose a complete model for all steps of primer initiation. The supporting data appear to be of high quality, the manuscript is well written, and the figures are clear. However, in my opinion, a number of issues need to be addressed before the manuscript will be suitable for publication in Nature Communications.

1. The model presented is well supported but is not proven directly by experiment. Therefore, the title should be scaled back, perhaps to specifically state ‘a model for’ as opposed to ‘molecular basis’. The tendency to over-interpret the fine details and overstate the certainty of the model is evident throughout the manuscript. A clear example is the passage starting on line 362: the discussion of a 4 degree shift in the orientation of a helix from a comparison of structural models represents an over-interpretation and is misleading because this is overly precise given the intrinsic uncertainty in the accuracy (and possibly the precision) of the structures.

In this vein, the conclusions about key interactions are largely derived from secondary analysis, i.e. NMR structures calculated from the NMR data but not direct NMR evidence. This gives rise to a certain degree of uncertainty in the mechanistic insights and models presented. This is not a fundamental flaw, there are a number of useful insights and an interesting model presented. However, the extent of uncertainty does need to be clearly acknowledged in the Results and Discussion sections.

2. The initiation mechanism described for pRN1 is claimed to be ‘highly likely conserved across all primases’ and ‘likely conserved among all known primases’. Such a broad generalization made with such a high degree of certainty seems unwarranted. These statements should be moderated. Also, it would be helpful if the authors clarified which features of their model that they believe to be shared by other primases, and by which other primases. Some features, such as recognition of a specific sequence and transient flipping of a specific nucleotide, may be shared by more closely related enzymes but may not be universal.

3. The use of NMR-based RNA priming assays would be significantly strengthened if results were compared to the significant literature of gel-based biochemical RNA priming assays.

4. While the clear focus of the study is the mechanism of primer initiation, the manuscript would be more impactful if the Discussion included speculation about primer elongation and termination, as introduced on line 38. Of particular interest, and following from the results described in the manuscript, are the roles played by the ancillary domains during these subsequent stages.

Additional issues relating to specific statements in the text:

Lines 31-43: ‘DNA primase’ and ‘DNA polymerase’ should be plural not singular.

Lines 59,69: These are strange uses of the term apo.

Line 76: Please clarify if this statement specifically refers to MsCAPP. If this refers to all primases, the statement is incorrect.

Line 102: This is a strange use of the term wild-type.

Line 221: ‘changed’ should be ‘changes’

Lines 258-259: The statement regarding closed conformations should be modified to acknowledge the two such structures of human primase that have been determined (He et al., Nature 2022 and Cordoba et al., J. Mol. Biol. 2023)

Lines 282-284: The meaning of this sentence is unclear and should be clarified.

Lines 303-304: The optimal selection of a representative structure from an ensemble is the one structure that is closest to the mean of the ensemble.

Lines 385-389: This section is vague. Which residues were mutated? How is mutation of these residues believed to impair dinucleotide formation?

Line 387: ‘confirm’ should be scaled back to ‘are consistent with’

Line 482: poorly understood structurally

Lines 482-483: One recent model was proposed in Cordoba et al., J. Mol. Biol. 2023.

Version 1:

Reviewer comments:

Reviewer #1

(Remarks to the Author)
The authors have satisfactorily addressed my concerns in the revised submission.

Reviewer #2

(Remarks to the Author)
Wu et al. have substantially clarified their work in response to reviewer comments. Clearer labeling of newly determined and previously published structures greatly improved interpretation of the figures and conclusions. The mechanism of primase initiation is inherently complex, and if the proposed model is correct, this work provides compelling structural and mechanistic insight into how base-pairing fidelity is enforced during primer synthesis. One outstanding concern regarding the NMR structure remains is described below.

1. One major concern is that the flipped out base, mentioned in the abstract, may be an artifact. As noted in the text, stabilization on the surface is through Met255 and Lys351, as well as hydrophobics in two regions 257-258 and 354-355. Met255 plays a primary role, but Met255 is not an actual primase residue but an artifact of the TEV linker. Is there any evidence, outside of the reported NMR structure, that the T flips out? The K351A mutation that abolished DNA and NTP binding might not have anything to do with stabilizing a flipped out base, but interacts with the DNA as suggested from the previously published structure. One way to prove this result is to show that the DNA flips out in the absence of Met255. Notably. the previously published structure appears not to have the TEV site . Another possibility is that the true linker would also stabilize the flipped out position as the linker does contain some hydrophobic residues (as suggested from 9aan model), but the lack of sequence conservation in the linker would suggest not for such an important functional role. It should be detectable in the chemical shift experiments with the catalytic domain, but I was not able to locate that data. A final possibility is that stabilization of the flipped out base is not actually required, as seen for UDG glycosylase. However, the sugar pucker is sterically held in place in glycosylases, and it’s not clear that this would be the case in this NMR structure where the flipping out is near the end of the DNA with few protein contacts. If indeed the flipping is an artifact, then it is formally possible that the observed shifts in GTG binding and the nucleotide binding is also an indirect consequence.
2. Along these lines, in contrast to the strong NTP binding site, it appears that the weak NTP binding site is poorly conserved, suggesting it may be found only in specific systems. Is there other evidence that suggests a more universal role?
3. The authors state on line 87 that they present three structures in this publication. While the methods used to build these hybrid models are now better clarified in the main text, the distinction between the three structures remains potentially confusing for readers. Specifically, only one structure (9RTJ) is a conventional NMR structure determined entirely from experimental NMR restraints, whereas the other two structures are hybrid models built by integrating NMR restraints with crystal. We suggest the authors more explicitly differentiate the hybrid models from the NMR throughout the text..
4. If you make one mutation on two salt bridges, you can only say that the related salt bridge is not critical, not both, as implied in line 334
5. The authors state that the primary rationale for domain closure is the Watson Crick basepairing. However, they do not mention if the GTG sequence is also required. Their model suggests an extensive interface, providing additional check that the GTG is present.
6. The chemical shift blots are difficult to read. There’s no tick marks making it almost challenging to figure out which residue is showing chemical shifts.
7. Please state actual K351 mutation on line 318. Please also state which residue was deleted in linker in line 324.

8. Please include pdb nomenclature in the supplemental figures.

Reviewer #3

(Remarks to the Author)
The responses to the reviews are thorough and address all concerns adequately.

Reviewer #4

(Remarks to the Author)

Open Access This Peer Review File is licensed under a Creative Commons Attribution 4.0 International License, which permits use, sharing, adaptation, distribution and reproduction in any medium or format, as long as you give appropriate credit to the original author(s) and the source, provide a link to the Creative Commons license, and indicate if changes were made.
In cases where reviewers are anonymous, credit should be given to 'Anonymous Referee' and the source.
The images or other third party material in this Peer Review File are included in the article's Creative Commons license, unless indicated otherwise in a credit line to the material. If material is not included in the article's Creative Commons license and your intended use is not permitted by statutory regulation or exceeds the permitted use, you will need to obtain permission directly from the copyright holder.
To view a copy of this license, visit https://creativecommons.org/licenses/by/4.0/

Point-by-point response to the reviewers' comments

REVIEWER COMMENTS

Reviewer #1 (Remarks to the Author):

I suggest that the authors address the following points to improve the clarity of the article:

1. The protein should be identified in the methods section by its Uniprot entry Q54324.

   Response: We added "(UniProt accession number Q54324)" in the methods section. See words in yellow in paragraph 3 of page 36.

2. The construct spanning the catalytic and the HBD domains is named "full-length" in many instances. This is incorrect and misleading because the large C-terminal domain is not present in the construct studied in this work. Hereafter, I will refer to this construct as "primase" (as the authors label it in Figure 1a).

   Response: We thank the reviewer for this suggestion. We agree and have therefore replaced "full-length primase" with "primase" throughout the manuscript.

3. The authors say that the two domains of the primase do not interact in the apo form, but in the crystal structure of the primase (PDB 3M1M, described in reference 21), the two domains do interact. This discrepancy should be clarified.

   Response: In the crystal structure of pRN1 primase (PDB: 3M1M), two hydrogen bonds can indeed be observed between the catalytic domain and the HBD. However, the interface between the two domains is minimal and does not involve a substantial buried surface area or extensive stabilizing interactions. In addition, NMR data indicate that the spectra of the isolated domains closely match those of the intact primase, suggesting that the catalytic domain and the HBD tumble independently in solution[1]. This observation supports a model in which the two domains are connected by a flexible linker and adopt an open and dynamic arrangement in the apo state. It is therefore possible that the hydrogen bonds observed in the crystal structure reflect a transient orientation that become stabilized by crystal packing, whereas in solution the domains sample multiple relative orientations.

4. The use of some atomic labels common in the NMR field, used in several of the figures showing NMR spectra, should be explained, so that non-NMR experts are not confused. For instance, I think that “T2Q7” in extended Figure 1c refers to the methyl of T2 in extended Figure 1c, but this is not obvious for the average reader.

   Response: We have added an explanation of the atomic labeling convention used in the NMR spectra to clarify the notation for readers who may not be familiar with standard NMR nomenclature.

   Specifically, in the legend of Fig. 1e (Page 10), we added the following sentence:

   “Q denotes a pseudo-atom representing three equivalent protons; for example, K340QZ refers to the three equivalent protons attached to the NZ atom of K340.”

   In the legend of Supplementary Fig. 1c, we added:

   “Q denotes a pseudo-atom representing two or three equivalent protons; for example, T2Q7 refers to the methyl group of T2, G253QA the glycine alpha protons and V354QG2 the gamma2 methyl of V354.”

5. I think it would be useful to follow the IUPAQ recommendation of naming the amino acids by the three-letter code in the text (not in the figures) so that there is no possible confusion with the same single letters used for nucleotides.

   Response: We thank the reviewer for this helpful suggestion and agree that using three-letter amino acid codes can help avoid potential ambiguity. In the present manuscript, however, nucleotide residues are numbered from 1 to 9, whereas protein residues are numbered from 40 to 370, which clearly distinguishes DNA nucleotide from amino acid residues throughout the text. In addition, the one-letter amino acid notation is also widely used in structural biology.

   Converting all amino acid names from one-letter to three-letter codes throughout the manuscript would require extensive modifications in both text and figures without substantially improving clarity in the current context. For these reasons, we have retained the one-letter amino acid notation in this manuscript. Nevertheless, we appreciate the reviewer’s suggestion and will consider adopting the three-letter notation more systematically in future work to further improve clarity.

6. Lines 172 and 1185: “Linker” should be “linker”.

   Response: We have changed “Linker” to “linker” in these two positions.

7. Line 172: the GHM residues are not TEV cleavage ones (only the Gly is), they are remaining non-native residues after TEV cleavage.

   Response: The wording has been corrected in the revised manuscript. In the legend of Fig. 1a, it now reads:

   "*three additional non-native residues (GHM, residues 253-255) remaining after TEV cleavage*".

8. Line: "represent" should be "representative".

   Response: We have changed "represent" to "representative" in the legend of Fig. 1g.

9. Line 247 and line 1102: open and closed states are mentioned along the text, but here a "fully" open state is mentioned. Is this a different state from the open one?

   Response: The term "fully open state" was originally intended to refer to the same conformation as the DNA_open state. To avoid ambiguity, we have now replaced "fully open state" with "open state" in these two lines so that a consistent terminology is used.

10. Line 1103: same as above, with "fully" closed state.

    Response: We have now replaced "fully closed state" with "closed state" in this line.

11. The crystal structure of the primase in the apo state does not show magnesium cations. The experimental data supporting the three cations that are actually bound to the primase (three in the 2bp_closed) should be stated, as this information is used in the modelling.

    Response: The placement of the three magnesium ions in the 2bp_closed model is supported by both our NMR data and previously reported structures.

    Our NMR data show that shortly after dinucleotide formation from the 2bp_closed state, the triphosphate group of the dinucleotide, which is derived from the ATP triphosphate, remains bound at the NTP^S site of the HBD (Supplementary Fig. 7c). This observation indicates that in the 2bp_close state the ATP triphosphate is also bound at the NTP^S site of the HBD. Because NTP binding at this site requires coordinating with Mg^{2+}, as illustrated in the newly added Supplementary Fig. 9f, this implies that a magnesium ion is present a the NTP^S site to coordinate the ATP triphosphate.

    In addition, the two-metal-ion catalytic mechanism is conserved in archaeal-eukaryotic primases. For example, crystal structures of the catalytic domain of MsCAPP primase in complex with two base pairs (PDB: 7P9J and 7QAZ) reveal two catalytic metal ions at the

active site. Notably, the structure of PDB: 7QAZ also shows a third metal ion coordinated with the triphosphate group of the initiating NTP, even in the absence of the ancillary HBD domain.

Together, these observations support a model in which one magnesium ion coordinates the ATP triphosphate at the NTP^S site of the HBD, while two catalytic magnesium ions are present in the active site. Accordingly, three magnesium ions were included in the 2bp_closed model of pRN1 primase. To clarify this point, we have added an explanation in the results section (page 22, paragraph 1, highlighted in yellow) and included a new panel (Supplementary Fig. 9f) illustrating the Mg^{2+}-dependent binding of NTP at the NTP^S site.

12. Lines 282-284: Some words are missing in this sentence to make full sense.

Response: We have revised these sentences to improve clarity. It now reads as follows (highlighted in yellow in paragraph 2 on page 15):

"In contrast, when we generated independent models of the primase-DNA^{CT}-2ATP complex in the absence of a base pair using different structural approaches (Fig. 3h, and Supplementary Fig. 6a), the interdomain linker was too short to allow binding between the two domains in any model."

13. Line 310: "wide" should be "wild", and "mutants" should be "mutant".

Response: We have corrected "wide" to "wild" and "mutants" to "mutant" in legend Fig. 3i.

14. Line 343: "Fig. 5e" should be "Fig 7e".

Response: We have corrected "Fig. 5e" to "Fig. 7e".

15. Explain the colors of the spheres used to represent the different cations in Figure 4.

Response: We have clarified the color coding of the cations in the legend of Fig. 4a.

16. Line 501: Does the abundance refer to the cytoplasm or the nucleoplasm?

Response: The review by Thomas W. Traut (1994) indicates that most measurements of nucleotide concentrations were performed using whole cells or tissues rather than isolated subcellular compartments[2]. Therefore, the reported values represent total cellular concentrations. The same study also notes that compartmentation between the cytoplasm and nucleus is generally not supported for rNTP and dNTP, suggesting that nucleotide pools tend to equilibrate between these two compartments.

Accordingly, we replaced the original sentence with the following revised version (highted in

yellow in paragraph 1 on page 29):

"Specific recognition of this motif occurs upon the binding of two rNTP molecules (rNTPs being 10 to 100 times more abundant than dNTPs in total cellular concentration)"

17. Line 457: "close" should be "closed".

Response: We have corrected "close" to "closed" in paragraph 2 on page 31.

18. Line 873: How do the CA and CB chemical shifts report on the formation of a hydrogen bond?

Response: Indeed, CA and CB chemical shifts do not directly report hydrogen bonds. In our analysis, slowly exchanging amide protons detected by ^{1}H-^{15}N H/D exchange experiments were used as the primary indicators of backbone hydrogen bonds. The CA and CB chemical shifts were used to infer secondary structure propensities, which helped validate the placement of hydrogen bonds consistent with α-helices or β-strands in the preliminary structure. We have revised the text to clarify this point (highlighted in yellow in paragraph 2 on page 40):

"TALOS+ provided dihedral angle constraints for the protein. Intramolecular hydrogen bonds were identified from ^{1}H-^{15}N hydrogen-deuterium exchange data and the preliminary structure, supported by secondary structure propensities inferred from CA and CB chemical shifts."

19. Line 944: the quality of these NMR spectra could be illustrated in an extended figure, or a reference could be provided if this kind of measurements have been shown before.

Response: We thank the reviewer for this helpful suggestion. The NMR spectra demonstrating that the dinucleotide product and unbound DNA resonance near -1 ppm due to signal overlap were previously reported and characterized in the Figure 3a and 3b of our earlier study[3]. To clarify this point, we have now added the appropriate citation in the manuscript.

20. Lines 976 and 977: the reported entries in the PDB-IHM could not be found, perhaps because they have not yet been deposited.

Response: The two structural models have been deposited in PDB-IHM. At the time of submission, the entries were placed on hold until publication, which is why they were not accessible. Following the reviewer's comment, we have contacted the PDB-IHM and requested that the entries be released. The accession codes 9AAN and 9AAM are now

publicly available.

21. In relation to the former issue in lines 976-977, the pdb files that are available in the submission are mislabeled: the "1bp" is the two-base pair model, and the "2bp" is the one-base pair model.

    Response: We have contacted the PDB-IHM to release our entries. The reviewer can now download the correctly labeled models directly from the PDB-IHM database or RCSB PDB database (rcsb.org) using the accession codes 9AAN ("1bp" model) and 9AAM ("2bp" model).

22. Line 1050: "ribonulceotide" should be "ribonucleotide".

    Response: We have corrected "ribonulceotide" to "ribonucleotide".

23. Lione 1086: "complexed" should be "complex"

    Response: We have corrected "complexed" to "complex".

24. The CSP plots are designated as "histograms", but they are not histograms, they are bar plots.

    Response: We have corrected "histograms" to "bar plots" throughout the figure legends and manuscript.

25. Explain the red spheres in extended Figure 4e.

    Response: The hot pink sphere in the pRN1 primase structure represents a zinc ion, whereas the hot pink spheres in the MsCAPP primase structure represent cobalt ions. We have revised the legend of Supplementary Figure 4e to clarify this.

26. In extended Figure 4, the label "DNA_open" is new and confusing. Shouldn´t it be "1bp_open"?

    Response: The DNA_open state is distinct from the 1bp_open state. In the DNA_open state, the pRN1 primase is bound only to the DNA template without any NTP bound. In contrast, the 1bp_open state refers to the complex in which the primase binds both the DNA template and NTPs, and one base-pair has formed in the HBD domain. In both states, the catalytic and HBD domains adopt an open conformation. To avoid confusion, we have clarified the definition of the DNA_open state in the legend of Supplementary Fig. 4b:
    "The DNA_open state represents the primase bound only to the DNA template without NTPs, in which the two domains adopt an open conformation."

27. Line 1105: "conformaitonal" should be "conformational".

Response: We have corrected "conformaitonal" to "conformational".

28. Line 1105: I doubt they are "transition states" (high-energy, low-populated states). Perhaps the authors mean "intermediate states".

    Response: We agree that the term "transition state" is not appropriate in this context, as the states described here represent experimentally observed structural intermediates rather than high-energy transition states. We have therefore replaced "transition states" with "intermediate states" in the revised manuscript.

29. In extended figure 7e, the label "wt" would be better changed to "NTP".

    Response: We have changed the label "wild-type" to "NTP" in Supplementary Fig. 7e.

30. Line 1162: Is the R311 resonance from the backbone or the side chain NH group?

    Response: The R311 resonance arises from the backbone amide. To clarify this, we have changed the label from "R311" to "R311H" in Supplementary Fig. 7e, and revised the corresponding figure legend accordingly.

31. Line 1164: "the" should come after "at".

    Response: We have put "the" after "at".

32. Line 1166: It is unclear what "wild type" means when used for NTPs. I think it means non-chemically modified (and does not refer to cognate). Please clarify.

    Response: Here, we tried to use "wild type" to indicate a non-chemically modified NTP. To avoid confusion, we have removed this term and replaced "wild type NTP" with simply "NTP" in the figure legend.

33. Line 1176: "Align" should be "Aligned".

    Response: We have corrected "Align" to "Aligned".

34. Line 453 and the extended figure 8b: in the scheme, a pi-pi network is presented as being the same in both the pRN1 primase model and the MsCAPP crystal structure, with G3 in pRN1 stacking with both T2 and the ATP at the I-site. This seems to be the case, inspecting the corresponding pdb file of the pRN1 model, but not the pdb of MsCAPP. In the structure of MsCAPP, the corresponding base of the template is stacked with the other bases of the DNA chain, but not with the isolated nucleotides. If Therefore, the scheme does not properly represent the extent of similarity. If the authors think it does, it should be explained what the precise criterion is used to identify which base is stacked to which other base, and explain to

what extent the pi-pi network is similar in both models.

Response: We thank the reviewer for carefully examing the structural models and for raising this important point. In our pRN1 primase model, the base of G3 is positioned between T2 of the DNA template and the ATP located at the I-site of the catalytic domain (Supplementary Fig. 8b), which is also simultaneously coordinated at the NTP^{S} binding site of the HBD (Supplementary Fig. 7c). In this arrangement, G3 forms a π-π stacking network with both T2 and ATP. This configuration is supported by experimental observations from our previous NMR structure of HBD-DNA^{CT}-2ATP (PDB: 6GVT), which shows that the ATP triphosphate is tightly coordinated by residues within the HBD even before ATP forms base pair with T2. The same structure also reveals hydrogen-bond interactions between the G3 base, HBD residues, and the ATP triphosphate. These interactions position G3 close to ATP, allowing it to stack with both T2 and the ATP base after two base pairs form and T2 pairs with ATP. Thus, the stacking of G3 between T2 and the NTP in our model is supported by specific experimental restraints.

In contrast, the available crystal structure of MsCAPP contains only the catalytic domain and lacks the ancillary HBD domain. Because the HBD is absent, the triphosphate of the first NTP at the I-site is less constrained and the corresponding template base (A8) cannot form additional stabilizing hydrogen-bond interactions with the HBD or with the triphosphate of the first NTP (GTP in this case). As a result, the stacking network differs from that observed in the pRN1 model: in the MsCAPP structure, A8 primarily stacks with the adjacent DNA base (C7) rather than with the isolated nucleotide. Therefore, the different π-π stacking arrangements observed in the two complexes arise from the presence of the HBD in pRN1 primase, which stabilizes the nucleotide and enables additional hydrogen-bond interactions that are not possible in the MsCAPP structure.

To more accurately reflect these structural difference, we have revised the schematic in Supplementary Fig. 8b so that it better represents the stacking interactions observed in the deposited MsCAPP structure (PDB: 7P9J). We have also clarified this point in the revised manuscript text (highlighted in yellow in paragraph 2 on page 20).

35. In relation to the former issue, if the different stacking is maintained, it should be discussed whether it is supported by specific experimental restraints or is driven by the force field used to build or refine the model.

Response: See aboved.

36. In extended Figure 8b, the numbering of the nucleotide sequence of the MsCAPP is different from the numbering in the pdb file 7P9J. Please, clarify

Response: We thank the reivewer for pointing this out. In the original version of Supplementary Fig. 8b, we adjusted the nucleotide numbering of the MsCAPP template to facilitate comparison with the nucleotide numbering used throughout our manuscript. However, we agree that this may cause confusion when comparing the figure with the deposited structure.

To avoid ambiguity, we have now reverted the nucleotide numbering in Supplementary Fig. 8b so that it matches the numbering used in the deposited structure (PDB: 7P9J). This change ensures consistency between the figure and the PDB file.

Reviewer #2 (Remarks to the Author):

260204primase_review

The primase work by Wu and colleagues carried out NMR work that indicated a novel, unexpected mechanism for initiation of priming, a major question in the field. Their data was complex, with nucleotides binding in unexpected places and a flipped out base in the template strand right where priming should be occurring. The mechanisms revealed by this study are important and would be of interest to Nature Comm readers and the replication and repair fields.

The major concern is the paper's lack of clarity in correlating the structural models (CIFs/PDBs) with the experimental NMR data (e.g., chemical shifts, NOEs, RDCs) used to determine them. The authors appear to shift between discussing determined structures and discussing unassigned or dynamically averaged NMR data, creating significant ambiguity. This makes it challenging for non-specialists in solution NMR (including many structural biologists) to critically evaluate the mechanisms.

Response: We thank the reviewer for this important comment. We agree that the previous version did not clearly correlate the structural models with the experimental NMR data used to generate them. To clarify the relationship between the structural models and the underlying

experimental data, we now explicitly indicate which features were transferred from closely related NMR structures on the basis of highly similar chemical shifts and which were modeled using previously determined crystal structures because direct NMR restraints were unavailable.

Specifically, for the primase–DNA^{CT}–1bp model, we now state that the restraints for the HBD, linker, ATP, the dGTP triphosphate, and DNA template residues T2-A9 were taken from the NMR structure of the HBD–DNA^{CT}–ATP–dGTP complex, supported by the nearly identical chemical shifts observed for these components in the two complexes. We also clarify that PRE data guided placement of the dGTP-C1 base pair in the I-site, whereas the catalytic domain was modeled using the crystal structure of the pRN1 primase because many residues in this domain, particularly near the active site, are highly dynamic and therefore not observable in the NMR spectra. The geometry of the dGTP-C1 base pair, excluding the dGTP triphosphate, was modeled based on the corresponding base pair in the MsCAPP structure. These clarifications have been incorporated into the Results text in paragraph 3 on page 14, and are highlighted in yellow.

Similarly, for the primase–DNA^{CT}–2bp model, we now clarify that restraints for the HBD and DNA template residues G3-A9 were taken from the HBD–DNA^{CT}–ATP–dGTP NMR structure, again supported by the highly similar chemical shifts observed in the two complexes. The ATP triphosphate was positioned using distance restraints derived from solid-state NMR spectra of the primase–DNA^{CT}–pNHppApH complex, whereas the catalytic domain was modeled using the pRN1 crystal structure. The geometries of the dGTP-C1 and ATP-T2 base pairs were modeled based on the corresponding I-site and E-site interactions observed in the MsCAPP structure. These clarifications have been incorporated into the Results text in paragraph 3 on page 19, and are highlighted in yellow.

Comments

1. For readers to immediately assess the significance of this work, please add the organism from which the primase is derived in the abstract and in the Fig. 1 legend. Along the same lines, it would be helpful to add for the general audience that the PRN1 plasmid provides a minimalist primase system. Use of an extremophile model enables many structural insights, but it should be mentioned.

Response: We thank the reviewer for this helpful suggestion. We have revised the abstract to explicitly state that the primase studied here is encoded by the pRN1 plasmid fro the

hyperthermophilic archaeon *Sulfolobus islandicus*. We also added a brief explanation that the pRN1 plasmid provides a minimalist primase system, which makes it particularly suitable for mechanistic and structural investigation. In addition, the organism source has also been clarified in the Fig. 1 legend as suggested.

2. The authors refer to 3 structures provided by this work, but only two CIFs are located. HBD-DNA-ATP-dGTP seemed to be missing. Figuring out which structure was which was confusing. Adding the assigned pdb deposition ID for structures determined by this paper to figures, including Supplementary figures and supplementary tables, would improve clarity from which coordinates the figures are derived.

Response: We apologize for the confusion. We have requested that the PDB and PDB-IHM entries be released so that reviewers can directly access and download the coordinates from the database.

To improve clarity, we have also added the corresponding PDB or PDB-IHM accession IDs to all relevant figures, including main figures, Supplementary Figures, and Supplementary Tables, so that it is immediately clear which structural coordinates were used to generate each figure.

In addition, to further avoid ambiguity, we have implemented consistent labeling throughout the manuscript and figures:

- Structures determined in this study are labeled “this study”
- Structures determined in our previous work (Julien et al. 2019) are labeled “previous”
- Structures determined by other groups and previously published are labeled “published”

3. There appears to be NMR data where the structures were not determined. How feasible would it be to determine the entire structure, as small changes may be propagated outside of the binding site?

Response: Determining full NMR structures for the intermediate states during dinucleotide formation is not feasible due to the rapid kinetics and high dynamics of the system. All the catalytic steps occurs within ~1 s, making the intermediates intrinsically transient and difficult to stabilize for structure determination.

Although we prepared several NMR complexes using different NTPs and NTP analogues to capture reaction intermediates, these complexes remain conformationally heterogeneous and do not provide sufficient restraints for full NMR structure calculations. In particular, many residues in the catalytic domain, especially those located in the active site, are already highly dynamic in the apo protein and are not observable in the spectra (Supplementary Fig. 4a). Upon substrate binding, additional residues broaden beyond detection, further limiting the number of detectable signals.

This behavior likely arises from the long flexible linker conneting the catalytic domain and the HBD, which allows substantial interdomain motion. Consistent with this dynamic nature, no structure directly determined from experimental data that captures the closed conformation of primase during dinucleotide formation has been reported, despite many recent cryo-EM studies.

Instead, we used the available NMR restraints to construct two structural models representing key intermediates. The primase-DNA^{CT}-1bp model illustrates a transient closed conformation in which the two domains cooperatively recognize the first base pair, a state that is too unstable to be captured using conventional structural approaches. The primase-DNA^{CT}-2bp model represents a more stable closed state where two base pairs are coordinated, positioning the substrates for rapid dinucleotide formation.

Together, these models integrate the available experimental restraints and provide a mechanistic framework for understanding how primase transitions from an initial transient recognition state to a catalytically competent configuration.

4. Figure 1. It was difficult to reconcile the HBD construct shown in Fig. 1 with the cif file of the full-length protein provided. If the cif file had only the HBD construct, then it would make sense. In the methods, it seems that the authors collected NMR data on both the truncated construct and the full-length. Please clarify or provide individual structures for each independent data set, if the truncated was collected as truncation could alter conformational dynamics in the protein. Throughout it seemed that the authors shifted easily between the two, making it difficult to follow.

Response: The NMR structure in Figure 1. corresponds to the isolated HBD construct. Unfortunately, this structure file was inadvertently omitted from the materials provided for

review. We apologize for this omission and we now include the HBD structure file in the revised manuscript.

5. Fig 1 Metal spheres are not defined at any point The authors have not described that they removed sections of the DNA bases. For the former, it was not until I looked at the cif files directly that I realized.

Response: In Fig. 1, the pink spheres represent magnesium ions, and this has now been clearly indicated in the figure legend.

In addition, nucleotide C8 and A9 of the DNA template were omitted from the structural representation in the figure for clarity. These nucleotides are highly dynamic and do not form stable interactions with the protein in the complex. Their omission was intended to improve visualization of the protein-DNA interactions at the binding interface. We have clarified this point in the figure legend in the revised manuscript.

6. Depth cueing in Fig 1 j makes it look like the lysines are coordinating with the metal.

Response: We have adjusted the viewing angle of the structure in Fig. 1j so that the lysine residues are clearly positioned farther away from the metal ion.

7. The authors do not make it clear that the structure described in Fig. 2 was previously published in Cell. What is new?

Response: We have now clarified in both Fig. 2 and the manuscript text which structure was previously reported and which is newly determined in this study. The HBD-DNA^{CT}-2ATP structure was previously reported in Cell (PDB: 6GVT), whereas the HBD-DNA^{CT}-ATP-dGTP structure represents a new structure determined in this study (PDB: 9RTJ). These labels have been added to the figure and corresponding description in the manuscript to avoid confusion.

8. To know the generality of the findings,an MSA this archaeal model system compared to the human, would show the conservation of key residues. The authors have an MSA with other archaeal plasmid-based systems that could be used to see close ortholog conservation. It would be helpful for readers to denote any residues mentioned in the text or regions, such as domains and the linker.

Response: To address the reviewer's suggestion regarding the generality of our findings, we performed a multiple sequence alignment (MSA) comparing pRN1 primase with MsCAPP, human primase (Pri1), and human PrimPol (Response Fig. 1). We also generated a structure-

based alignment highlighting the conserved catalytic motifs I–III (Response Fig. 2). The alignment shows limited overall sequence conservation between archaeal and human primases, consistent with previous studies reporting that members of the archaeal–eukaryotic primase (AEP) superfamily are highly divergent in sequence across kingdoms while retaining conserved catalytic functions and structural organization[4].

AEP superfamily contain a Prim-Pol catalytic domain together with an ancillary domain involved in primer initiation[5]. Despite sequence divergence, their catalytic cores share a conserved architecture consisting of an N-terminal α/β fold and a C-terminal RRM-like fold that harbor the conserved catalytic motifs I–III: motif I (DhD/E), motif II (sxH), and motif III (hD/E). The acidic residues in motifs I and III coordinate two catalytic metal ions, while the conserved histidine in motif II contributes to nucleotide binding.

Because pRN1 primase, MsCAPP, human primase, and human PrimPol originate from evolutionarily distant systems, their primary sequences and overall structures are highly divergent. As a result, the MSA primarily highlights conservation of the catalytic motifs I-III rather than extensive residue-level similarity. These features have already been well documented in previous studies of the AEP superfamily[4-6]. For this reason, we include the alignments in the response to illustrate motif conservation but have not incorporated them into the revised manuscript. Instead, the manuscript presents an MSA of archaeal plasmid-based primases to demonstrate close ortholog conservation.

To clarify this point in the manuscript, we have modified the following sentence in the Discussion (paragraph 2, page 35, highlighted in yellow):
"DNA primases universally contain at least a catalytic domain and an ancillary domain. Although primases, even within the archaeal–eukaryotic primase superfamily, are highly divergent in sequence across kingdoms, these domains function cooperatively to ensure precise dinucleotide formation—the first step in primer synthesis."

Despite the limited sequence conservation within the AEP superfamily, both the catalytic chemistry and the structural principle underlying primer initiation appear to be conserved. Previous studies have shown that the initiating nucleotide is stabilized by an ancillary helical module associated with the catalytic core in pRN1 primase[7], human primase[8], and human PrimPol[9]. Our structure further reveals how the pRN1 helix-bundle domain (HBD) prepares the template and nucleotides for dinucleotide formation. The conserved domain organization

and catalytic architecture observed across archaeal plasmid primases, eukaryotic primase, and PrimPol therefore support a broadly conserved mechanism within the AEP superfamily.

Finally, to facilitate interpretation by readers, the MSA with other archaeal plasmid-based systems in Supplementary Fig. 10 has been annotated to highlight key residues mentioned in the text, as well as the domains and linker region.

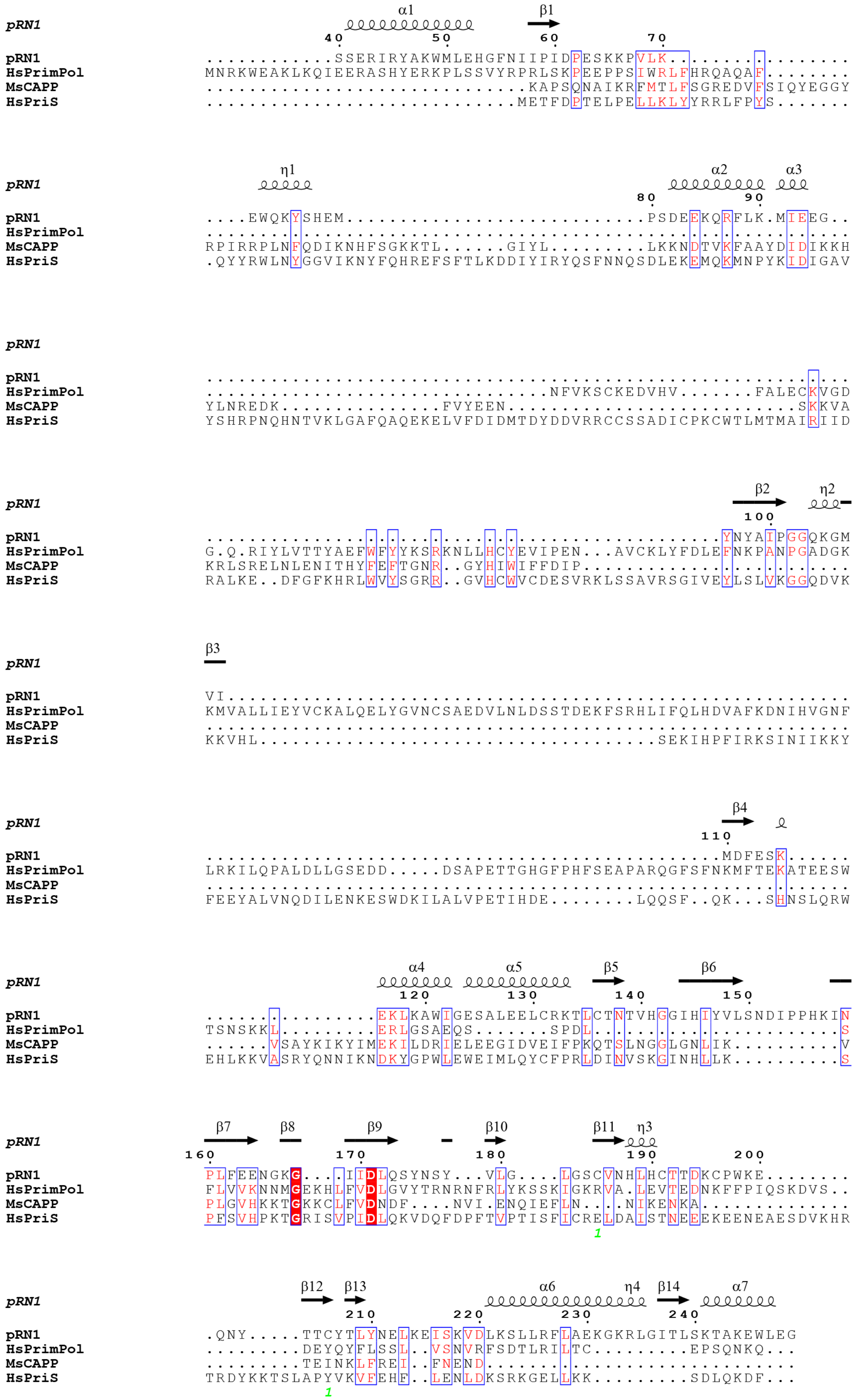
pRN1
α1 β1
40 50 60 70
pRN1 ............SSERIRYAKWMLEHGFNIIPIDPESKKPVLK.................
HsPrimPol MNRKWEAKLKQIEERASHYERKPLSSVYRPRLSKPEEPPSIWRLFHRQAQAF........
MsCAPP ..............................KAPSQNAIKRFMTLFSGREDVFSIQYEGGY
HsPriS .............................METFDPTELPELLKLYYRRLFPYS.......
pRN1
η1 α2 α3
80 90
pRN1 ....EWQKYSHEM............................PSDEEKQRFLK.MIEEG..
HsPrimPol ....
MsCAPP RPIRRPLNFQDIKNHFSGKKTL......GIYL.........LKKNDTVKFAAYDIDIKKH
HsPriS .QYYRWLNYGGVIKNYFQHREFSFTLKDDIYIRYQSFNNQSDLEKEMQKMNPYKIDIGAV
pRN1
pRN1 ....
HsPrimPol ................................NFVKSCKEDVHV.......FALECKVGD
MsCAPP YLNREDK...............FVYEEN...........................SKKVA
HsPriS YSHRPNQHNTVKLGAFQAQEKELVFDIDMTDYDDVRRCCSSADICPKCWTLMTMAIRIID
pRN1
β2 η2
100
pRN1 ....YNYAIPGGQKGM
HsPrimPol G.Q.RIYLVTTYAEFWFYYKSRKNLLHCYEVIPEN...AVCKLYFDLEFNKPANPGADGK
MsCAPP KRLSRELNLENITHYFEFTGNR..GYHIWIFFDIP.........................
HsPriS RALKE..DFGFKHRLWVYSGRR..GVHCWVCDESVRKLSSAVRSGIVEYLSLVKGGQDVK
pRN1
β3
pRN1 VI..
HsPrimPol KMVALLIEYVCKALQELYGVNCSAEDVLNLDSSTDEKFSRHLIFQLHDVAFKDNIHVGNF
MsCAPP ....
HsPriS KKVHL.....................................SEKIHPFIRKSINIIKKY
pRN1
β4
110
pRN1 ....MDFESK......
HsPrimPol LRKILQPALDLLGSEDD.....DSAPETTGHGFPHFSEAPARQGFSFNKMFTEKATEESW
MsCAPP ....
HsPriS FEEYALVNQDILENKESWDKILALVPETIHDE........LQQSF..QK...SHNSLQRW
pRN1
α4 α5 β5 β6
120 130 140 150
pRN1 ................EKLKAWIGESALEELCRKTLCTNTVHGGIHIYVLSNDIPPHKIN
HsPrimPol TSNSKKL.........ERLGSAEQS.......SPDL.......................S
MsCAPP ......VSAYKIKYIMEKILDRIEEEGIDVEIFPKQTSLNGGLGNLIK...........V
HsPriS EHLKKVASRYQNNIKNDKYGPWLEWEIMLQYCFPRLDINVSKGINHLLK..........S
pRN1
β7 β8 β9 β10 β11 η3
160 170 180 190 200
pRN1 PLFEENGKG....IIDLQSYNSY...VLG....LGSCVNHLHCTTDKCPWKE........
HsPrimPol FLVVKNNMGEKHLFVDLGVYTRNRNFRLYKSSKIGKRVA.LEVTEDNKFFPIQSKDVS..
MsCAPP PLGVHKKTGKKCLFVDNDF....NVI.ENQIEFLN...NIKENKA...............
HsPriS PFSVHPKTGRISVPIDLQKVDQFDPFTVPTISFICRELDAISTNEEKEENEAESDVKHR
1
pRN1
β12 β13 α6 η4 β14 α7
210 220 230 240
pRN1 .QNY.....TTCYTLYNELKEISKVDLKSLLRFLAEKGKRLGITLSKTAKEWLEG
HsPrimPol .........DEYQYFLSSL..VSNVRFSDTLRILTC.........EPSQNKQ...
MsCAPP .........TEINKLFREI..FNEND..............................
HsPriS TRDYKKTSLAPYYKVFEHF..LENLDKSRKGELLKK.........SDLQKDF...
1

Response Fig. 1 | Multiple sequence alignment of pRN1 primase, human PrimPol (HsPrimPol), MsCAPP, and the catalytic small subunit of human primase (HsPriS).

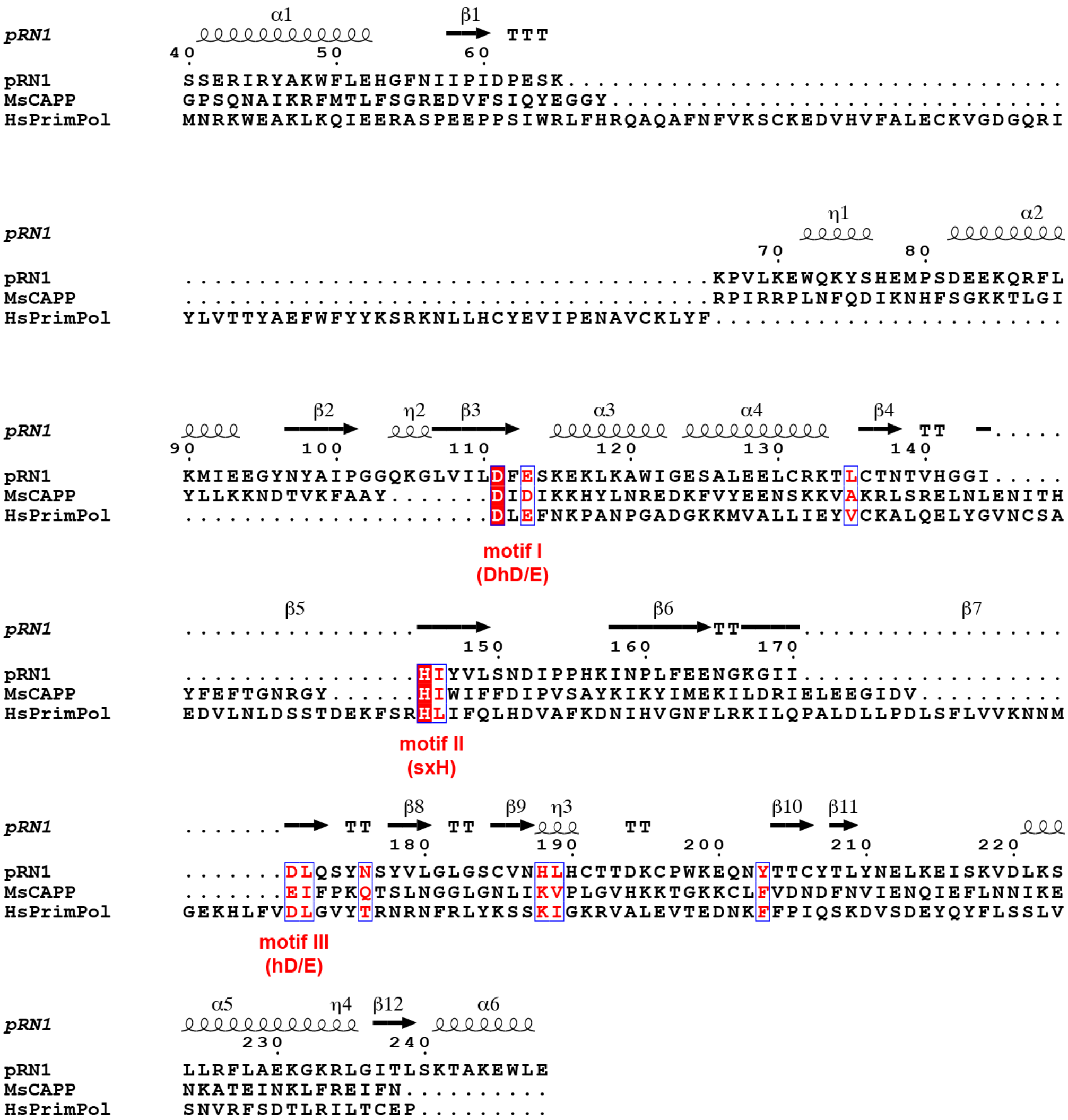

Response Fig. 2 | Structure-based multiple sequence alignment of pRN1 primase, MsCAPP, and human PrimPol (HsPrimPol).

9. The reported 5 Å phosphodiester-phosphodiester distance in the flipped-out T2 structure, when modeled with an expected C2'-endo sugar pucker, is structurally unusual and potentially incompatible with standard nucleic acid geometry. Please provide the primary experimental evidence.

Response: We thank the reviewer for this helpful suggestion. To provide direct experimental evidence for the conformation of T2, we have added a new Supplementary Fig. 14 (showed bellow). The NMR data demonstrate that T2 adopts a *syn* glycosidic conformation and a C2’-

endo sugar pucker in the HBD-DNA^{CT}-ATP^{C}-dGTP^{C} complex. Specifically, the NOESY spectrum shows a strong H6-H1' NOE for T2, indicating a short distance between these protons and supporting a *syn* glycosidic orientation. In addition, the TOCSY spectrum shows a strong H1'-H2' cross-peak, consistent with a large $^{3}J_{H1'H2'}$ coupling constant characteristic of a C2'-endo sugar pucker. These experimental observations are now presented in Supplementary Fig. 14 and described in the Methods section of the revised manuscript (paragraph 3, page 40, highlighted in yellow).

The unusually short phosphodiester-phosphodiester distance observed for the flipped-out T2 nucleotide is stabilized by extensive interactions with the protein and neighboring bases. In the structure, C1, T2 and G3 of the DNA template are tightly bound by the HBD through hydrogen bonding and stacking interactions (Fig. 1k,l). Notably, T2 is sandwiched between the linker region and residue K351, which stabilizes its flipped-out conformation and allows accommodation of the short phosphodiester-phosphodiester distance. In addition, C1 forms a stable stacking interaction with G3, supported by strong NOE cross-peak between C1H1' and G3H1' (Fig. 1d). This stable C1-G3 stacking further constrains the local backbone geometry and favors the compact arrangement around T2. Although the resulting phosphodiester-phosphodiester distance is shorter than typically observed in canonical nucleic acid structures, it is stabilized by the surrounding protein-DNA interactions and base stacking within the complex.

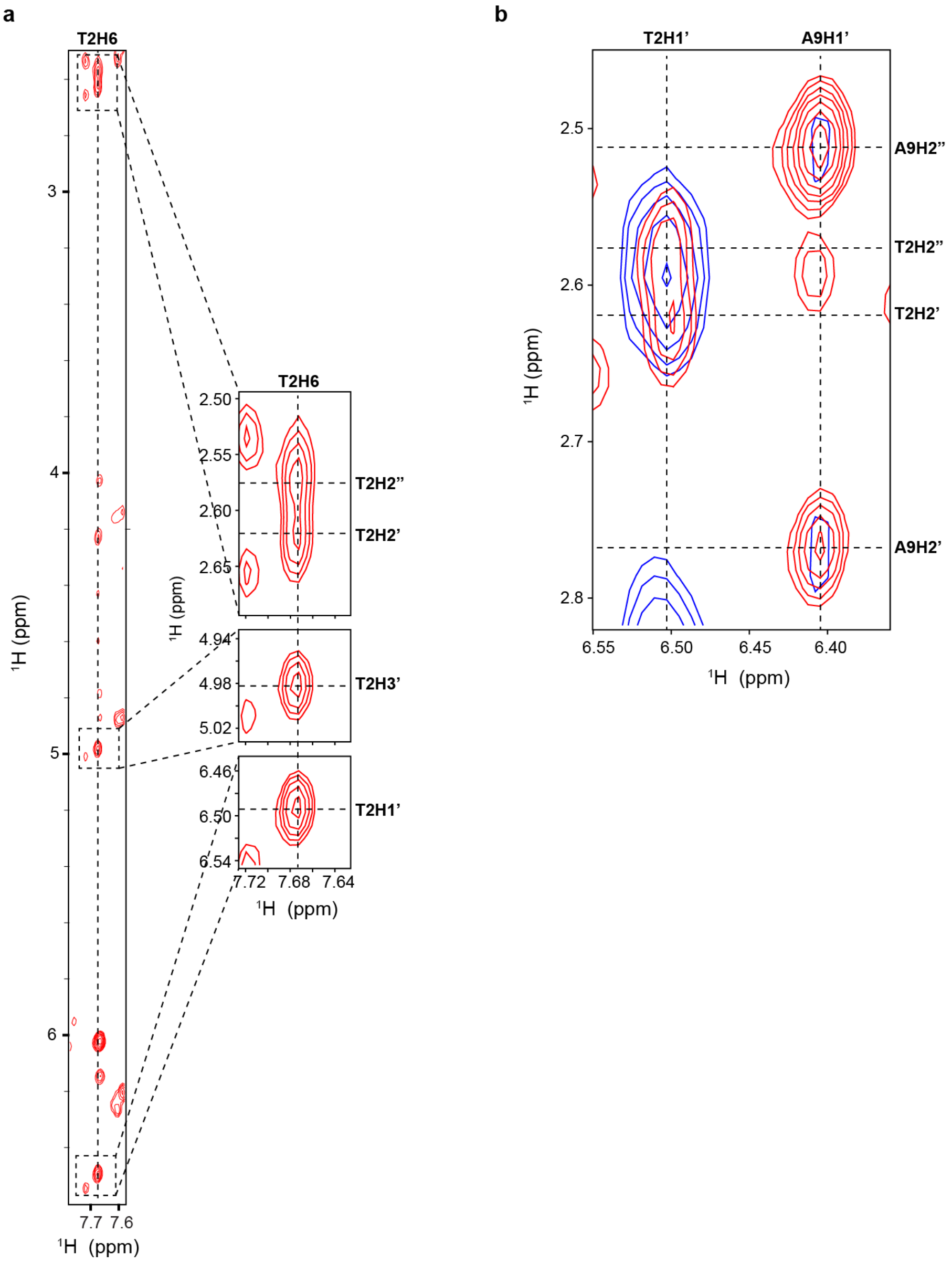

Supplementary Fig. 14 | Glycosidic conformation and sugar pucker of T2. a, Selected regions of the 2D F1fF2f NOESY spectrum of the HBD-DNA^{CT}-ATP^{C}-dGTP^{C} complex. The left panel shows intraresidue NOE cross-peaks between T2 H6 and the ribose protons of T2. The right panel zooms in and highlights the NOEs between T2 H6 and H1’, H2’, H2”, and H3’. b, Selected regions of overlaid the 2D ^{1}H-^{1}H TOCSY (blue) and 2D F1fF2f NOESY (red) spectra of the HBD-DNA^{CT}-ATP^{C}-dGTP^{C} complex. Cross-peaks between H1’ and H2’/H2” are shown for T2, with the corresponding signals for A9 included as a control.

10. The authors report that they mutated the linker residues to remove hydrophobic, yet they chose lysine, known in protein structures for the hydrophobility of the aliphatic chain. Their

choice of mutation did alter beta branching and electron dispersion. The authors argue the biological significance of the flipped out T2. Mutation of the linker partly addressed it, but a more precise mutation (e.g. K351) may be more selective in its effect.

Response: We thank the reviewer for this insightful comment. Our previous NMR structure of the HBD–DNA^{CT}–2ATP complex (PDB: 6GVT) showed that linker residues V257–F260 form a short α-helix connecting the catalytic domain and the HBD. Our current NMR structure of the HBD–DNA^{CT}–ATP–dGTP complex (PDB: 9RTJ) reveals that during the early stage of initiation, following formation of the first C1–dGTP base pair, this helical structure unfolds and the template nucleotide T2 flips out to interact with residues in the linker region. Our structural model of the primase–DNA^{CT}–2bp complex (PDB-IHM: 9AAM) further shows that once the second base pair forms and T2 pairs with ATP, T2 no longer contacts the linker residues. At this stage, the linker refolds into a helical conformation, effectively shortening the linker and enabling the catalytic domain and HBD to adopt a more compact closed conformation required for dinucleotide formation.

Based on these structural observations, the linker mutations were designed not only to disrupt the hydrophobic interaction with the flipped T2, but also to test the functional importance of the helix–coil transition of the linker during dinucleotide synthesis. We agree with the reviewer that substitutions in this region may affect multiple structural features, including β-branching and side-chain dispersion interactions that contribute to hydrophobic packing. Importantly, these mutations are also expected to perturb the ability of this region to transiently form an α-helix, which appears to be central to the conformational rearrangement observed during initiation.

We appreciate the reviewer's suggestion that a more targeted mutation, such as K351, could provide a more selective perturbation. Our previous study demonstrated that the K351A mutation completely abolishes dinucleotide formation by disrupting both DNA and NTP binding[1]. Therefore, this mutation is well suited to probe the functional role of the flipped T2 during dinucleotide formation. We have now clarified this point in the revised manuscript and cited the previous study accordingly.

Together, we have clarified in the revised manuscript that disrupting the interaction of the flipped T2 with either K351 in the HBD or the interdomain linker significantly impairs substrate preparation during dinucleotide formation (paragraph 3, page 15, highlighted in yellow).

11. In discussion of unexpected preference for pyrimidines, the other side (template) are purines. Could stabilization and stacking of the purines in the template strand be an important consideration?

Response: We thank the reviewer for this insightful suggestion. Stabilization and stacking interactions involving purine bases in the template strand likely contribute to the observed preference for pyrimidines at the N1 and N2 positions. Previous studies have shown that many primases preferentially initiate primer synthesis at sites where a purine base is located upstream of the initiation site, which can stabilize the initiating nucleotide through stacking interactions[6].

Consistent with this idea, our NMR structure of the HBD–DNA^{CT}–ATP–dGTP complex shows that the template guanine at position G3 (the 3′ neighbor of the N1 template base) stacks with both the template base C1 and the incoming dGTP (Fig. 1k). In this configuration, G3 primarily stacks with the incoming purine nucleotide, likely stabilizing the otherwise flexible nucleotide triphosphate during formation of the first base pair. This may also explain why free purine nucleotides readily form mismatched base pairs in the HBD complex (Fig. 5a–d).

However, stacking with template purines is not universally required. For example, in the structure of the p58C domain of human primase bound to an RNA-primed DNA template, the initiating GTP stacks against a histidine residue (H303) of the protein, while the upstream puine base stacks with the N1 template nucleotide[8].

Taken together, these observations suggest that stacking interactions within the template strand can contribute to stabilization of the initiation complex. Nevertheless, the primary reason for the preference for pyrimidines at the N1 and N2 positions is that they are less prone to form mismatched base pairs (Fig. 5a–d) and ensure incorporation of an incoming purine nucleotide during dinucleotide formation. Because the initiating nucleotide triphosphate is flexible and makes limited contacts with the protein, stabilization through stacking is critical. Pairing of template pyrimidines with incoming purine nucleotides enables favorable stacking interactions that stabilize the nascent base pair and promote efficient dinucleotide formation.

We have added this point in the Discussion part of the revised manuscript (paragraph 1, page 33, highlighted in yellow).

Reviewer #4 (Remarks to the Author):

The manuscript by Wu et al. describes a detailed structural and mechanistic study of primer initiation (dinucleotide synthesis) by the archaeo-eukaryotic primase pRN1. Through DNA-dependent de novo synthesis, primases perform the essential role of generating the RNA primers required to begin replicative DNA synthesis. Based primarily on NMR experiments, the authors propose a complete model for all steps of primer initiation. The supporting data appear to be of high quality, the manuscript is well written, and the figures are clear. However, in my opinion, a number of issues need to be addressed before the manuscript will be suitable for publication in Nature Communications.

1. The model presented is well supported but is not proven directly by experiment. Therefore, the title should be scaled back, perhaps to specifically state 'a model for' as opposed to 'molecular basis'. The tendency to over-interpret the fine details and overstate the certainty of the model is evident throughout the manuscript. A clear example is the passage starting on line 362: the discussion of a 4 degree shift in the orientation of a helix from a comparison of structural models represents an over-interpretation and is misleading because this is overly precise given the intrinsic uncertainty in the accuracy (and possibly the precision) of the structures.

Response: We thank the reviewer for this important comment. We agree that some aspects of the previous wording conveyed a level of precision and certainty that exceeded what is justified by the experimental data and the hybrid structural modeling approach. We have therefore revised the title to "Structural and Mechanistic Insights into Primer Synthesis Initiation by DNA Primase", which is better reflect the NMR-supported and model-guided nature of the work while preserving the central mechanistic insight provided by the

experimentally determined HBD structure and the NMR-guided models of the closed intermediates.

We have also revised the text (including the abstract) to use more cautions language when describing features derived from structural models. In particular, we removed the statement describing a “4 degree shift” in helix orientation, as this degree of precision is not warranted. Instead, we now describe the relatvie domain arrangement as largely similar between the two models and focus on the more robust difference in the interdomain linker region, which is further supported by chemical-shift changes in linker residues. These revisions are intended to better align the wording of the manuscript with the level of confidence supported by the data. The corresponding changes are highlighted in yellow in paragraph 1 on page 21 of the revised manuscript.

In this vein, the conclusions about key interactions are largely derived from secondary analysis, i.e. NMR structures calculated from the NMR data but not direct NMR evidence. This gives rise to a certain degree of uncertainty in the mechanistic insights and models presented. This is not a fundamental flaw, there are a number of useful insights and an interesting model presented. However, the does need to be clearly acknowledged in the Results and Discussion sections.

Response: We agree that the extent of this uncertainty should be stated more explicitly in the manuscript. To address this point, we have revised both the Results and Discussion to acknowledge the model-based nature of these mechanistic inferences more clearly. In the Results, we added the following statement: *“Thus, the 1bp_closed and 2bp_closed states are represented by NMR-guided structural models that are strongly supported by experimental data, although some mechanistic details remain model-based because full NMR structures of the entire complexes could not be directly determined.”* This sentence has been added to paragraph 1 on page 20 of the revised manuscript and is highlighted in yellow.

In the Discussion, we also softened the wording of the mechanistic summary to better reflect the level of confidence supported by the data. Specifically, we revised the sentence from *“Our structural NMR data reveal a sequential pathway by which the pRN1 primase assembles the DNA template and the first two NTPs for dinucleotide formation”* to *“Our structural and spectroscopic data support a sequential model for how pRN1 primase assembles the DNA template and the first two NTPs during dinucleotide formation.”* This

change has been made in paragraph 4 on page 28 of the revised manuscript and is highlighted in yellow.

2. The initiation mechanism described for pRN1 is claimed to be ‘highly likely conserved across all primases’ and ‘likely conserved among all known primases’. Such a broad generalization made with such a high degree of certainty seems unwarranted. These statements should be moderated. Also, it would be helpful if the authors clarified which features of their model that they believe to be shared by other primases, and by which other primases. Some features, such as recognition of a specific sequence and transient flipping of a specific nucleotide, may be shared by more closely related enzymes but may not be universal.
Response: We agree that the previous broad generalizations regarding the conservation of the pRN1 initiation mechanism across all primases were unwarranted. We have therefore moderated these statements to better reflect the available evidence.

Specifically, in the Abstract we replaced “*Given its essential role, this initiation mechanism is highly likely conserved across all primases*” with “*Given its essential role, this initiation mechanism is likely general among most know primases.*” Similarly, in the Introduction we replaced “*This mechanism is likely conserved among all know primases*” with “*This mechanism is likely general among most know primases*”.

Additionally, to address the reviewer’s request for clarification on which features of our model are shared by other primases and which are system-specific, we have added the following content in the Discussion section in paragraph 2 of page 36 (highlighted in yellow):

“Taken together, our pRN1 results support a broadly shared principle of primer initiation, in which the ancillary domain forms the first productive base pair with the second cognate NTP before this intermediate is handed over to the catalytic domain. In this view, the ancillary domain first engages the DNA template, organizes the initiating nucleotide, and stabilizes the first base-pairing event. After transfer to the catalytic domain and dinucleotide formation, the ancillary domain is proposed to continue stabilizing the 5’-triphosphate of the nascent primer during early extension, while the catalytic domain adds incoming nucleotides to the 3’-end. We suggest that this overall functional logic may be shared by many primases. By contrast, the precise structural details observed here, including GTG recognition and linker-mediated sensing of a flipped template base, are more likely to be system-specific.”

3. The use of NMR-based RNA priming assays would be significantly strengthened if results were compared to the significant literature of gel-based biochemical RNA priming assays.

Response: We agree that direct comparison with the established gel-based and HPLC-based biochemical literature is important for interpreting the NMR-based RNA priming assays. To address this point, we revised the manuscript to place our NMR-based results more explicitly in the context of previous biochemical priming studies.

Specifically, in paragraph 3 on page 15, we added the following text: "*In agreement with this model, our previous study showed that mutation of K351, which stacks with T2 and stabilizes its interaction with the linker (Fig. 1l), completely abolishes dinucleotide formation by disrupting both DNA and NTP binding. In the present study, we additionally introduced mutations in the interdomain linker (V257-F260) that disrupt both a-helical formation and hydrophobic interactions with T2, which significantly reduces primase activity (Fig. 3i). These findings are also consistent with previous studies using an HPLC-based primase assay, which showed that linker length is critical for the primase synthesis, as shortening the flexible linker by a single amino acid reduce primer activity to approximately 25% of the wild-type level*[10]*. The agreement with this independent biochemical assay supports the structural relevance of the effects observed in our NMR-based priming experiments. More broadly, the two approaches are complementary: HPLC-based assays define primer synthesis activity and product distribution, whereas the NMR-based assay enables direct monitoring of substrate binding, intermediate formation, and mechanistic transitions during dinucleotide synthesis at atomic resolution*."

In paragraph 2 on page 22, we modified the following sentence:
"*Many of these residues have previously been shown by mutagenesis, using conventional gel-based primase assays, to be essential for DNA-binding and enzyme activity*[7,11]*, providing an independent biochemical framework for interpreting our NMR-based priming assays.*"

Together, these revisions clarify that the trends observed in our NMR-based assays are consistent with those obtained using established gel-based and HPLC-based biochemical assays, thereby strengthening the reliability of the conclusions drawn from the NMR experiments. We also revised the text to emphasize the complementary strengths of these

methods. Whereas gel-based and HPLC-based assays provide robust measurements of primer synthesis activity and product distribution, the NMR-based assay enables direct monitoring of substrate binding, intermediate formation, and mechanistic transitions during dinucleotide synthesis. Together, these approaches provide a more complete picture of primer initiation.

4. While the clear focus of the study is the mechanism of primer initiation, the manuscript would be more impactful if the Discussion included speculation about primer elongation and termination, as introduced on line 38. Of particular interest, and following from the results described in the manuscript, are the roles played by the ancillary domains during these subsequent stages.

Response: We thank the reviewer for this helpful suggestion. We agree that the manuscript is strengthened by discussing how the mechanistic framework defined here for primer initiation may extend to primer elongation and termination, particularly with respect to the role of the ancillary domain. To address this point, we added a speculative paragraph to the Discussion (paragraph 2 on page 34, highlighted in yellow).

In the revised text, we propose that the ancillary domain may continue to function after dinucleotide formation by remaining associated with the 5′ end of the nascent primer, most likely through interaction with the 5′-triphosphate. We further suggest that this interaction could help stabilize the growing duplex during elongation, while the catalytic domain extends the 3′ end. In this framework, the ancillary domain may act not only as a processivity factor during early primer extension but also as a determinant of primer-length control by imposing a geometry-dependent limit as the duplex grows. We also note that previous biochemical work on pRN1 argues against a simple caliper model based solely on linker length, suggesting instead that primer termination reflects a more specific structural constraint intrinsic to the primase. Finally, we relate this model to human primase, in which the ancillary PriL C-terminal domain has been shown to regulate primer length and handoff to Polα, supporting the broader idea that ancillary domains can stabilize the 5′ end of the product during elongation and help position the completed primer for release or transfer after termination.

We think that these additions place our initiation model in a broader functional context and clarify how the ancillary domain may contribute not only to primer initiation, but also to elongation and termination.

Additional issues relating to specific statements in the text:

Lines 31-43: 'DNA primase' and 'DNA polymerase' should be plural not singular.

Response: We have corrected "DNA primase" and "DNA polymerase" to the plural forms, "DNA primases" and "DNA polymerases" in the revised manuscript.

Lines 59,69: These are strange uses of the term apo.

Response: We have revised the following sentences in the Introduction:

- "*In its apo form, the two domains do not interact with each other and remain in an open conformation, connected by a flexible interdomain linker.*"
  has been replaced with
  "*In the absence of bound DNA and nucleotides, the two domains do not interact with each other and remain in an open conformation, connected by a flexible interdomain linker.*"
- "*Several primase structures, including apo forms and complexes with single NTPs or chimeric DNA-RNA template-primers, have been reported.*"
  has been replaced with
  "*Several primase structures have been reported, including unbound forms lacking DNA and nucleotides, as well as complexes with a single NTP or with chimeric DNA-RNA template-primers.*"

Line 76: Please clarify if this statement specifically refers to MsCAPP. If this refers to all primases, the statement is incorrect.

Response: We have revised the sentence in the Introduction to clarify that it specifically referred to MsCAPP: "Furthermore, there is currently no structural information on how the catalytic and ancillary domains of MsCAPP coordinate during primer synthesis. Although that study concluded that the catalytic domain alone is sufficient for dinucleotide formation *in vitro*, both domains of archaeal-eukaryotic primases are required under physiological

conditions."

Line 102: This is a strange use of the term wild-type.

Response: We have replaced "wild-type NTPs" with "NTPs"

Line 221: 'changed' should be 'changes'

Response: We have replaced 'changed' with 'changes'.

Lines 258-259: The statement regarding closed conformations should be modified to acknowledge the two such structures of human primase that have been determined (He et al., Nature 2022 and Cordoba et al., J. Mol. Biol. 2023)

Response: We have revised the Results text to acknowledge these human primase structures while clarifying that the transient closed intermediate associated with formation and handover of the first base pair remains structurally uncharacterized.

Specifically, we replaced:

"The closed conformation between the catalytic and ancillary domains in all primases has remained uncharacterized due to its transient nature, making it difficult to capture using conventional methods such as NMR, X-ray crystallography, or cryo-EM."

with:

"Although recent studies have captured preinitiation and elongation-related states of human primase and proposed an initiation model for the priming complex, the transient closed intermediate associated with formation and handover of the first base pair remains structurally uncharacterized."

Lines 282-284: The meaning of this sentence is unclear and should be clarified.

Response: We have revised these sentences to improve clarity. It now reads as follows (highlighted in yellow in paragraph 2 on page 15):

"In contrast, when we generated independent models of the primase-DNA^{CT}-2ATP complex in the absence of a base pair using different structural approaches (Fig. 3h, and Supplementary Fig. 6a), the interdomain linker was too short to allow binding between the two domains in any model."

Lines 303-304: The optimal selection of a representative structure from an ensemble is the one structure that is closest to the mean of the ensemble.

Response: We thank the reviewer for this helpful comment. We agree that the conformer closest to the ensemble mean is often used as a representative structure in NMR studies. At

the same time, the lowest-energy conformer is also commonly used for graphical representation. In our manuscript, we chose the lowest-energy conformer for consistency across the figures. Importantly, the ensemble is tightly converging, with a backbone RMSD of 0.46 Å, so the lowest-energy conformer and the conformer closest to the ensemble mean are expected to be highly similar. Our structural interpretations are therefore based on the ensemble as a whole, and the choice of displayed representative conformer does not affect the conclusions.

Lines 385-389: This section is vague. Which residues were mutated? How is mutation of these residues believed to impair dinucleotide formation?

Response: In the revised manuscript, we now specify which residues were mutated, what interactions they make in the structural model, and why these interactions are expected to be important for dinucleotide formation.

Specifically, we mutated residues predicted by the model to stabilize substrate binding in the catalytic domain. These included Q73, K157, and N176, which interact with the DNA phosphate backbone; W72, N159, and Q173, which contact the ribose moieties of the two base pairs; K167, which contacts the ATP triphosphate; and T139, H141, H145, K195, and Y207, which coordinate the dGTP triphosphate. We then monitored dinucleotide formation by time-resolved ^{31}P 1D NMR spectroscopy using wild-type and mutant proteins. Mutations at these positions led to strong reductions in primase activity, consistent with the model in which these residues stabilize the DNA, the initiating nucleotides, and their triphosphates in the catalytically competent 2bp_closed state.

We have revised the Results accordingly to clarify both the mutational design and its mechanistic interpretation in paragraph 2 on page 22.

Line 387: ‘confirm’ should be scaled back to ‘are consistent with’

Response: We have scaled back ‘confirm’ to ‘are consistent with’.

Line 482: poorly understood structurally

Response: We have replaced ‘poorly understood’ with ‘poorly understood structurally’.

Lines 482-483: One recent model was proposed in Cordoba et al., J. Mol. Biol. 2023.

Response: We agree and have revised the Discussion to acknowledge the recent model proposed by Córdoba et al. (*J. Mol. Biol.* 2023). Specifically, we added the following

sentence: "*By contrast, an alternative model for human primase initiation proposes that the ancillary domain provides the dominant substrate-binding platform, whereas the catalytic domain must align with it in a catalytically competent configuration to enable dinucleotide formation.*" This revision has been incorporated into the Discussion of the revised manuscript in paragraph 2 on page 28.

Reference for the point-by-point response

1 Boudet, J. *et al.* A Small Helical Bundle Prepares Primer Synthesis by Binding Two Nucleotides that Enhance Sequence-Specific Recognition of the DNA Template. *Cell* (2019). https://doi.org/10.1016/j.cell.2018.11.031

2 Traut, T. W. Physiological concentrations of purines and pyrimidines. *Molecular and Cellular Biochemistry* **140**, 1-22 (1994). https://doi.org/10.1007/BF00928361

3 Wu, P. *et al.* Initial Primer Synthesis of a DNA Primase Monitored by Real-Time NMR Spectroscopy. *J Am Chem Soc* **146**, 9583-9596 (2024). https://doi.org/10.1021/jacs.3c11836

4 Iyer, L. M., Koonin, E. V., Leipe, D. D. & Aravind, L. Origin and evolution of the archaeo-eukaryotic primase superfamily and related palm-domain proteins: structural insights and new members. *Nucleic Acids Res* **33**, 3875-3896 (2005). https://doi.org/10.1093/nar/gki702

5 Zabrady, K., Li, A. W. H. & Doherty, A. J. Mechanism of primer synthesis by Primase-Polymerases. *Curr Opin Struct Biol* **82**, 102652 (2023). https://doi.org/10.1016/j.sbi.2023.102652

6 Bainbridge, L. J., Zabrady, K. & Doherty, A. J. Primase-polymerases: how to make a primer from scratch. *Biosci Rep* **43** (2023). https://doi.org/10.1042/BSR20221986

7 Beck, K., Vannini, A., Cramer, P. & Lipps, G. The archaeo-eukaryotic primase of plasmid pRN1 requires a helix bundle domain for faithful primer synthesis. *Nucleic Acids Res* **38**, 6707-6718 (2010). https://doi.org/10.1093/nar/gkq447

8 Baranovskiy, A. G. *et al.* Mechanism of Concerted RNA-DNA Primer Synthesis by the Human Primosome. *J Biol Chem* **291**, 10006-10020 (2016). https://doi.org/10.1074/jbc.M116.717405

9 Boldinova, E. O. *et al.* The role of catalytic and regulatory domains of human PrimPol in DNA binding and synthesis. *Nucleic Acids Res* **51**, 7541-7551 (2023). https://doi.org/10.1093/nar/gkad507

10 Bergsch, J., Devillier, J. C., Jeschke, G. & Lipps, G. Stringent Primer Termination by an Archaeo-Eukaryotic DNA Primase. *Frontiers in microbiology* **12**, 652928 (2021). https://doi.org/10.3389/fmicb.2021.652928

11 Lipps, G., Weinzierl, A. O., von Scheven, G., Buchen, C. & Cramer, P. Structure of a bifunctional DNA primase-polymerase. *Nat Struct Mol Biol* **11**, 157-162 (2004). https://doi.org/10.1038/nsmb723

Point-by-point response to the reviewers' comments

REVIEWER COMMENTS

Reviewer #1 (Remarks to the Author):

The authors have satisfactorily addressed my concerns in the revised submission.

Reviewer #2 (Remarks to the Author):

Wu et al. have substantially clarified their work in response to reviewer comments. Clearer labeling of newly determined and previously published structures greatly improved interpretation of the figures and conclusions. The mechanism of primase initiation is inherently complex, and if the proposed model is correct, this work provides compelling structural and mechanistic insight into how base-pairing fidelity is enforced during primer synthesis. One outstanding concern regarding the NMR structure remains is described below.

1. One major concern is that the flipped out base, mentioned in the abstract, may be an artifact. As noted in the text, stabilization on the surface is through Met255 and Lys351, as well as hydrophobics in two regions 257-258 and 354-355. Met255 plays a primary role, but Met255 is not an actual primase residue but an artifact of the TEV linker. Is there any evidence, outside of the reported NMR structure, that the T flips out? The K351A mutation that abolished DNA and NTP binding might not have anything to do with stabilizing a flipped out base, but interacts with the DNA as suggested from the previously published structure. One way to prove this result is to show that the DNA flips out in the absence of Met255. Notably. the previously published structure appears not to have the TEV site . Another possibility is that the true linker would also stabilize the flipped out position as the linker does contain some hydrophobic residues (as suggested from 9aan model), but the lack of sequence conservation in the linker would suggest not for such an important functional role. It should be detectable in the chemical shift experiments with the catalytic domain, but I was not able to locate that data. A final possibility is that stabilization of the flipped out base is not actually required, as seen for UDG glycosylase. However, the sugar pucker is sterically held in place in glycosylases, and it's not clear that this would be the case in this NMR structure where the flipping out is near the end of the DNA with few protein contacts. If indeed the flipping is an artifact, then it is formally possible that the observed shifts in GTG binding and the nucleotide binding is also an indirect consequence.

Response: We thank the reviewer for this insightful comment and agree that it is important to determine whether the flipped-out Thy2 observed in our structure represents a genuine mechanistic feature or an artifact arising from the non-native linker sequence. Below, we provide several independent lines of evidence supporting the biological relevance of the flipped-out conformation.

First, the flipped-out Thy2 conformation is observed even in the absence of Met255. The chemical shift perturbations of the HBD in the primase-DNA^{CT}-ATP^{C}-dGTP^{C} complex (Fig. 3a) and the primase^{H145A}- DNA^{CT}-ATP^{C}-dGTP^{C} complex (Supplementary Fig. 1f), both of which contain the native wild-type linker sequence and therefore lack Met255, are essentially identical to those observed in the HBD- DNA^{CT}-ATP^{C}-dGTP^{C} complex (Supplementary Fig. 1e). In addition, the chemical shifts of G3 and T4 are the same in the primase-DNA^{CT}-ATP^{C}-

$dGTP^C$ complex (shown as ATP^C+$dGTP^C$ (0 h) in Supplementary Fig. 4c) and the HBD-DNA^{CT}-ATP^C-$dGTP^C$ complex (shown as 1bp_HBD in Supplementary Fig. 4c). Together, these results demonstrate that Thy2 remains flipped out even in the absence of Met255, indicating that the observed conformation is not caused by the TEV linker artifact.

Importantly, Thy2 is not stabilized solely by Met255 and Lys351. In our structure, the flipped nucleotide also forms hydrophobic interactions with residues in the linker region (Val257 and Val258) and the final helix of the HBD (Val354 and Ile355) (Fig. 1l). Although the linker sequences differ between the isolated HBD and full-length primase, resulting in different chemical shifts for Val257 and Val258 in the two proteins (Response Fig. 1a,b), both constructs exhibit large chemical shift perturbations upon formation of the flipped-out state. Nearly identical perturbations are observed for Lys351, Val354, and Ile355 in the HBD (measured at 298 K) and primase (measured at 313 K), further supporting the conclusion that the flipped-out conformation is maintained independently of Met255 (Response Fig. 1a,b).

The reviewer is correct that our previously published HBD-DNA^{CT}-2ATP structure (PDB: 6GVT) did not contain the TEV cleavage site. However, that construct instead contained a thrombin cleavage site (LVPR↓GS), which also introduced non-native residues. Importantly, we did not observe intermolecular NOEs between the DNA substrate and these residues, and therefore they were not included in the final NMR structure calculations.

We also agree that hydrophobic interactions involving the linker are unlikely to be the primary determinant stabilizing the flipped-out state. Consistent with this interpretation, we observed similar behavior using a different DNA template sequence, DNA^{TC}, instead of DNA^{CT}. In the DNA^{CT} template, ATP acts as the first cognate NTP and cannot base pair with its template nucleotide (Thy2), as shown in our previous HBD-DNA^{CT}-2ATP structure, where the Asn348H and Glu345H resonances are absent in the 2D ^1H-^{15}N HSQC spectra (Response Fig. 2a). These resonances are located within the NTP-binding site of the HBD and become detectable only after formation of a stable single base pair within the HBD (Response Fig. 2b). In contrast, in the DNA^{TC} template, ATP serves as the second cognate NTP and can base pair with its template nucleotide (Thy1), as indicated by the appearance of weak Asn348H and Glu345H signals in the HSQC spectra (Response Fig. 2c). These observations demonstrate that only the second cognate NTP forms a stable base pair within the HBD and further suggest that Cyt2 in the DNA^{TC} template can also adopt a flipped-out conformation. Together, these results indicate that nucleotide flipping can occur even in the absence of optimal hydrophobic interactions with the linker region.

At the same time, the weaker Asn348H and Glu345H signals observed with the DNA^{TC} template (Response Fig. 2c) suggest that hydrophobic interactions modestly enhance stabilization of the flipped-out state. These interactions involve not only the linker region but also hydrophobic residues within the final helix of the HBD, including Val354 and Ile355 (Fig. 1l). Such interactions may additionally explain why many eukaryotic primases preferentially initiate on DNA^{CT} templates[1], as the flipped-out Thy2 in DNA^{CT} may provide a more favorable hydrophobic environment.

Finally, nucleotide flipping may be mechanistically required to create sufficient space for NTP base pairing at the NTP^S site of the HBD. In our previous HBD-DNA^{CT}-2ATP structure, G3 within the GTG motif interacts with the ATP triphosphate at the NTP^S site; however, there is insufficient space for the adjacent 5′ nucleotide (Thy2 in the DNA^{CT} template) to base pair with the bound NTP. In contrast, our current HBD-DNA^{CT}-ATP-dGTP structure shows

that the second 5′ nucleotide (Cyt1 in the DNA^{CT} template) can form a base pair with the NTP at the NTP^{S} site because it occupies a more favorable spatial position. Therefore, the base-pairing interaction between Cyt1 and dGTP, together with stacking interactions involving the GTG motif and the Cyt1-dGTP base pair, likely contributes substantially to stabilization of the flipped-out Thy2 conformation.

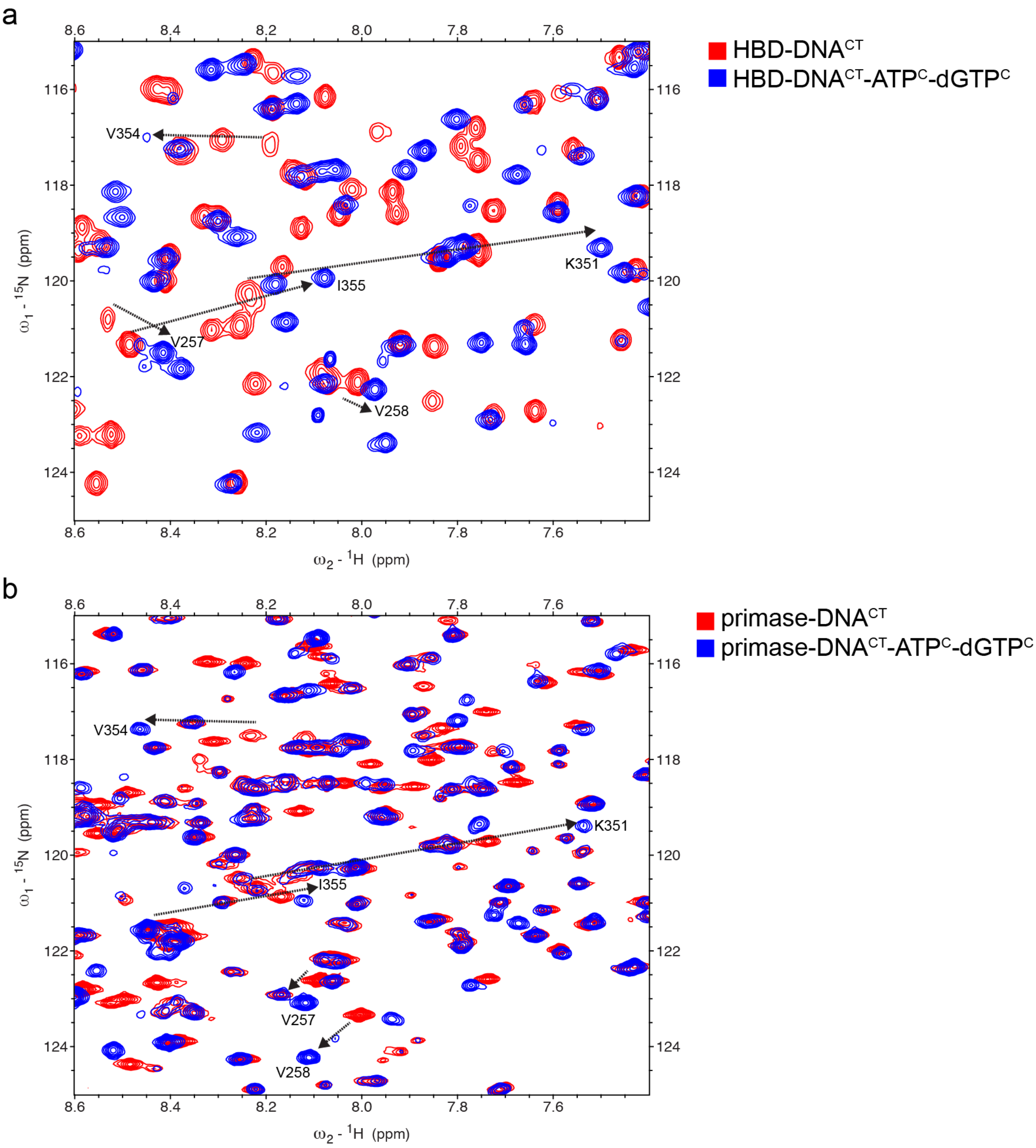

Response Fig. 1 | the flipped-out Thy2 conformation is observed in the absence of Met255. a, Overlaid 2D ^{1}H-^{15}N HSQC spectra of the HBD-DNA^{CT} (red) and HBD-DNA^{CT}-ATP^{C}-dGTP^{C} (blue) complexes, recorded at 298 K. b, Overlaid 2D ^{1}H-^{15}N BEST-TROSY spectra of the primase-DNA^{CT} (red) and primase-DNA^{CT}-ATP^{C}-dGTP^{C} (blue) complexes, recorded at 313 K.

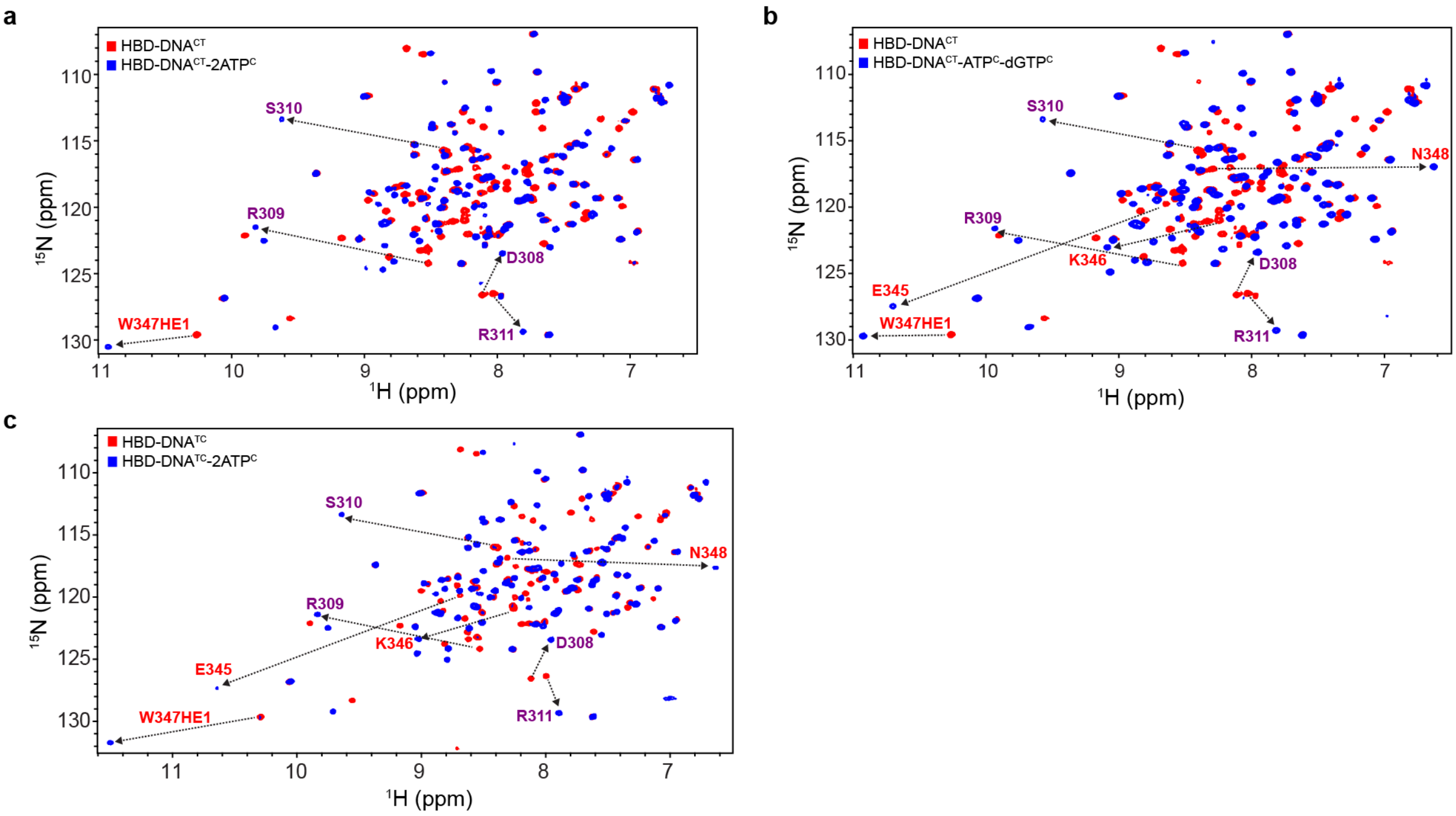

Response Fig. 2 | Formation of a single base pair by the second cognate NTP. a, Overlaid 2D ^{1}H-^{15}N HSQC spectra of the HBD-DNA^{CT} (red) and HBD-DNA^{CT}-2ATP^{C} (blue) complexes. b, Overlaid 2D ^{1}H-^{15}N HSQC spectra of the HBD-DNA^{CT} (red) and HBD-DNA^{CT} -ATP^{C}-dGTP^{C} (blue) complexes. c, Overlaid 2D ^{1}H-^{15}N HSQC spectra of the HBD-DNA^{TC} (red) and HBD-DNA^{TC}-2ATP^{C} (blue) complexes.

2. Along these lines, in contrast to the strong NTP binding site, it appears that the weak NTP binding site is poorly conserved, suggesting it may be found only in specific systems. Is there other evidence that suggests a more universal role?

Response: We agree that the weak NTP-binding site is poorly conserved, suggesting that it may be present only in specific systems or may vary substantially in its detailed structural implementation. In our solid-state NMR spectra, we observe very weak but detectable interactions between the triphosphate moiety of the NTP and residues within the weak NTP-binding site of the HBD (Fig. 1e). These signals demonstrate that, in the pRN1 primase ancillary domain (HBD in this case), a second NTP can indeed bind weakly and transiently.

Notably, many primase ancillary domains in other systems also contain a high density of positively charged residues. This raises the possibility that transient binding of more than one NTP to the ancillary domain may be a more general but previously underappreciated feature of primase function. However, such interactions are expected to be highly dynamic and low affinity.

We also note that capturing such weak and transient binding events is inherently challenging using conventional structural biology approaches such as X-ray crystallography or cryo-EM, which preferentially resolve stable and well-ordered states. As a result, structural evidence for these interactions is currently very limited across primase systems.

Therefore, while it remains uncertain whether all primase ancillary domains universally bind a second NTP, our results suggest that this possibility cannot be excluded and may have been overlooked due to methodological limitations rather than true absence.

3. The authors state on line 87 that they present three structures in this publication. While the methods used to build these hybrid models are now better clarified in the main text, the distinction between the three structures remains potentially confusing for readers. Specifically, only one structure (9RTJ) is a conventional NMR structure determined entirely from experimental NMR restraints, whereas the other two structures are hybrid models built by integrating NMR restraints with crystal. We suggest the authors more explicitly differentiate the hybrid models from the NMR throughout the text.

Response: We thank the reviewer for this helpful suggestion. We agree that the original wording may have led to ambiguity regarding the nature of the three reported structures. In response, we have revised the manuscript to explicitly distinguish the fully NMR-derived structure (PDB: 9RTJ) from the two hybrid models.

Specifically, we now clarify throughout the text that only one structure is determined exclusively from NMR restraints, whereas the other two represent hybrid NMR–crystallography models used to capture distinct functional states during the dinucleotide formation process. We have also revised the relevant section (line 87, highlighted in yellow) to improve clarity and prevent any potential confusion for readers.

4. If you make one mutation on two salt bridges, you can only say that the related salt bridge is not critical, not both, as implied in line 334

Response: We agree with the reviewer that a single D194A mutation can only directly assess the contribution of the D194–R309 interaction, and therefore does not allow a definitive conclusion regarding both salt bridges.

We have revised the manuscript accordingly to avoid overstatement (Line 335, hightlighted in yellow). The text now clarifies that disruption of D194–R309 does not significantly affect primase activity, indicating that at least this interaction is not essential for domain closure, while we refrain from extending this conclusion to the second interaction (G166–R311) in the absence of direct mutational evidence.

5. The authors state that the primary rationale for domain closure is the Watson Crick basepairing. However, they do not mention if the GTG sequence is also required. Their model suggests an extensive interface, providing additional check that the GTG is present.

Response: We thank the reviewer for this insightful comment. To address whether the GTG sequence is required for domain closure in addition to Watson–Crick base pairing, we further examined our structural model of the primase–DNA^{CT}–1bp complex (PDB-IHM: 9AAN) in the closed state.

In this structure, after domain closure, the C1-dGTP base pair is co-bound by both the catalytic domain and the HBD. Importantly, the catalytic domain interacts only with the

phosphate backbone of the GTG motif and does not make specific contacts with either the ribose or base moieties of the GTG nucleotides. This indicates that the GTG sequence is not directly recognized by the catalytic domain and does not itself act as a direct trigger for domain closure.

In contrast, the HBD forms strong interactions with the ribose and base components of the GTG motif. Consistent with our previous biochemical and structural observations, DNA templates lacking the GTG motif fail to properly support NTP binding by the HBD and therefore abolish primase activity[2]. This suggests that the GTG sequence is required to establish a competent NTP-binding configuration in the HBD, thereby enabling correct positioning of the incoming nucleotide for Watson–Crick base pairing.

Therefore, we propose that the GTG motif plays an indirect but essential role in domain closure by enabling productive NTP binding and base pairing within the HBD. Once this correct Watson–Crick base pair is formed, it serves as the primary driving force for domain closure. In this framework, the GTG sequence is necessary for preparing the correct pre-organized state, but the conformational transition to the closed state is primarily triggered by Watson–Crick base pairing geometry rather than direct sequence-specific recognition by the catalytic domain.

6. The chemical shift blots are difficult to read. There's no tick marks making it almost challenging to figure out which residue is showing chemical shifts.

Response: We thank the reviewer for this helpful comment regarding the readability of the CSP plots. In our visualization scheme, residues assigned in both states were assigned positive CSP values, whereas residues unassigned in both states (before and after NTP addition) were assigned negative placeholder values. In addition, residues assigned only in the state before NTP addition were assigned more negative values to distinguish them from fully unassigned residues.

As a consequence of this encoding strategy, CSP plots contain values on both sides of zero, which can lead to overlap with tick marks when the axis is positioned at y = 0. These figures were generated using Microsoft Excel, which does not allow flexible repositioning of tick marks independently of the axis crossing position. As a result, tick marks may overlap with data points in the current implementation.

We apologize for the limitation in the current visualization. In future work, we will consider using more flexible plotting tools that allow independent control of axis and tick placement to improve the clarity of CSP figures.

7. Please state actual K351 mutation on line 318. Please also state which residue was deleted in linker in line 324.

Response: We have revised the manuscript to explicitly state the specific mutation used. The sentence now reads: "mutation of K351 to alanine (K351A)…" instead of the previous generic description of the mutation on line 320.

We have revised the manuscript to specify the exact residue involved in the linker deletion. The sentence now explicitly states that deletion of residue 250 in the linker was used in the referenced study, replacing the previous general description of linker shortening.

8. Please include pdb nomenclature in the supplemental figures.
Response: We have revised the Supplementary Figures to include the corresponding PDB nomenclature, as requested, to improve clarity and facilitate structural interpretation.

Reviewer #3 (Remarks to the Author):

The responses to the reviews are thorough and address all concerns adequately.

1 Bainbridge, L. J., Zabrady, K. & Doherty, A. J. Primase-polymerases: how to make a primer from scratch. *Biosci Rep* **43** (2023). https://doi.org/10.1042/BSR20221986
2 Boudet, J. *et al.* A Small Helical Bundle Prepares Primer Synthesis by Binding Two Nucleotides that Enhance Sequence-Specific Recognition of the DNA Template. *Cell* (2019). https://doi.org/10.1016/j.cell.2018.11.031

Wu et al. report a detailed and exhaustive structural characterization of the molecular mechanism of primer synthesis by the *S. islandicus* primase, serving as a model for DNA primases. The authors have previously characterized certain structural aspects of this system, and describe here how the ancillary domain HBD is not only involved in the recognition of the DNA template but also in the selection of the first two incoming nucleotides and their presentation to the catalytic domain for accurate initiation and elongation of the nascent oligonucleotide. The experiments are carefully designed and expertly performed, the interpretation of the results is sound, and the conclusions are supported by the data. I think this is a very valuable contribution to the understanding of DNA replication.

I suggest that the authors address the following points to improve the clarity of the article:

1. The protein should be identified in the methods section by its Uniprot entry Q54324.
2. The construct spanning the catalytic and the HBD domains is named “full-length” in many instances. This is incorrect and misleading because the large C-terminal domain is not present in the construct studied in this work. Hereafter, I will refer to this construct as “primase” (as the authors label it in Figure 1a).
3. The authors say that the two domains of the primase do not interact in the apo form, but in the crystal structure of the primase (PDB 3M1M, described in reference 21), the two domains do interact. This discrepancy should be clarified.
4. The use of some atomic labels common in the NMR field, used in several of the figures showing NMR spectra, should be explained, so that non-NMR experts are not confused. For instance, I think that “T2Q7” in extended Figure 1c refers to the methyl of T2 in extended Figure 1c, but this is not obvious for the average reader.
5. I think it would be useful to follow the IUPAQ recommendation of naming the amino acids by the three-letter code in the text (not in the figures) so that there is no possible confusion with the same single letters used for nucleotides.
6. Lines 172 and 1185: “Linker” should be “linker”.
7. Line 172: the GHM residues are not TEV cleavage ones (only the Gly is), they are remaining non-native residues after TEV cleavage.
8. Line: “represent” should be “representative”.
9. Line 247 and line 1102: open and closed states are mentioned along the text, but here a “fully” open state is mentioned. Is this a different state from the open one?
10. Line 1103: same as above, with “fully” closed state.
11. The crystal structure of the primase in the apo state does not show magnesium cations. The experimental data supporting the three cations that are actually bound to the primase (three in the 2bp_closed) should be stated, as this information is used in the modelling.
12. Lines 282-284: Some words are missing in this sentence to make full sense.
13. Line 310: “wide” should be “wild”, and “mutants” should be “mutant”.
14. Line 343: “Fig. 5e” should be “Fig 7e”.
15. Explain the colors of the spheres used to represent the different cations in Figure 4.
16. Line 501: Does the abundance refer to the cytoplasm or the nucleoplasm?

17. Line 457: “close” should be “closed”.
18. Line 873: How do the CA and CB chemical shifts report on the formation of a hydrogen bond?
19. Line 944: the quality of these NMR spectra could be illustrated in an extended figure, or a reference could be provided if this kind of measurements have been shown before.
20. Lines 976 and 977: the reported entries in the PDB-IHM could not be found, perhaps because they have not yet been deposited.
21. In relation to the former issue in lines 976-977, the pdb files that are available in the submission are mislabeled: the “1bp” is the two-base pair model, and the “2bp” is the one-base pair model.
22. Line 1050: “ribonulceotide” should be “ribonucleotide”.
23. Lione 1086: “complexed” should be “complex”
24. The CSP plots are designated as “histograms”, but they are not histograms, they are bar plots.
25. Explain the red spheres in extended Figure 4e.
26. In extended Figure 4, the label “DNA_open” is new and confusing. Shouldn´t it be “1bp_open”?
27. Line 1105: “conformaitonal” should be “conformational”.
28. Line 1105: I doubt they are “transition states” (high-energy, low-populated states). Perhaps the authors mean “intermediate states”.
29. In extended figure 7e, the label “wt” would be better changed to “NTP”.
30. Line 1162: Is the R311 resonance from the backbone or the side chain NH group?
31. Line 1164: “the” should come after “at”.
32. Line 1166: It is unclear what “wild type” means when used for NTPs. I think it means non-chemically modified (and does not refer to cognate). Please clarify.
33. Line 1176: “Align” should be “Aligned”.
34. Line 453 and the extended figure 8b: in the scheme, a pi-pi network is presented as being the same in both the pRN1 primase model and the MsCAPP crystal structure, with G3 in pRN1 stacking with both T2 and the ATP at the I-site. This seems to be the case, inspecting the corresponding pdb file of the pRN1 model, but not the pdb of MsCAPP. In the structure of MsCAPP, the corresponding base of the template is stacked with the other bases of the DNA chain, but not with the isolated nucleotides. If Therefore, the scheme does not properly represent the extent of similarity. If the authors think it does, it should be explained what the precise criterion is used to identify which base is stacked to which other base, and explain to what extent the pi-pi network is similar in both models.
35. In relation to the former issue, if the different stacking is maintained, it should be discussed whether it is supported by specific experimental restraints or is driven by the force field used to build or refine the model.
36. In extended Figure 8b, the numbering of the nucleotide sequence of the MsCAPP is different from the numbering in the pdb file 7P9J. Please, clarify

260204primase_review

The primase work by Wu and colleagues carried out NMR work that indicated a novel, unexpected mechanism for initiation of priming, a major question in the field. Their data was complex, with nucleotides binding in unexpected places and a flipped out base in the template strand right where priming should be occurring. The mechanisms revealed by this study are important and would be of interest to Nature Comm readers and the replication and repair fields.

The major concern is the paper's lack of clarity in correlating the structural models (CIFs/PDBs) with the experimental NMR data (e.g., chemical shifts, NOEs, RDCs) used to determine them. The authors appear to shift between discussing determined structures and discussing unassigned or dynamically averaged NMR data, creating significant ambiguity. This makes it challenging for non-specialists in solution NMR (including many structural biologists) to critically evaluate the mechanisms

Comments

1. For readers to immediately assess the significance of this work, please add the organism from which the primase is derived in the abstract and in the Fig. 1 legend. Along the same lines, it would be helpful to add for the general audience that the PRN1 plasmid provides a minimalist primase system. Use of an extremophile model enables many structural insights, but it should be mentioned.
2. The authors refer to 3 structures provided by this work, but only two CIFs are located. HBD-DNA-ATP dGTP seemed to be missing. Figuring out which structure was which was confusing. Adding the assigned pdb deposition ID for structures determined by this paper to figures, including extended data figures and supplementary tables, would improve clarity from which coordinates the figures are derived.
3. There appears to be NMR data where the structures were not determined. How feasible would it be to determine the entire structure, as small changes may be propagated outside of the binding site?
4. Figure 1. It was difficult to reconcile the HBD construct shown in Fig. 1 with the cif file of the full-length protein provided. If the cif file had only the HBD construct, then it would make sense. In the methods, it seems that the authors collected NMR data on both the truncated construct and the full-length. Please clarify or provide individual structures for each independent data set, if the truncated was collected as truncation could alter conformational dynamics in the protein. Throughout it seemed that the authors shifted easily between the two, making it difficult to follow.
5. Fig 1 Metal spheres are not defined at any point The authors have not described that they removed sections of the DNA bases. For the former, it was not until I looked at the cif files directly that I realized.
6. Depth cueing in Fig 1 j makes it look like the lysines are coordinating with the metal.
7. The authors do not make it clear that the structure described in Fig. 2 was previously published in Cell. What is new?
8. To know the generality of the findings,an MSA this archaeal model system compared to the human, would show the conservation of key residues. The authors have an MSA with other archaeal plasmid-based systems that could be used to see close ortholog conservation. It would be helpful for readers to denote any residues mentioned in the text or regions, such as domains and the linker.
9. The reported 5 Å phosphodiester-phosphodiester distance in the flipped-out T2 structure, when modeled with an expected C2'-*endo* sugar pucker, is structurally unusual and potentially incompatible with standard nucleic acid geometry. Please provide the primary experimental evidence.
10. The authors report that they mutated the linker residues to remove hydrophobic, yet they chose lysine, known in protein structures for the hydrophobility of the aliphatic chain. Their choice of mutation did alter beta branching and electron dispersion. The authors argue the biological significance of the flipped out T2. Mutation of the linker partly addressed it, but a more precise mutation (e.g. K351) may be more selective in its effect.

11. In discussion of unexpected preference for pyrimidines, the other side (template) are purines. Could stabilization and stacking of the purines in the template strand be an important consideration?
