# Supplementary material for: Structural and mechanistic insights into primer synthesis initiation by DNA primase
Source: Nat Commun. 2026 Jul 3;17:8299. doi: 10.1038/s41467-026-74862-8 (PMC13469122; doi:10.1038/s41467-026-74862-8)
Supplement: Supplementary file 4 [file 41467_2026_74862_MOESM2_ESM.docx]

**Description of additional Supplementary files**

File Name: Supplementary Data 1

Description:

DNA templates and NTPs used for proofreading and template selection assays. The two nucleotides involved in base pairing are highlighted in red.

File Name: Supplementary Movie 1

Legend: Conformational Changes Upon Single Base-Pair Formation.

File Name: Supplementary Movie 2

Legend: Stepwise Mechanism of Dinucleotide Formation by DNA Primase.
